# Drug repositioning strategy for the identification of novel telomere‐damaging agents: A role for NAMPT inhibitors

**DOI:** 10.1111/acel.13944

**Published:** 2023-10-19

**Authors:** Angela Rizzo, Carmen Maresca, Carmen D'Angelo, Manuela Porru, Serena Di Vito, Erica Salvati, Andrea Sacconi, Francesco Berardinelli, Antonella Sgura, Sergey Kuznetsov, Swapnil Potdar, Antti Hassinen, Antonella Stoppacciaro, Pasquale Zizza, Annamaria Biroccio

**Affiliations:** ^1^ IRCCS—Regina Elena National Cancer Institute Translational Oncology Research Unit Rome Italy; ^2^ Institute of Molecular Biology and Pathology National Research Council Rome Italy; ^3^ IRCCS—Regina Elena National Cancer Institute Clinical Trial Center, Biostatistics and Bioinformatics Unit Rome Italy; ^4^ Department of science “Roma Tre” University Rome Italy; ^5^ Institute for Molecular Medicine Finland (FIMM), University of Helsinki Helsinki Finland; ^6^ Department of Clinical and Molecular Medicine, Sant'Andrea Hospital Sapienza University of Rome Rome Italy

**Keywords:** anticancer therapy, cell death, drug screening, NAMPT inhibitor, oxidative DNA damage, telomeres, TRF2

## Abstract

Drug repositioning strategy represents a valid tool to accelerate the pharmacological development through the identification of new applications for already existing compounds. In this view, we aimed at discovering molecules able to trigger telomere‐localized DNA damage and tumor cell death. By applying an automated high‐content spinning‐disk microscopy, we performed a screening aimed at identifying, on a library of 527 drugs, molecules able to negatively affect the expression of TRF2, a key protein in telomere maintenance. FK866, resulting from the screening as the best candidate hit, was then validated at biochemical and molecular levels and the mechanism underlying its activity in telomere deprotection was elucidated both in vitro and in vivo. The results of this study allow us to discover a novel role of FK866 in promoting, through the production of reactive oxygen species, telomere loss and deprotection, two events leading to an accumulation of DNA damage and tumor cell death. The ability of FK866 to induce telomere damage and apoptosis was also demonstrated in advanced preclinical models evidencing the antitumoral activity of FK866 in triple‐negative breast cancer—a particularly aggressive breast cancer subtype still orphan of targeted therapies and characterized by high expression levels of both NAMPT and TRF2. Overall, our findings pave the way to the development of novel anticancer strategies to counteract triple‐negative breast cancer, based on the use of telomere deprotecting agents, including NAMPT inhibitors, that would rapidly progress from bench to bedside.

## Graphical abstract

In this study, we used an automated high‐content spinning‐disk microscope to identify, within a library of 527 known drugs, the molecules able to impair telomere maintenance, as defined by evaluating TRF2 foci as surrogate marker. The best candidate hit, FK866, was then validated at biochemical and molecular levels, so evidencing a novel role of this NAMPT inhibitor in promoting—through the production of reactive oxygen species—DNA damage and telomere loss, two events inducing tumor cell death.

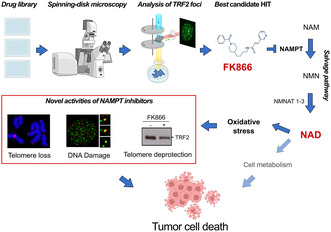

## 1 INTRODUCTION

Telomeres are highly conserved nucleoprotein structures that localize at the very end of eukaryotic chromosomes and play a role in maintaining genomic stability (Chakravarti et al., 2021). At molecular level, human telomeres are constituted by TTAGGG repeats, recognized, and bound by the shelterin complex, a six‐members protein complex, including TRF1, TRF2, and POT1, involved in telomere protection (De Lange, 2018; Ruis & Boulton, 2021; Timashev & De Lange, 2020). Due to their structural organization, telomeres are exposed to the so‐called “end‐replication problem,” a phenomenon that causes a progressive telomere shortening, eventually leading to replicative senescence in normal cells (Harley et al., 1990; Olovnikov, 1973). Telomere erosion acts as a protective barrier against unlimited cell proliferation, and for this reason, cancer cells need to develop telomere length maintenance mechanisms (TMMs) to acquire replicative immortalization. In most human cancers (85%–90%), the main TMM is represented by the upregulation of telomerase, a reverse transcriptase formed by a catalytic subunit (TERT) and an RNA component (TERC) acting as a template for de novo synthesis of TTAGGG repeats (Greider & Blackburn, 1985). In the remaining tumors (10%–15%), telomeres are elongated through a homologous recombination‐based mechanism known as Alternative Lengthening of Telomeres (ALT; Bryan et al., 1997; Recagni et al., 2020).

Based on this knowledge, the TMMs have long been an important target for the development of novel anticancer strategies, some of which—directed against telomerase (e.g., GRVAC1, GV1001, and GRN163L)—have been evaluated in clinical trials (Relitti et al., 2020). Unfortunately, to be effective, telomerase inhibitors need telomeres to shorten up to reach a critical length, a process that can require several cell‐division cycles (Vertecchi et al., 2022). This limitation, together with the ability of certain tumors to develop resistance mechanisms, such as switching from telomerase to ALT TMMs (Recagni et al., 2020), dramatically reduces the possibility of translating telomerase inhibitors into clinical practice.

Evidence produced in the last few years revealed that the targeting shelterin components could represent an alternative strategy to effectively hit telomeres in cancer cells. In this context, pharmacological targeting of telomere repeating factor 1 (TRF1) and 2 (TRF2) has been demonstrated to be a valid strategy to counteract tumor growth (Bejarano et al., 2019; Dinami et al., 2023; El Maï et al., 2021).

Up to date, the approaches used to develop new drugs are mainly based on complex, time‐consuming, and very expensive procedures that require, among the others, the identification and the validation of a target, optimization of an effective lead and, finally, a number of toxicological and preclinical studies, aimed at defining the modalities of a drug formulation and administration before proceeding to clinical studies (Myers & Baker, 2001). In this view, drug repurposing—a method to identify novel therapeutic activities from existing FDA‐approved or clinically used drugs—is assuming a relevant and attractive approach to accelerate the process of drug development. Based on these considerations, we aimed to use the drug repurposing strategy to discover novel antitumoral agents able to promote telomere deprotection. In particular, we performed a drug screening by using a library of molecules selected from a large list of conventional chemotherapeutics, kinase inhibitors, epigenetic drugs, and metabolic modifiers. To ascertain the capacity of these molecules to affect telomere capping in cancer cells, we performed a high‐throughput screening of 527 compounds, in which the shelterin protein TRF2—due to its key role in promoting telomere protection—was evaluated using immunofluorescence staining as a readout parameter. The analysis allowed to identify FK866 (also named Daporinad or APO866), a specific inhibitor of the nicotinamide phosphoribosyltransferase (NAMPT; Hasmann & Schemainda, 2003), as the best hit impairing TRF2 foci. NAMPT is the rate‐limiting enzyme in the biosynthesis of nicotinamide adenine dinucleotide (NAD) from nicotinamide. NAD is essential for redox homeostasis and metabolism and is the substrate for NAD‐consuming enzymes regulating multiple cellular processes, such as DNA repair and gene expression, two fundamental events in supporting energetic need and proliferation of cancer cells (Gasparrini & Audrito, 2022). To satisfy the sustained demand for NAD, several tumor types overexpress NAMPT (Lin, 2022). Starting from these observations, depleting NAD cellular pool by blocking NAMPT activity has emerged as a promising anticancer strategy to affect metabolism and other NAD‐dependent functions. In this view, FK866 has been reported as the first NAMPT inhibitor showing high antitumor activity against leukemia and liver cancer (Galli et al., 2020; Ghanem et al., 2021; Xie et al., 2021).

Here, we demonstrated that FK866 can promote its antitumoral activity by inducing oxidative stress and DNA damage, including at telomere level, both in vitro and in vivo. These findings unveiled a new cross talk between a metabolic pathway and telomere homeostasis, which could improve the rational stratification of patients and suggest new drug combinations in cancer treatment.

## 2 RESULTS

### 2.1 Drug screening to identify telomere‐targeting agents

**FIGURE 1:**
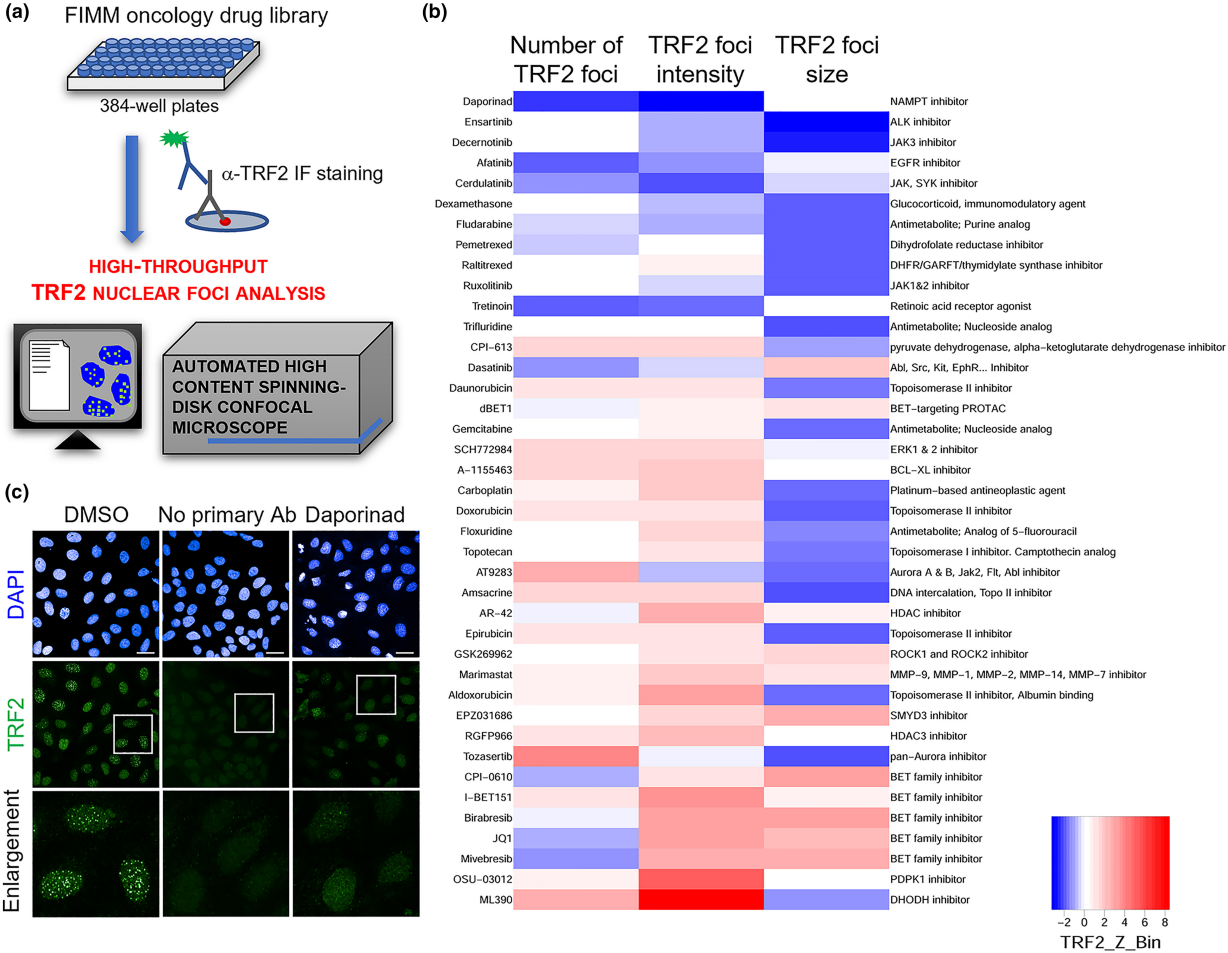
Drug screening to identify telomere‐targeting agents. (a) Schematic representation of the high‐throughput drug screening: 527 compounds, including chemotherapeutic agents, epigenetic modifiers, and kinase inhibitors, were evaluated by the automated spinning‐disk confocal Opera Phenix High Content Screening System for their ability to reduce TRF2 nuclear foci signals in U2OS cells processed for TRF2‐immunofluorescence (IF) assay. (b) Heat map of the drug screening reporting the top effective 40 hits in modulating the indicated parameters of TRF2 foci signals. (c) Representative images with the relative enlargements showing the staining for TRF2 and Hoechst in samples exposed to DMSO as control or FK866 (Daporinad) as the best TRF2‐inhibiting drug identified in the screening, compared with the samples processed in the absence of primary antibody as negative control of IF assay. Scale bars, 20 μm.

The development of new therapeutic approaches aimed at inducing telomere deprotection is nowadays considered a very promising strategy to counteract cancer. Here, taking advantage of an automated high‐content spinning‐disk confocal microscopy, we performed a high‐throughput screening to identify new telomere‐targeting molecules. To this aim, we used a library of experimental and FDA‐approved drugs, known to target cancer‐related pathways (e.g., chemotherapeutic agents, epigenetic modifiers, and kinase inhibitors). Notably, due to the key role played by TRF2 in telomere protection, evaluation of the protein levels was used as a readout parameter for the drug screening (Figure 1a). The analyses were carried out in U2OS, an ALT‐positive osteosarcoma cell line that, thanks to its long telomeres, represent a valid model to easily detect and quantify fluorescent foci derived from the staining of the telomere binding proteins. For the screening, cells were treated with two different concentrations of each drug (selected on the basis of the data available in the literature) and, upon DNA and TRF2 staining, three features, reflecting the number, size, and the fluorescence intensity of nuclear TRF2 foci, were evaluated. To this aim, *z*‐scores were calculated for each of the analyzed readout parameters and drugs showing values below −1 or above 1 were considered as negative and positive TRF2 regulators, respectively (Figure S1). Subsequently, through the application of exclusion criteria, reported in the methods section, it was possible to additionally refine our analyses by scaling down the number of candidate “hits” from the initial 527 to only 40 drugs (28 positive and 12 negative TRF2 regulators; Figure 1b and Table S1). Of note, as telomere uncapping is frequently associated with a reduction of TRF2 levels, we specifically focused on the drugs negatively affecting the TRF2 signal (negative TRF2 regulators). In this view, Daporinad (hereafter FK866) was identified as the most promising candidate drug. Indeed, this well‐known inhibitor of the enzyme NAMPT (NAMPTi) was found to be the most effective drug in reducing both the number and the fluorescence intensity of TRF2 foci with a *z*‐score below −2 (−2.4 and −3.2, respectively) at the 100 nM concentration (Figure 1b,c).

**FIGURE 2:**
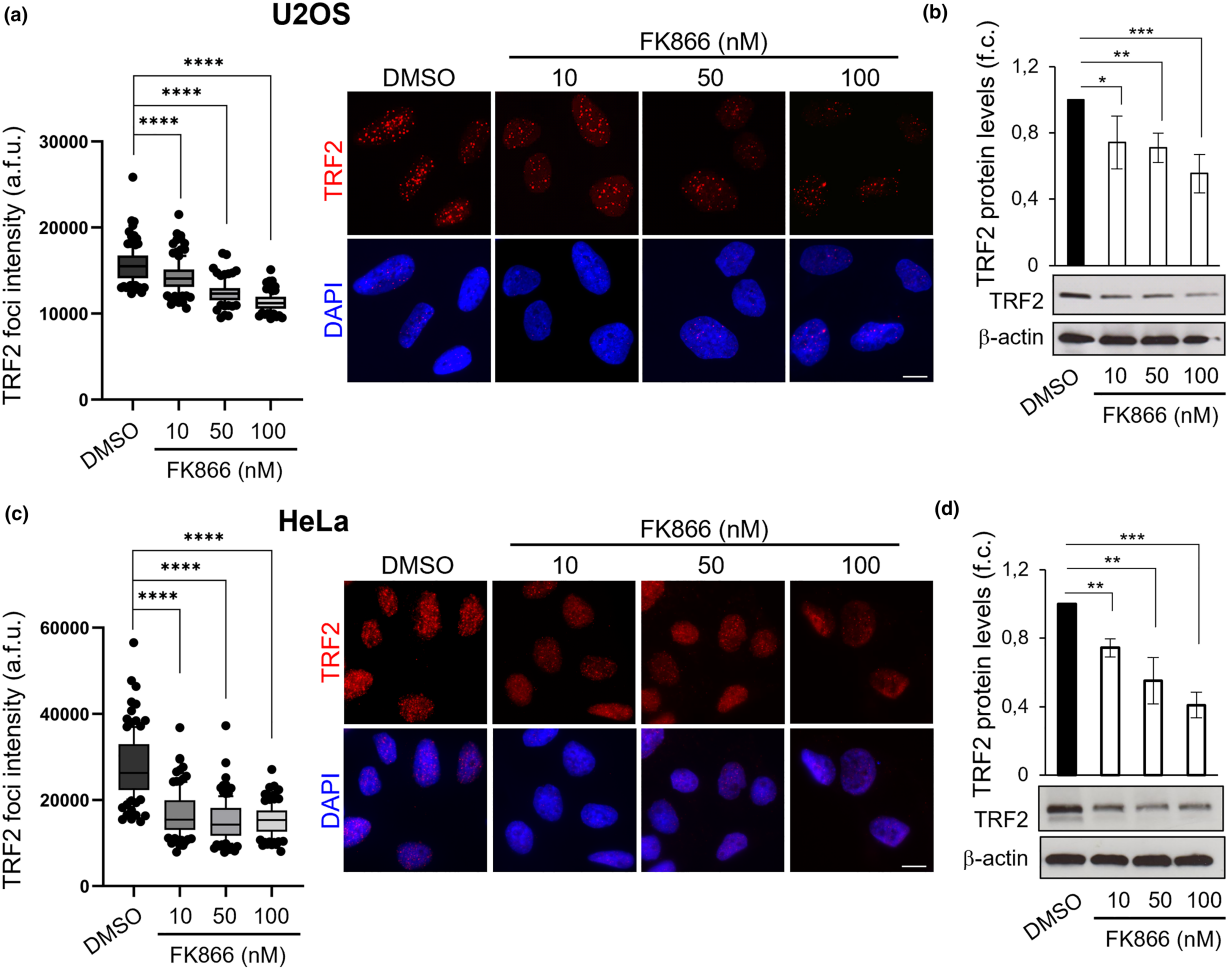
FK866 Treatment reduces TRF2 protein levels. Quantification of TRF2 nuclear foci intensity by IF in U2OS (a) and HeLa (c) cells treated with DMSO as control or the indicated doses of FK866 for 48 h (left panels). Box plots: Middle lines represent median values and the box extends from the 25th to 75th percentiles. The whiskers mark the 10th and 90th percentiles. *p*‐values were calculated on three independent experiments (*n* ≥ 90 nuclei) by using a two‐tailed Mann–Whitney test. Representative images of IF experiments in U2OS (a) and Hela (c) cells are shown (right panels). Scale bars, 10 μm. Quantification of TRF2 protein levels in U2OS (b) and HeLa (d) cells treated as in (a) and (c). Representative WB images are shown below each panel. β‐actin was used as a loading control. Bars indicate means ± SD of three independent experiments. Unpaired two‐tailed *t* test was used to calculate *p*‐values. (*p < 0.05; **p < 0.01; ***p < 0.001; ****p < 0.0001).

To validate the obtained results, the capability of FK866 to negatively affect the expression of TRF2 was then evaluated in independent experiments by both immunofluorescence (IF) and western blot (WB) analysis (Figure 2a,b), using the two drug concentrations already evaluated in the screening plus an additional intermediate dose (10, 50, and 100 nM). Interestingly, treatment with FK866 was found to reduce TRF2 expression in a dose‐dependent manner at all the doses evaluated, and its effect, as demonstrated by real‐time qPCR, could not be explained by alterations of the transcriptional process (Figure S1a). Subsequently, these data were confirmed in other cell lines. Notably, being the U2OS an ALT cell line expressing wild‐type TP53, the analyses were extended to a model of TP53‐mutated ALT cells (MG‐63, Figure S1b) and to two different telomerase‐positive cell lines: HCT116 human colorectal carcinoma (Figure S1c) and HeLa human cervical cancer cells (Figure 2c,d and Figure S1a). Altogether, the obtained results demonstrated that the capability of FK866 to reduce the levels of TRF2 is independent of both the mutational state of TP53 and the mechanisms of telomere maintenance.

### 2.2 Inhibition of NAMPT induces telomere dysfunction

**FIGURE 3:**
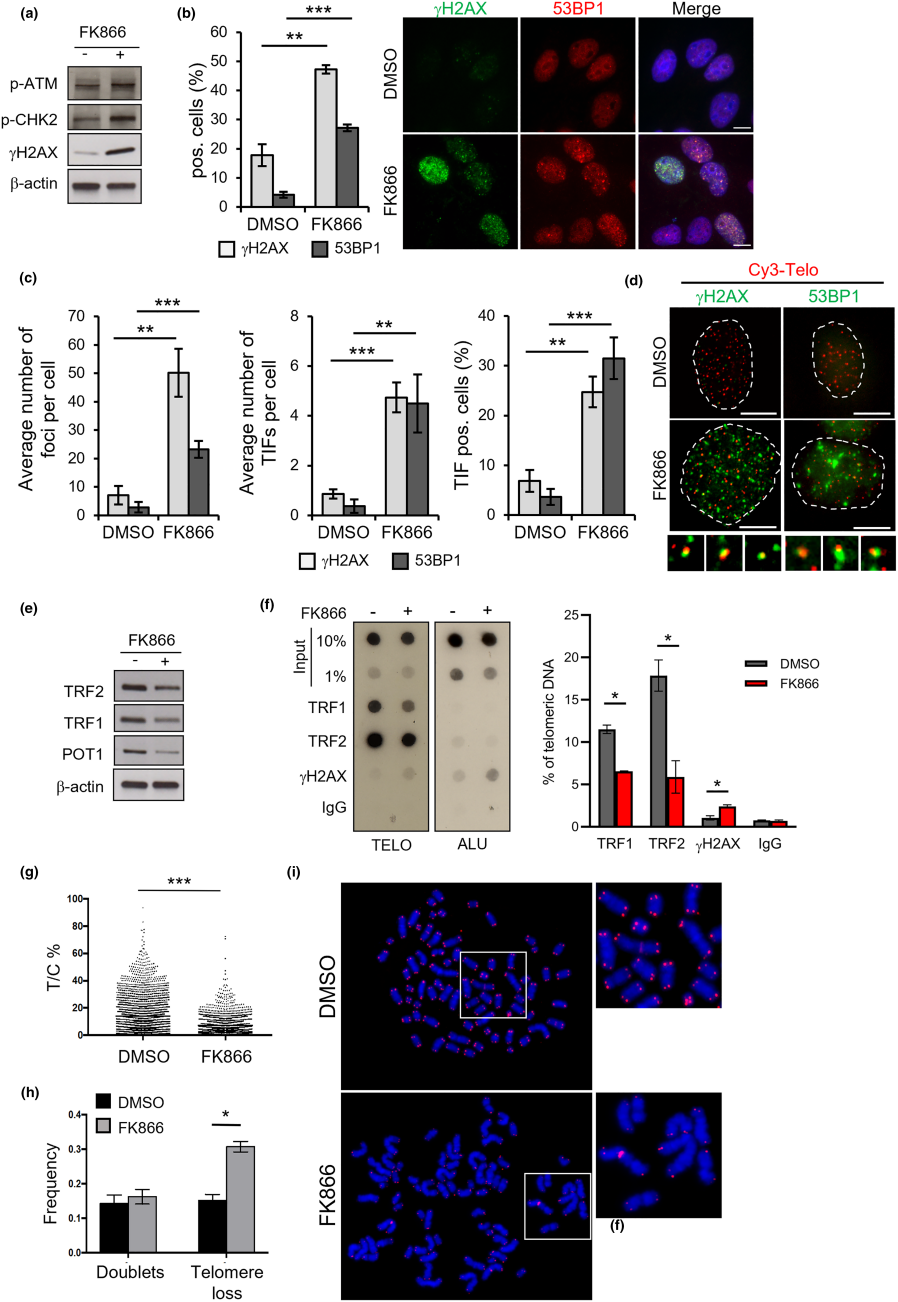
NAMPT inhibitor FK866 induces telomere dysfunction. HeLa cells were treated with DMSO as control or 10 nM FK866 and then processed for the analysis of DNA damage response and telomere integrity. (a) The indicated phosphorylated proteins were evaluated by WB at 48 h after treatment, and β‐actin was used as a loading control. Representative immunoblots of three independent experiments are shown. (b) Histograms in the left panel report the percentage of γH2AX and 53BP1 foci‐positive cells quantified in IF experiments after 48 h of drug exposure. The mean of three independent experiments ± SD is shown (*n* ≥ 90 nuclei). Representative images of IF are shown in the right panel. Scale bars, 10 μm. (c) The average number of foci per cell (left panel), the average number of Cy3‐Telo PNA probe/γH2AX or 53BP1 co‐localizations (TIFs) per cell (middle panel), and the percentage of cells displaying ≥4 γH2AX‐ or 53BP1‐TIFs (right panel) were scored in three independent experiments of IF‐FISH (*n* ≥ 90 nuclei) after 48 h of drug exposure. Bars indicate means ± SD. Unpaired two‐tailed *t* test was used to calculate *p*‐values. (d) Representative images of Telo‐FISH are shown with the enlargements of some colocalizing foci. Scale bars, 10 μm. (e) The indicated shelterin proteins were evaluated by WB at 24 h after treatment with 10 nM FK866, and β‐actin was used as a loading control. (f) After 48 h of drug exposure, chromatin of DMSO‐ or 10 nM FK866‐treated samples was immunoprecipitated with the indicated Abs, and the relative extracted DNA was dot‐blotted and then hybridized with ^32^P‐labeled Telo or Alu probes. Representative experiment is reported in left panel. Histograms in the right panel show the quantification of each ChIP sample normalized to telomeric DNA input. Data represent means ± SD of two independent experiments. Unpaired two‐tailed *t* test was used to calculate *p*‐values. (g) Metaphase spreads of HeLa samples treated with DMSO as control or 10 nM FK866 for 48 h were processed for quantitative telomeric FISH staining (Q‐FISH), and telomere length was calculated as the ratio between the fluorescence of each telomere signal (T) and the fluorescence of the centromere (C) of chromosome 2, used as internal reference in each metaphase analyzed. Data of two independent experiments are expressed as percentages of the ratio T/C. A two‐tailed Mann–Whitney test was used to calculate the statistical significance. In parallel, the frequency of telomere doublets and telomere loss was estimated and reported in (h) as mean ± SD of two independent experiments. (i) Representative images of metaphase spreads with the relative enlargements are shown. Unpaired two‐tailed *t* test was used to calculate the *p*‐values. (*p < 0.05; **p < 0.01; ***p < 0.001).

Based on the obtained results, we evaluated whether the reduction of TRF2 levels was associated with DNA damage in FK866‐treated cells. WB analyses, performed in HeLa cells treated with 10 nM of FK866, revealed a robust activation of the DNA damage response (DDR) pathway (Figure 3a) and evaluated as phosphorylated H2AX (γH2AX), ATM (p‐ATM), and CHK2 (p‐CHK2). In agreement with the WB analyses, IF experiments—performed in HeLa and U2OS cells—evidenced a strong increase in γH2AX and 53BP1—another sensor of DSBs—in response to FK866 treatment (Figure 3b and Figure S3a). In addition, fluorescence in situ hybridization (FISH) assays—performed by co‐staining γH2AX, or 53BP1, with a peptide nucleic acid (PNA) fluorescent telomeric probe—revealed that FK866 (10 nM for 48 h) induces marked levels of DNA damage, a fraction of which (about 15%) is localized at telomeres (Figure 3c,d and Figure S3b).

Since telomeres are particularly hard to be repaired (Fumagalli et al., 2012), accumulation of DNA damage in these regions can lead to an arrest of cell proliferation and/or activation of cell death pathways. In line with this, analyses of cell viability showed that inhibition of NAMPT can induce a dramatic reduction of cell growth (Figure S4a) that, as demonstrated by FACS and WB analyses, is mainly due to the ability of the FK866 to elicit apoptotic cell death (Figure S4b–d).

Then, to define whether cell death would represent the cause or the consequence of telomere dysfunction, effects of FK866 were evaluated in time course experiments. Interestingly the obtained results evidenced that both reduction of TRF2 and accumulation of DNA damage are early events that occur starting from 24 h (Figure S4e,f), when cell death is almost undetectable (Figure S4b–d).

Finally, to evaluate whether FK866‐mediated telomere uncapping goes exclusively through the depletion of TRF2, levels of other shelterin proteins were evaluated. Interestingly, the WB analyses evidenced that FK866—similar to its effect on TRF2—promotes an early reduction of TRF1 and POT1 levels (Figure 3e). Moreover, telomere chromatin immunoprecipitation (telo‐ChIP) assays, performed in the presence and in the absence of FK866, demonstrated that the treatment with the NAMPT inhibitor decreases the amount of TRF1 and TRF2 bound to telomeric chromatin (Figure 3f and Figure S5a) and, in agreement with the previous results, induces an enrichment of γH2AX both at telomeres (Figure 3f) and in nontelomeric heterochromatic regions (Figure S5b–d). In addition, FISH assays evidenced a reduction of the fluorescence in PNA‐labeled telomeres of drug‐exposed cells (Figure S5e,f). Moreover, quantitative FISH (Q‐FISH) analyses, performed on metaphase spreads of cells, clearly demonstrated that FK866 induces early telomere loss events (Figure 3g–i).

**FIGURE 4:**
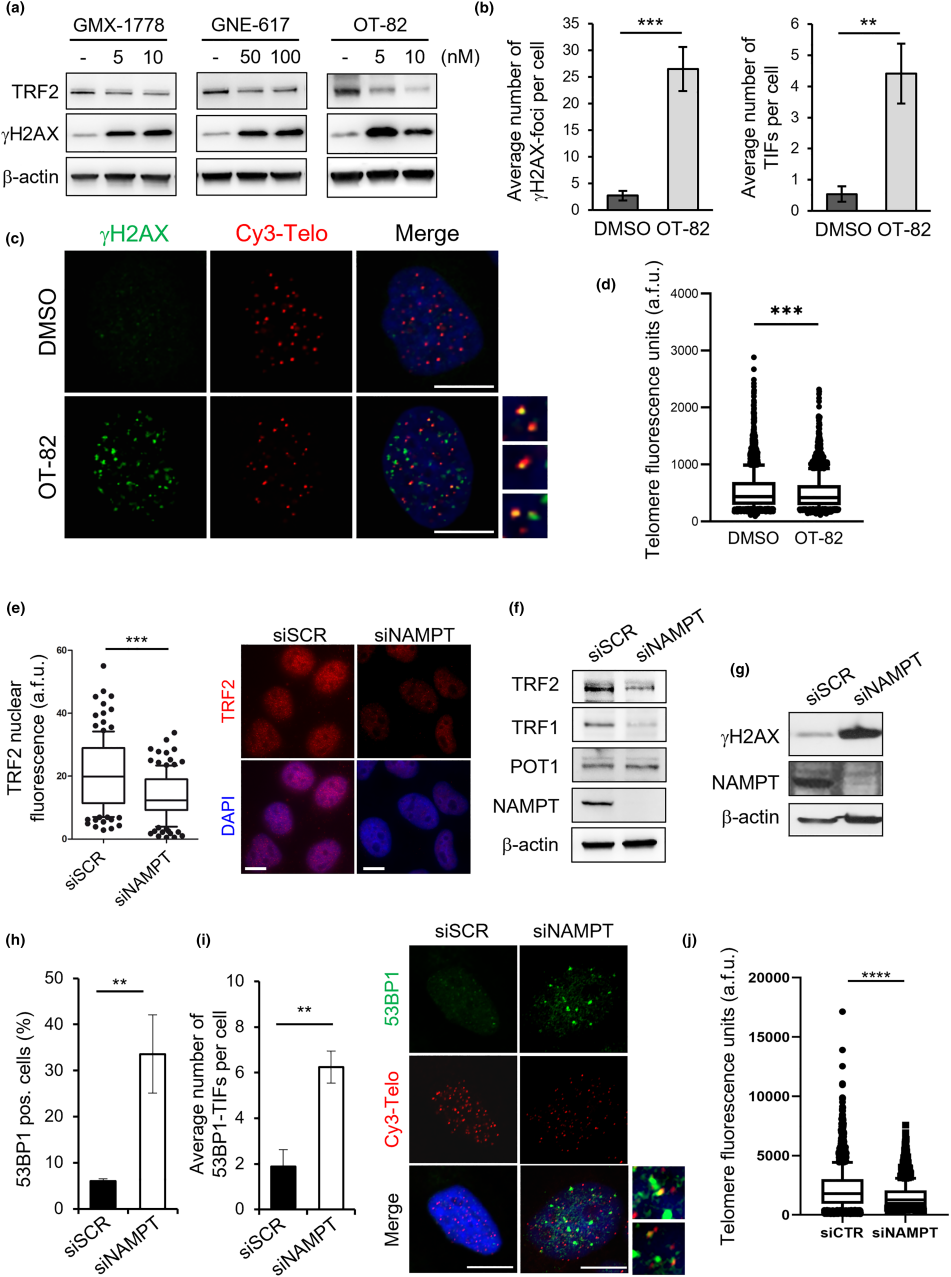
NAMPT inhibition induces telomere dysfunction. (a) HeLa cells were treated for 48 h with the NAMPT inhibitors GMX‐1778, GNE‐617, or OT‐82 at the indicated doses and then processed for WB analysis of TRF2 and γH2AX protein levels. β‐actin was used as a loading control. Representative images of three independent experiments are shown. (b) HeLa cells were treated for 48 h with 5 nM OT‐82 and then processed for telomeric IF‐FISH assay. The average number of γH2AX‐foci (left panel) and TIFs (right panel) per cell was quantified from three experiments (*n* ≥ 90 nuclei). Bars indicate means ± SD. Unpaired two‐tailed *t* test was used to calculate *p*‐values. (c) Representative images of Telo‐FISH are shown with the enlargements of some colocalizing foci. Scale bars, 10 μm. (d) Fluorescence intensity quantification of telomeric signals in HeLa cells treated as in (b) and processed for Telo‐FISH assay (*n* ≥ 90 nuclei). Box plots: Middle line represents the median value of arbitrary telomere fluorescence units (a.f.u.), boxes extend from the 25th to 75th percentiles, and the whiskers mark the 10th and 90th percentiles. Statistical significance from three independent experiments was calculated by two‐tailed Mann–Whitney test. (e–j) HeLa cells were transiently silenced for NAMPT (siNAMPT) or its control counterpart (siSCR) and processed after 72 h of siRNAs transfection for the evaluation of telomere integrity. (e) Quantification of TRF2 nuclear fluorescence signal (left panel) expressed as arbitrary fluorescence units (a.f.u.). Three independent experiments were analyzed (*n* ≥ 60 nuclei). Box plots: Middle line represents median, and the box extends from the 25th to 75th percentiles. The whiskers mark the 10th and 90th percentiles. Representative images of the IF experiments are shown in the right panel. Scale bars, 10 μm. (f and g) The indicated shelterin proteins, NAMPT and γH2AX levels were evaluated by WB, and β‐actin was used as a loading control. (h) Histograms report the percentage of 53BP1 foci‐positive cells quantified in IF experiments. The mean of three independent experiments ± SD is presented. (i) The average number of 53BP1‐TIFs per cell was scored in experiments of IF‐FISH (left panel). The mean of two independent experiments with SD is shown. *p*‐Value was calculated by Unpaired two‐tailed *t* test. Representative images of Telo‐FISH are shown with the enlargements of some TIFs (right panel). Scale bars, 10 μm. (j) Telomere length of the same samples processed as in (i) was measured by quantifying the fluorescence intensity of each telomeric signal from Telo‐FISH assay. Box plot: The middle line represents median arbitrary telomere fluorescence units (a.f.u.), boxes extend from the 25th to 75th percentiles, and the whiskers mark the 10th and 90th percentiles. *p*‐Value was calculated from three independent experiments (*n* ≥ 90 nuclei) by two‐tailed Mann–Whitney test. (**p < 0.01; ***p < 0.001; ****p < 0.0001).

To establish whether the telomere‐targeting activity was a generalizable effect of NAMPT inhibitors, rather than a peculiarity of the FK866, we first extended our analyses to other drugs of the same family. Notably, the treatment of cells with GMX‐1778, GNE‐617, or OT‐82 was found to fully recapitulate the effects of FK866 (Figure 4a–d). Then, to confirm that the effects of the drug were specifically due to the impairment of NAMPT, the same analyses were extended to cells in which the enzyme was transiently silenced. Interestingly, the genetic depletion of NAMPT, analogously to its pharmacological inhibition, caused a significant reduction of both TRF2 and TRF1 (Figure 4e,f), accompanied by induction of telomeric damage (Figure 4g–i) and reduction of telomere length (Figure 4j), indicating that telomere dysfunction is a generalizable event deriving from impairment of NAMPT.

Interestingly, when the dose of FK866 used for the treatment of cancer cells (10 nM) was administered to BJ normal fibroblasts, no effects were observed in terms of telomere dysfunction (Figure S6), indicating that FK866 preferentially targets transformed cells.

### 2.3 NAMPT inhibition induces telomere damage through NAD‐dependent ROS production

As evidenced from the literature, the antitumoral effects of NAMPT inhibitors are tightly dependent on their capability to promote NAD depletion and, consequently, accumulation of reactive oxygen species (ROS). Specifically, treatment with FK866, affecting NAD synthesis, might decrease levels of NADH and NADPH, which act as donors of reductive power for the large majority of ROS‐detoxifying enzymes (Cloux et al., 2019; Hong et al., 2016; Xu et al., 2017). In turn, ROS are known to induce DNA lesions that are mainly dependent on the formation of 8‐oxoguanines (8‐oxoG), a product of guanine oxidation (Suzuki & Kamiya, 2016). Starting from these data and knowing that telomeres are DNA regions particularly rich in guanine residues, it is reasonable to suppose that telomere loss, observed in response to FK866, is a consequence of NAD‐dependent ROS production.

**FIGURE 5:**
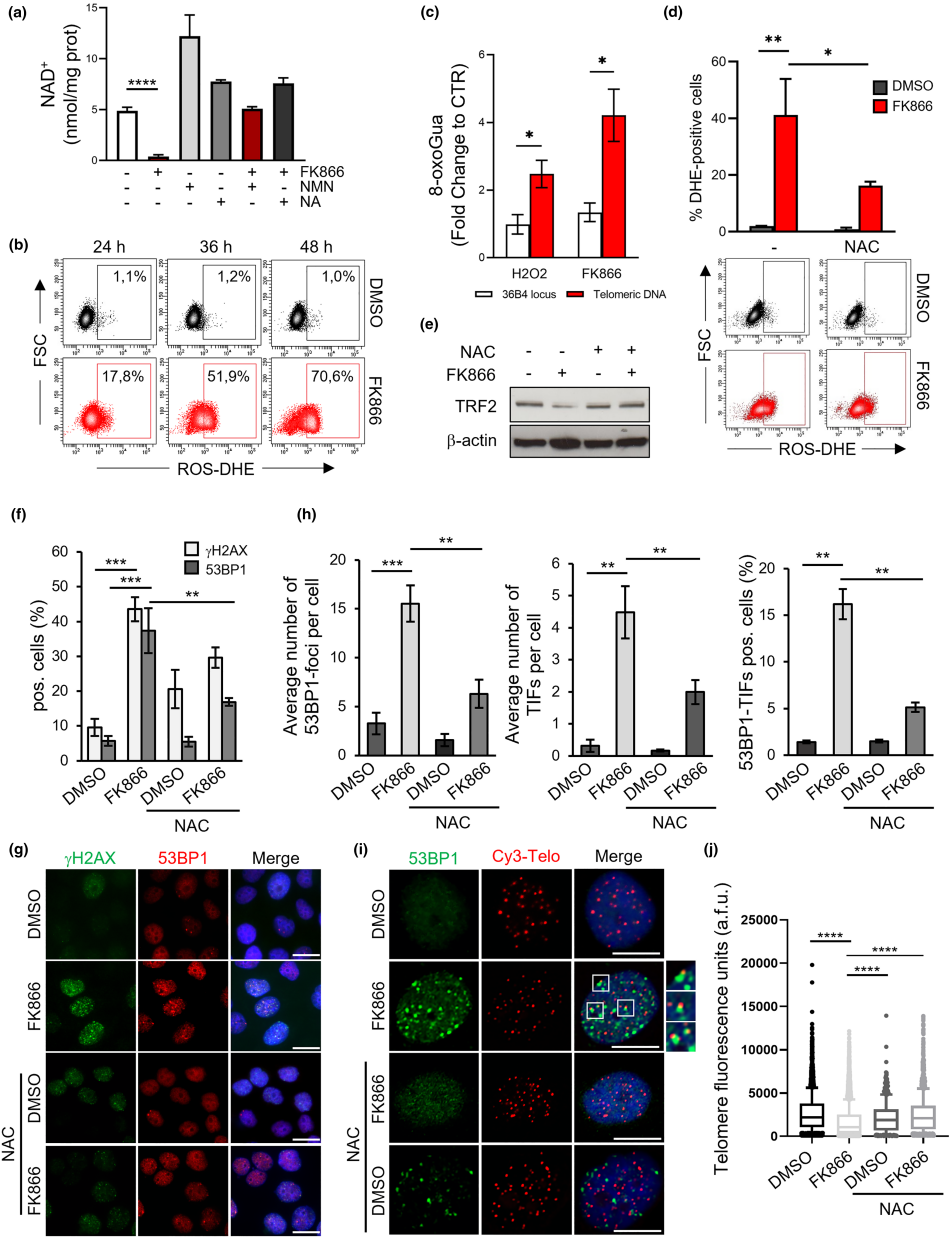
NAMPT inhibition induces telomere damage through NAD‐dependent ROS production. (a) NAD+ content in Hela cells treated for 48 h with 10 nM FK866 or DMSO as control, alone or in combination with 250 μM NMN or 25 μM NA. The mean of three independent experiments ± SD is shown. (b) FACS analysis of ROS‐positive cells, detected with DHE, in HeLa cells treated with DMSO as control or 10 nM FK866 for increasing times. One representative experiment is shown from three independent ones. (c) Histograms report fold increase in oxidized guanine levels (8‐oxoGua) measured from genomic DNA of HeLa cells by qPCR at the 36B4 locus or telomeric DNA after exposure to 200 mM H2O2 (for 1 h) or 10 nM FK866 (for 48 h), compared with untreated samples. 8‐oxoGua were calculated with the ΔCt method (Ct FPG‐digested—Ct undigested). Bars show the mean ± SD from three independent experiments. (d) FACS analysis of ROS‐positive cells in HeLa cells treated for 36 h with DMSO as control or 10 nM FK866 in the presence or the absence of the antioxidant NAC (5 mM). The average of three independent experiments ± SD is reported (upper panel). The FACS analysis of a representative experiment is shown below the histograms. (e) TRF2 levels in the total lysates of samples treated as in (d) were evaluated by WB, and β‐actin was used as a loading control. (f) Histograms report the percentage of γH2AX or 53BP1 foci‐positive cells quantified in IF experiments of HeLa cells treated as in (d). The average of three independent experiments ± SD is presented. (g) Representative images of IF are shown. Scale bars, 10 μm. (h) The average number of 53BP1‐foci per cell (left panel), the average number of 53BP1‐TIFs per cell (middle panel), and the mean percentage of cells displaying ≥4 53BP1‐TIFs were scored in three experiments of IF‐FISH (*n* ≥ 90 nuclei). Bars indicate means ± SD. Unpaired two‐tailed *t* test was used to calculate *p*‐values. (i) Representative images of Telo‐FISH are shown with the enlargements of some colocalizing foci. Scale bars, 10 mm. (j) Telomere length of the same samples processed as in (h) and (i) was measured by quantifying the fluorescence intensity of each telomeric signal from Telo‐FISH assay. Box plot: The middle line represents median arbitrary telomere fluorescence units (a.f.u.), boxes extend from the 25th to 75th percentiles, and the whiskers mark the 10th and 90th percentiles. *p*‐Value was calculated from three independent experiments (*n* ≥ 90 nuclei) by two‐tailed Mann–Whitney test. (*p < 0.05; **p < 0.01; ***p < 0.001; ****p < 0.0001).

To confirm this hypothesis, first, we evaluated whether synthesis of NAD was effectively dependent on NAMPT activity. Interestingly, our experiments demonstrated that FK866, as well as siNAMPT, is able to significantly affect NAD levels (Figure 5a and Figure S7a,b). Moreover, the addition of nicotinamide mononucleotide (NMN)—a NAD precursor synthetized by NAMPT—was found to abolish the inhibitory effects of NAMPT inhibition (Figure 5a and Figure S7a–e), indicating that NAD synthesis goes preferentially (if not exclusively) through the activity of NAMPT (salvage pathway).

An extensive analysis of NAD‐pathway enzymes, performed in HeLa and U2OS cells, evidenced that these two cell lines both express NAPRT (Figure S7f), an enzyme that using nicotinic acid (NA) catalyzes the first step in the biosynthesis of NAD (Preiss–Handler pathway). Based on these data, additional NAD assays were performed in cells co‐treated with FK866 and NA. Of note, NA administration to U2OS cells was unable to rescue NAD levels (Figure S7b), indicating that the NAPTR is not functional in these cells. Conversely, experiments performed in HeLa cells showed an almost complete rescue of NAD (Figure 5a), a result that—in line with literature data—would indicate that the Preiss–Handler pathway is ineffective to counteract the effect of NAMPT inhibitors in these cells, probably due to a limited availability of physiological NA levels (Kirkland, 2009). These results define two different conditions in which the NAMPT inhibitors, completely impairing NAD synthesis, might represent a valid therapeutic tool in cancer treatment.

Then, we evaluated the role of NAMPT inhibition in promoting oxidative stress. Notably, cytofluorometric analyses of dihydroethidium (DHE) levels evidenced a time‐dependent accumulation of ROS in cells treated with FK866 (Figure 5b). Subsequently, drug‐induced oxidative modifications of telomeres were evaluated by a RT‐qPCR‐based assay performed in the presence of formamidopyrimidine DNA‐glycosylase (FPG), a bacterial enzyme that—recognizing and excising oxidized residues—permits to measure the amounts of oxidized guanine in specific DNA regions (O'Callaghan et al., 2011). Interestingly, the results reported in Figure 5c evidenced that FK866 induces oxidative stress and this effect, as demonstrated by the evaluation of 8‐oxoG, is more pronounced at telomeres (about fourfold over its untreated control) than in the 36B4 locus, a DNA sequence with a similar percentage of guanine residues (about onefold over its control). Moreover, comparing the effects of FK866 with those observed upon treatment with hydrogen peroxide (H_2_O_2_, Figure 5c), a generic and potent inducer of oxidative stress, it is possible to conclude that telomeres are DNA regions particularly sensitive to oxidative damaging agents.

Finally, to define the role of ROS in mediating FK866‐dependent telomere uncapping, the most relevant experiments were repeated in the presence of N‐acetylcysteine (NAC), a well‐known antioxidant drug. Interestingly, the obtained results evidenced that 5 mM NAC, a drug concentration that can significatively affect ROS production (Figure 5d), significantly reduces the effects of FK866 on TRF2 expression (Figure 5e), DNA damage response (Figure 5f–i), and telomere length (Figure 5j). Moreover, parallel experiments, performed in NAMPT‐silenced HeLa cells, demonstrated that the genetic depletion of the enzyme promotes a ROS production that, as in the case of the pharmacological treatment, is inhibited by NAC (Figure S7g).

Altogether, these results established a clear and direct link between NAMPT inhibition, ROS production, and telomere dysfunction.

### 2.4 Translational relevance of NAMPT inhibition in breast cancer

Finally, we investigated the translational relevance of our findings. To this aim, we performed a bioinformatic analysis to evaluate the expression levels of TRF2 and NAMPT in a number of publicly available cancer genomic datasets from the cBioPortal platform. Despite the relatively high levels of TRF2 and NAMPT measured in all the evaluated cancer histotypes (Figure S8a,b), the Pearson's correlation analysis revealed that the two mRNAs positively correlate in breast cancer (BC; Figure S8c), suggesting that this kind of tumor would represent, for its molecular characteristics, the best candidate for testing the antitumoral properties of NAMPT inhibitors as telomere‐targeting agents.

**FIGURE 6:**
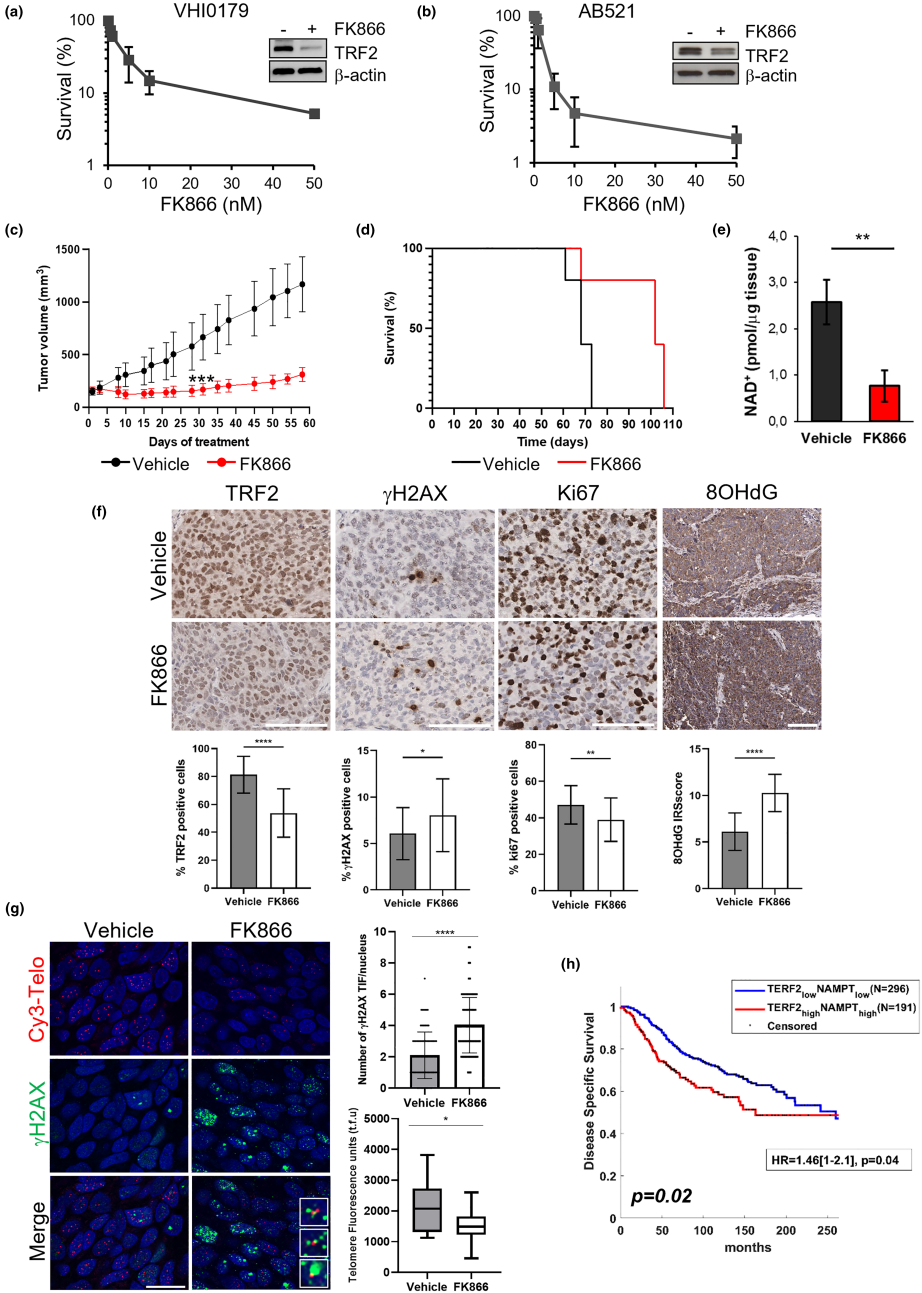
Translational relevance of NAMPT inhibition in breast cancer. Dose‐dependent viability assay of PDTCs derived from triple‐negative breast cancer PDTX VHI0179 (a) and AB521 (b) treated with FK866 for 72 h at the doses ranging from 0.5 to 50 nM. TRF2 protein levels were evaluated by WB at the end of treatment, and β‐actin was used as a loading control. (c) AB521 PDTXs were grafted into NSG female mice. FK866 was administered intraperitoneally (ip, 30 mg/Kg twice daily for 4 consecutive days/week for 3 weeks). Tumor volume was measured at the time points shown on the graphs. Each experimental group included *n* = 8 mice. Errors bars represent SD. *p*‐Values were calculated between treated and untreated tumors at day 31 (Unpaired two‐tailed *t* test, ***p < 0.001). (d) Kaplan–Meier survival curve for mice treated as in (c). Statistical significance was assessed by log‐rank test, *p = 0.020. (e–g) Three mice per group shown in panel (c) were sacrificed at the end of the treatment and tumor samples were excised for ex vivo quantification of NAD^+^ content, immunohistochemical (IHC) analyses, and telomere IF‐FISH. (e) NAD^+^ content quantification in tumor samples from untreated and FK866‐treated mice. (f) In the upper panel, representative images of IHC staining for the indicated markers are reported. Scale bar: 100 μm. The histograms in the bottom panel show the quantification of the IHC assays expressed as the percentage of positive cells to TRF2, γH2AX, or Ki67 staining. The anti‐8‐hydroxyguanine (8‐OHdG) amounts were calculated as immunoreactivity score (IRS). Bars indicate means ± SD. (g) In the left panel, representative images of telomere IF‐FISH are shown. Scale bar: 20 μm. The histograms in the right panel show the average number of γH2AX‐TIFs per nucleus (upper panel) and the quantification of telomere fluorescence units (lower panel). The line in the middle of the box plots denotes a median value, the limits of box represent the interquartile range (25th–75th percentiles), while the whiskers indicate the minimum to maximum values. Unpaired two‐tailed *t* test; *p < 0.05; **p < 0.01; ****p < 0.0001. (h) Disease‐specific survival evaluated by Kaplan–Meier curves on BC patients from the Metabric dataset. Survival of patients, stratified based on TRF2 and NAMPT mRNA expression, is reported. The log‐rank test was used to assess differences between curves. High and low gene expression was defined considering *z*‐scores higher or lower than 0.5, respectively.

Based on these data, we extended our analyses to triple‐negative breast cancer (TNBC), the most aggressive BC subtype that is still orphan of effective therapies. In this view, we first confirmed our most relevant results in a TNBC cell line, the MDA‐MB‐231 (Figure S9a–d). Then, to improve the translational relevance of our findings, we moved in advanced preclinical models of TNBC, here represented by short‐term patient‐derived tumor xenograft cells (PDTCs). These models, thanks to their ability to maintain the main characteristics of the original tumors, are nowadays considered a valuable and cost‐effective tool for drug development (Bruna et al., 2016). In this view, two short‐term PDTCs, VHI0179 and AB521, were generated from TNBC patient‐derived tumor xenografts (PDTXs; Bruna et al., 2016; Groelly et al., 2022) and their response to FK866 was evaluated. Notably, viability experiments, performed by treating the cells with increasing doses of the drug, demonstrated that FK866 induces cell death in a dose‐dependent manner, with an IC_50_ ranging between 1 and 2 nM (Figure 6a,b). Moreover, as demonstrated by WB analyses, the reduced viability observed in the two experimental models correlates, in both the cases, with a robust reduction of TRF2 expression (Figure 6a,b).

In order to test whether our findings would also reproduce in vivo, AB521 were subcutaneously implanted in NOD‐SCID gamma (NSG) female mice and, when tumors reached a volume of 150–200 mm^3^, the animals were randomized and treated intraperitoneally (*ip*) with FK866. Notably, tumors treated with the NAMPT inhibitor showed a significative reduction of the tumor volume, which reached an inhibition of about 70% starting from Day 17, and, more interestingly, a stabilization of the disease was observed and maintained for ~20 days following the end of the treatment (Figure 6c). Moreover, we observed a 100% survival for ~70 days after initiation of treatment (Figure 6d), indicating that FK866 confers a long‐term antitumoral activity. In addition, three mice were sacrificed at the end of the treatment (day 23) and tumor samples were subjected to ex vivo analyses. Interestingly, the treatment with FK866 was found to promote a significant inhibition of NAD levels (Figure 6e), indicating that—despite the co‐expression of NAMPT and NAPRT (Figure S9e,f)—the savage pathway represents the preferential route of NAD synthesis also in this model. Moreover, as demonstrated by immunohistochemical (IHC) analyses performed in mice‐derived tumor sections, FK866 promotes a reduction of TRF2 expression, associated with increased levels of H2AX phosphorylation (γH2AX) and 8‐hydroxyguanine (8‐OHdG), and decreased Ki67 proliferation marker (Figure 6f). Furthermore, IF‐FISH analyses showed that FK866 induces a robust increase in telomere damage associated with a reduction of telomere staining (Figure 6g).

Altogether, these results confirm and reinforce the conclusion that FK866, by inhibiting NAMPT, leads to DNA damage and affects telomeres.

Finally, the clinical relevance of our data was assessed on a cohort of 1215 BC patients from the Metabric dataset. Interestingly, a disease‐specific survival (DSS) analysis in subsets of patients stratified for TRF2 (TRF2^Low^ vs TRF2^High^, *z*_score_ = 0.5) or NAMPT (NAMPT^Low^ vs NAMPT^High^, *z*_score_ = 0.5) mRNA levels did not reveal any significant impact on the clinical outcome when the two variables were considered separately (Figure S10). However, high levels of both TRF2 and NAMPT covariates (TRF2^High^/NAMPT^High^) identified a category of patients with a high risk of relapse/progression (*p* = 0.02, Figure 6h). In accordance with these data, high levels of TRF2 and NAMPT expression (TRF2^High^/NAMPT^High^) were found to correlate with a more aggressive tumor phenotype, as defined by the tumor stage, subtype and expression of estrogen receptor (ER), progesterone receptor (PR), HER2, and p53 (Figure S11). Altogether, these data suggest that NAMPT inhibitors may be a new class of molecules for treating BC and, more specifically TNBCs, an extremely aggressive tumor subclass still in need of effective therapeutic strategies.

## 3 DISCUSSION

Development of effective anticancer therapies represents one of the main goals in oncological research. Indeed, several particularly aggressive malignancies—such as the TNBC—are not very sensitive to standard therapies due to their intrinsic characteristics (Yin et al., 2020), underlying an urgent need for developing novel and more appropriate antitumoral pharmacological approaches.

Drug development is a complex, time‐consuming, and expensive process and, for this reason, new molecules can take several years before entering into clinical practice. In this view, drug repositioning (or drug repurposing)—an investigative procedure aimed at defining new therapeutic opportunities for already existing drugs—is becoming particularly relevant (Pushpakom et al., 2019). Here, we screened a library of 527 experimental and FDA‐approved anticancer drugs to identify molecules with telomere‐damaging activity. To this end, considering the critical role of shelterin proteins in telomere protection (De Lange, 2018), the screening aimed to identify the best candidate hits on the basis of their capacity to impair the expression of TRF2, a key player in the maintenance of telomere and genomic stability. Interestingly, our analyses led to select FK866, a well‐known NAMPT inhibitor (Hasmann & Schemainda, 2003), as the most potent molecule among the tested ones. In agreement with the screening results, performed in telomerase‐negative cells, the capacity of the drug to reduce the expression levels of TRF2 has been confirmed—through independent experiments—also in telomerase‐positive cell lines, so demonstrating that the effect of FK866 on TRF2 is independent from the mechanisms of telomere maintenance.

In contrast to previous studies, whose final goal was the identification of drugs specifically inhibiting TRF1 (Bejarano et al., 2017) or TRF2 (El Maï et al., 2021), we identified here a molecule that, while promoting telomere loss, is able to impair the organization and the activity of the entire shelterin complex. Indeed, digging deeply into the drug's mechanism of action, we demonstrated that FK866 does not target TRF2, per se, but it rather promotes an accumulation of damage in heterochromatic DNA regions, that at telomeres results in rapid shortening events accompanied by loss of telomeric proteins (i.e., TRF2, TRF1, and POT1). Of note, being telomeres hardly reparable DNA regions (Fumagalli et al., 2012), the accumulation of dysfunctions in these chromosomal sites would account—together with the metabolic activity of NAMPT inhibition—for the tumor cell death induced by drug exposure.

At the molecular level, induction of telomere deprotection is dependent on the already known role of the drug in inducing the inhibition of NAMPT, a rate‐limiting enzyme in the salvage pathway, the canonical pathway of NAD biosynthesis. Indeed, the same telomeric effects observed in response to FK866 can be also obtained by using other NAMPT inhibitors (e.g., GMX‐1778, GNE‐617, and OT‐82), as well as, through the genetic depletion of NAMPT, indicating that FK866 activity goes through the inhibition of NAMPT and its effects are not molecule‐specific but generalizable to the entire family of the NAMPT inhibitors.

Despite the missing—at the best of our knowledges—of clear literature data connecting the enzymatic activity of NAMPT to the maintenance of telomere integrity, our data allowed to first associate the telomere loss with an accumulation of reactive oxygen species (ROS) caused by NAMPT inhibition (Cloux et al., 2019; Hong et al., 2016; Xu et al., 2017), a source of oxidative stress that—in line with the literature data—preferentially targets telomeric regions (Coluzzi et al., 2014; Fouquerel et al., 2019; Tan et al., 2020).

The role of ROS in promoting FK866‐induced telomere dysfunctions and cell death was directly assessed by showing that treatment of cells with antioxidant NAC was able to significantly abrogate the activity of FK866. However, knowing that also NAD‐consuming proteins, such as Sirtuins (SIRTs) and the Poly(ADP‐ribose) polymerases (PARPs), play a role in DNA repair and telomere homeostasis (Beneke et al., 2008; Heske, 2020), the contribution of these NAD‐pathway enzymes in promoting tumor cell death cannot be completely ruled out.

Along with the mechanistical data, we also pointed at evaluating the clinical relevance of our results. In this view, we performed a series of bioinformatic analyses aimed at identifying tumors that, expressing high levels of both NAMPT and TRF2, would represent the histotype in which translating our findings. Interestingly, measurements of mRNAs levels, performed on multiple subsets of oncology patients, evidenced that TRF2 and NAMPT positively correlate with each other in Breast Cancer (BC), in which we demonstrated that co‐expression of these two factors correlates with a more aggressive tumor phenotype and, consequently, with a worst prognosis. Based on this clinical evidence, we aimed at translating the obtained results in TNBC, an aggressive subclass of BC that, due to its molecular characteristics, is refractory to canonical endocrine and target therapies. In this view, we first confirmed our most relevant results in the MDA‐MB‐231, a TNBC cell line, and then moved on to test the effects of FK866 treatment in advanced preclinical experimental models, in vivo. Interestingly, the experiments performed with patient‐derived tumor xenografts (PDTXs) proved a marked effectiveness of the drug in promoting reduction of tumor volume associated with a prolonged stabilization of the disease, thus conferring a significant survival advantage to the animals subjected to treatment with FK866.

Altogether, our data identify a novel mechanism through which FK866, a well‐characterized NAMPT inhibitor, promotes its antitumoral activity. Of note, while the canonical mechanism of action of NAMPT inhibitors is dependent on their ability to negatively affect the energy metabolism of the cells, the telomere‐targeting activity of this class of molecules relies on their capacity to induce ROS‐dependent DNA damage. These results pave the way to the development of therapeutic application of NAMPT inhibitors for the treatment of specific tumor subclasses. Indeed, taking advantage of the dual modality through which NAMPT inhibitors can promote their antitumoral activity, patients with such molecular characteristics might significantly benefit from the treatment with this class of drugs.

Efficacy of NAMPT inhibitors in the treatment of cancer aroused the interest of oncological research for a long time. Based on the preclinical results obtained by using this class of molecules, a number of promising drugs—included FK866—rapidly moved on the evaluation in clinical trials. Unfortunately, no one of the tested agents has exceeded Phase II, mainly due to the induction of severe dose‐dependent toxic effects, including thrombocytopenia, retino‐toxicity, and cardiotoxicity. Despite this, the targeting of NAMPT still remains an attractive antitumoral tool, as demonstrated by the continuous efforts employed in the development of new strategies aimed at making safer the use of these molecules, included development of novel and less toxic drugs and definition of strategies to limit the drug doses and/or to bypass their off‐target effects.

Here, in line with these observations, we pointed at extending the obtained results to other NAMPT inhibitors, among which, OT‐82—a recently developed inhibitor—has been selected for its capability to inhibit NAMPT activity without producing cardiac, neurological, or retinal toxicities in mouse models (Korotchkina et al., 2020). On the other side, we demonstrated that the concentration of FK866 that we found to promote DNA damage in cancer is unable to affect normal cells. In addition, the possibility of identifying tumors with a compromised Preiss–Handler pathway would open to the additional possibility of limiting the side effects in normal cells by co‐administrating the NAMPT inhibitors together with NA (Korotchkina et al., 2020). Finally, being the dysfunctional telomeres particularly sensitive to DNA‐damaging agents, such as ionizing radiations (Goytisolo et al., 2000; Wong et al., 2000), it is reasonable to propose NAMPT inhibitors in combination with other anticancer drugs. Interestingly, among the advantages of applying a such combinatorial therapeutic scheme, it should also be considered a possibility to scale down the concentration of the drugs thereby limiting the risk of toxic effects (Galli et al., 2020).

In conclusion, this study discovered a previously unreported telomere‐targeting mechanism through which NAMPT inhibitors can promote their antitumoral activity. Notably, unveiling a cross talk between a metabolic pathway and the homeostasis of telomeres in cancer cells, these findings could lead to an improvement in the stratification of cancer patients and could suggest new therapeutic strategies for the treatment of certain cancers still missing effective therapies, including the TNBC.

## 4 EXPERIMENTAL PROCEDURES

### 4.1 High‐throughput drug screening

Drug screening was performed with U2OS cells using the FIMM oncology drug library FO5A (Table S2). Eight thousand cells/well (25 μL) were seeded into 384‐well imaging plates (CellCarrier‐384 Ultra, Perkin Elmer). The cells were incubated in the presence of the indicated drugs for 72 h, and TRF2 foci were evaluated by immunofluorescence microscopy (see experimental procedures for details). Images were acquired with a spinning‐disk confocal Opera Phenix High Content Screening System (Perkin Elmer). Thirty fields in each well were imaged using a 40× water immersion objective (NA 1.1, 0.62 mm free working distance, 320 × 320 μm field of view; Zeiss, Germany) and analyzed with the Harmony 4.9 software (Perkin Elmer). Nuclei were labeled with DAPI, and 24 features of the stained objects related to the nuclear and TRF2‐foci number, intensity, and morphology were evaluated. Resultant values were expressed as a *z*‐score from the mean of control wells. *Z*‐scores were separately calculated for 6 bins in accordance with the number of cells per well to account for a gradual increase in standard deviation as the number of cells per well (reflecting cell viability) decreased. Drugs with a *z*‐score below −1 were considered negative regulators of TRF2 foci, while those with a *z*‐score above 1 were considered positively affecting TRF2 foci. Out of 527 drugs, 57 molecules with a *z*‐score below −1 or above 1 for any parameter at any concentration were initially identified as candidate “hits.” Next, those drugs for which the number of nuclei per well was below 250 were excluded as unreliable. Candidates with inconsistent dose–response values, that is, when the effect at a higher of two doses was less than the one at a lower dose, were also excluded. After these criteria were applied, 40 drugs positively or negatively affecting one of three key features of TRF2 foci—number, size, and fluorescence intensity—were identified.

### 4.2 DNA extraction and relative quantification of oxidized bases by quantitative PCR

Genomic DNA (gDNA) was extracted from cultured cells using the NucleoSpin DNA RapidLyse Kit (Macherey‐Nagel) following the manufacturer's instructions. To quantify the relative accumulation of oxidative bases at telomeric loci, the procedure described in O'Callaghan et al. (2011) was performed. Briefly, for each condition 400 ng of gDNA was incubated with 12 units of FPG (New England BioLabs) in 1× NEB buffer at 37°C overnight. The quantitative real‐time amplification (qPCR) was performed on 40 ng of digested or undigested gDNA in a 1xSYBR Green master mix (Applied Biosystems [AB]), including specific telomeric or 36B4 primers (Table S3). Following qPCR amplification, profiles of FPG‐treated and FPG‐untreated samples were compared by calculating the relative ΔCT (CT digested – CT undigested).

### 4.3 PDX‐derived tumor cells (PDTC)

Short‐term PDTC culture was obtained from two TNBC patient‐derived tumor xenografts (PDTX; VHI0179 and AB521) as described in Bruna et al. (2016). Briefly, fresh patient‐derived tumor xenografts (PDTXs) were thawed and dissociated, to obtain a single cell suspension, by combining mechanical and enzymatic dissociation. The tumor dissociation was performed using GentleMACS Dissociator and the human tumor dissociation kit (Cat ID 130–093‐235; Miltenyi Biotec) according to the manufacturer's instructions. Single cells were plated at density of 5 × 10^3^ cells (96‐well plates) and treated after 24 h with FK866 for 72 h. The inhibition of cell viability was measured by CellTiter‐Glo® Luminescent Cell Viability Assay (cod. G7570; Promega) in accordance with the manufacturer's instructions.

### 4.4 In vivo xenograft experiments

All animal procedures were performed following the national and international directives (D.L. March 4, 2014, no. 26; directive 2010/63/EU of the European Parliament and European Council; Guide for the Care and Use of Laboratory Animals, U.S. National Research Council, 2011; Animal Research guidelines Reporting of In Vivo Experiments (ARRIVE) guidelines) and approved by the Italian Ministry of Health (authorization n. 607/2019‐PR, released on 07‐08‐2019).

Mice were maintained in a barrier facility on high‐efficiency particulate air HEPA‐filtered racks and received food and water ad libitum.

To generate PDTXs, tumor fragments (15–20 mm^3^) derived from AB521, a triple‐negative breast tumor, were coated in Matrigel and implanted by a small incision in a subcutaneous pocket made in one side of the lower back into NOD.Cg‐PrkdcSCID IL‐2R null (NSG) female mice (Charles River Laboratories). When the tumor reached 150–200 mm^3^, mice were randomly divided into two groups and treated intraperitoneally (ip) with vehicle and FK866 at 30 mg/mouse twice at days for 4 consecutive days/week for 3 weeks as previously described in Cea et al. (2012). Each experimental group included eight mice. At the end of treatment (day 23), three mice were sacrificed and tumors were excised for NAD^+^ quantification and histologic assays. The mice were sacrificed, for ethical reason, when the tumor reached a volume of about 1700 mm^3^.

### 4.5 Statistics

Experiments were replicated three times, with few exceptions as specified in the figure legends, and the data were expressed as means ± standard deviation (SD). GraphPad Prism 6 was used for the statistical analysis, and the differences between groups were analyzed by the unpaired Student's *t* test or two‐tailed Mann–Whitney test. Differences were considered statistically significant for *p < 0.05; **p < 0.01; ***p < 0.001; ****p < 0.0001.

## AUTHOR CONTRIBUTIONS

A.R. contributed to design and analysis of experiments; validation; investigation; methodology; and writing—original draft preparation and editing. C.M. contributed to analysis; validation; and methodology. C.D. contributed to FACS analyses and methodology. M.P. contributed to in vivo experiments and methodology. S.D.V. contributed to IHC analysis and methodology. E.S. contributed to investigation and writing—original draft. A.Sa. contributed to bioinformatic analysis of patient's datasets. F.B. and A.Sg. contributed to telomeric aberrations analysis and methodology. S.K., S.P., and A.H. contributed to high‐throughput drug screening analysis and methodology. A.St. contributed to IHC analysis and supervision. P.Z. contributed to investigation; supervision; and writing—original draft and editing. A.B. contributed to conceptualization; supervision; funding acquisition; investigation; and writing—original draft and editing. All authors read and approved the final manuscript.

## CONFLICT OF INTEREST STATEMENT

None declared.

## Supporting information


Data S1.

Figure S1–S11.

Table S1.

Table S2.

Table S3.

## Data Availability

The data that support the findings of this study are available from the corresponding author upon reasonable request.
