# Supplementary material for: Drug repositioning strategy for the identification of novel telomere‐damaging agents: A role for NAMPT inhibitors
Source: Aging Cell. 2023 Oct 19;22(11):e13944. doi: 10.1111/acel.13944 (PMC10652301; doi:10.1111/acel.13944)
Supplement: Supplementary file 1 — Data S1. [file ACEL-22-e13944-s004.pdf]

Supplemental experimental procedures

Cell lines, culture conditions and transfection

Human cervical cancer cells (HeLa), human osteosarcoma cells (U2OS), triple negative breast cancer cells (MDA-MB-231) and human normal fibroblasts (BJ) were purchased from ATCC and grown in high glucose Dulbecco′s Modified Eagle′s Medium (DMEM, EuroClone, Italy), supplemented with L-glutamine, Penicillin/Streptomycin and 10% fetal bovine serum (FBS; Thermo Fisher Scientific - Gibco, Waltham, Massachusetts, USA), in humidified incubator at 37 °C with 5% CO_2 and atmospheric O_2 concentration. Cell lines were authenticated and tested for Mycoplasma. All were Mycoplasma free.

For transient RNA interference experiments a control scrambled siRNA (siSCR, sc-37007; Santa Cruz Biotechnology; Dallas, Texas, USA) or siNAMPT (sc-45843, a pool of 3 target-specific 19-25 nt siRNAs; Santa Cruz Biotechnology) were transfected into cells using INTERFERin (Polyplus, New York, NY, USA) according to the manufacturer's instructions.

Reagents and treatments

NAMPT inhibitors FK866, GMX1778, GNE-617 and OT-82 (Selleckchem, Houston, Texas, USA) were dissolved in DMSO for *in vitro* experiments and used at the indicated dilutions and exposure times. In the case of *in vivo* experiments, FK866 was dissolved in 5% DMSO + 40% PEG 300 + 5% Tween 80 + 50% ddH_2O. To induce oxidative DNA damage, cells at about 80% of confluence were treated with 200 μM H_2O_2 (Sigma-Aldrich, St. Louis, Missouri, USA) in serum-free DMEM for 1 h. N-acetyl-L-cysteine (NAC), Nicotinic acid (NA) and β-Nicotinamide mononucleotide (NMN; Sigma-Aldrich) were freshly dissolved in phosphate buffered saline (PBS) and administered to cells at a final concentration of 5 mM, 25 μM and 250 μM, respectively, alone or in combination with 10 nM FK866.

Immunofluorescence (IF).

IF experiments were carried out as reported in (Salvati et al., 2007). For immunostaining, the following primary and secondary antibodies were used: mAb anti-TRF2 (clone 4A794; Millipore, Bedford, MA), anti-γH2AX (clone JBW301; Millipore) and pAb anti-53BP1 (Novus Biologicals, Italy); goat anti-rabbit and anti-mouse IgG (H+L), F(ab')2 Fragment (Alexa Fluor 488 or 555 Conjugate; Cell Signaling Technology, Beverly, MA, USA). Nuclei were stained with 4',6-diamidino-2-phenylindole (DAPI; Sigma-Aldrich). Fluorescence signals were acquired at 63x magnification by using the Leica DMi8 microscope equipped with the Leica DFC 350FX camera (Leica, Solms, Germany).

Telomere fluorescence in situ hybridization (Telo-FISH)

To evaluate the telomeric DNA damage IF staining was combined with FISH assay. After fixation and permeabilization, cells were blocked for 1 h with 3% FBS in PBS and incubated with the primary antibody (mAb anti-γH2AX or pAb anti-53BP1) overnight at 4 ºC. After two washes with PBS and incubation in the relative Alexa Fluor-488 secondary antibody, cells were fixed in 4% formaldehyde in PBS for 2 min, washed three times with PBS and subjected to standard telomere DNA FISH. In detail, the glass coverslips containing cells were dehydrated by successive incubations in 70%, 90%, and 100% ethanol; allowed to air dry and then mounted on slides in the presence of the Cy3-labeled $(CCCTAA)_3$ peptide nucleic acid (PNA) telomere probe (Panagene, Daejeon, South Korea) to be denatured at 80 °C for 3 min and hybridized for 2 h at RT in a humid chamber. Subsequently, the slides were washed under agitation, twice with FISH solution (70% formamide, 10 mM Tris pH 7.2, 0.1% BSA) for 15 min and three times with 0.08% Tween-20 in TBS at RT. Finally, slides were stained with DAPI and dehydrated as above mentioned. Fluorescence signals were acquired at 63x magnification by using the Zeiss Laser Scanning Microscope 510 Meta (Zeiss, Germany). For Telomere-Induced Foci (TIFs) analysis, at least 30 γH2AX or 53BP1-positive nuclei on a single plane were scored per experiment. Cells with at least four telomere-γH2AX (or -53BP1) colocalizing

nuclear spots were considered TIF positive. To quantify the intensity of telomeric signals, 30 interphase nuclei for each sample in triplicate were analyzed using spot IOD analysis (TFL-TELO) software. A Mann–Whitney was used to calculate statistical significance.

Metaphase spreads preparation

Chromosome spreads were obtained following standard procedures. Briefly, colchicine $5x10^{-6}$ M was added to the cells 4 h before the finalization of the culture. Cells were then incubated with 75 mM KCl hypotonic solution for 20 min at 37 °C, and subsequently fixed in freshly prepared Carnoy solution (3:1 methanol/acetic acid (v/v)). Cells were then dropped onto slides, air dried, and utilized for cytogenetic analysis.

Quantitative telomeric FISH

Centromere calibrated Q-FISH staining was performed as previously described (Surace et al., 2014). Briefly, 48 h after the treatment with 10 nM FK866 or DMSO as control, slides were post-fixed in 4% formaldehyde for 2 min and dehydrated with an ethanol series. Slides and probes (Cy3-telomeric and -centromeric of chromosome 2) were co-denatured at 80 °C for 3 min and hybridized for 2 h at RT in a humidified chamber. After hybridization, slides were washed in 70% formamide and then dehydrated with an ethanol series and air-dried. Finally, samples were counterstained with DAPI and sealed using Vectashield antifade solution (Vector Laboratories, Burlingame, CA, USA). Images were captured at 63× magnification with an Axio Imager Z2 (Carl Zeiss, Jena, Germany) equipped with a charge-coupled device camera, and the telomere length was analyzed with ISIS software (MetaSystems, Altlussheim, Germany). The software calculates telomere lengths as the ratio between the fluorescence of each telomere signal and the fluorescence of the centromere of chromosome 2, used as internal reference in each metaphase analyzed. Data were expressed as a percentage (T/C %) (Perner et al., 2003). Telomere length analysis was performed on at least 30 metaphases per sample

in two independent experiments. Telomere loss and telomere doublets were analyzed in at least 100 metaphases for each sample in two independent experiments.

Western blotting

Total cell lysates were prepared using a proper buffer (50 mM Tris-HCl pH 7.5, 5 mM EDTA, 250 mM NaCl, 0.1% Triton) containing protease and phosphatase inhibitors (Thermo Fisher Scientific). Samples were sonicated (Sonopuls HD2070.2, Bandelin; Germany) followed by centrifugation at 16.000 × g for two min. Supernatants were recovered and used for Western blotting analysis. The following antibodies were used: mAb anti-TRF2 (clone 4A794; Millipore); pAb anti-TRF1 (N-19; Santa Cruz Biotechnology); pAb anti-POT1 (Abcam, Cambridge, UK); pAb anti-p-ATM (Ser1981; Cell Signaling Technology); pAb anti-p-Chk2 (Thr68; Cell Signaling); mAb anti-phospho-histone H2A.X (γH2AX, Ser139, clone JBW301; Millipore); mAb anti-PARP1 (Mab 551025; BD Pharmingen, San Jose, CA, USA); mAb anti-NAMPT (E-3; Santa Cruz Biotechnology); mAb anti-NAPRT (B-8; Santa Cruz Biotechnology); mAb anti-SIRT1 (B-10; Santa Cruz Biotechnology); mAb anti-β-actin (Sigma-Aldrich). The following secondary antibodies were used: goat anti-mouse or anti-rabbit immunoglobulin G (IgG)-horseradish peroxidase conjugated antibodies (Biorad, Hercules, CA, USA). The intensity of protein bands was quantified by densitometric analysis using ImageJ software.

Cell proliferation

HeLa and BJ cells were seeded in 24 well plates at a density of 1×10^4 and 2×10^4 cells, respectively, per well. After 24 h from plating, FK866 was added to the medium at concentrations ranging from 2,5 to 50 nM and cell growth was monitored by Incucyte® S3 Live-Cell Analysis System (Essen BioScience, Ann Arbor, MI). Images were automatically captured every 12 h under a phase-contrast microscope (10× magnification). Viability was assessed by comparing the cell confluence between groups using IncuCyte S3 Image Analysis Software (Essen Bioscience). Additionally, cell growth

was evaluated in HeLa cells seeded in 6 well plates at a density of 1×10^5 cells per well and counted daily using the automated cell counter Countess II (Invitrogen, Carlsbad, CA, USA).

RNA isolation, quantitative real-time PCR (qRT-PCR)

Total RNA was isolated from the cells using TRIzol reagent (Invitrogen). The mRNA Reverse transcription (RT) was performed using the QuantiTect Reverse Transcription Kit (Qiagen, Hilden, Germany), according to the manufacturer's instructions. cDNA was evaluated by SYBR Green (Applied Biosystems) qRT-PCR method using a standard protocol in the QuantStudio 6 Flex Detection system. The list of primers is reported in the Table S3.

Chromatin Immunoprecipitation (ChIP)

Hela cells exposed for 48 h to 10 nM FK866 or DMSO as control vehicle, were fixed with 1% of formaldehyde. Nuclei were isolated by using a Dounce homogenizer and lysed with SDS lysis buffer. Next, the lysates were sonicated to generate fragments averaging 0.5 to 1 kb (Diagenode Bioruptor Inc., NXT-Dx Belgium). The following antibodies were used for ChIP: pAb anti-TRF2 (Novus Biologicals); pAb anti-TRF1 (Abcam); mAb anti-γH2AX (Millipore). IgG Rabbit (Bethyl Laboratories, Montgomery, Texas, USA) were used as negative control. After the elution from immuno-complexes and the reverse of cross-linking, the DNA was precipitated, blotted onto Hybond-N membrane (Amersham), and the telomeric repeat sequences were detected through hybridization with a radiolabeled-telomeric probe. A nonspecific probe recognizing Alu sequences was also used. To verify that an equivalent amount of chromatin was used in the immunoprecipitates, samples representing the 1% and 0.1% of the total chromatin (input) were included in the telo-blot. The filter was exposed to a PhosphorImager screen (Bio-Rad), and the signals were measured using ImageJ software. Alternatively, DNA obtained from ChIP was analyzed by SYBR Green (Applied Biosystems) qRT-PCR method in the QuantStudio 6 Flex Detection system. The list of primers is reported in Table S3. ChIP analysis was performed using the percent Input method.

NAD^+ quantification

NAD^+ levels were determined on tumor tissue samples and culture cells using the Q-NAD Tissue/Cell NAD+ and NADH assay kit (NADMED, Helsinki, Finland) according to manufacturer's instructions. NAD serial standard dilutions were made from the concentrated NAD+ stock (NADMED). In parallel, total protein was measured with the bicinchoninic assay (BCA) assay kit (Thermo Fisher Scientific) and NAD^+ levels were normalized to μg protein.

Flow cytometry

Cell cycle analysis was performed by flow cytometry using the BD FACSCelesta™ (BD Biosciences, San Jose, CA, USA). Adherent cells (2×10^5) were harvested, washed twice with 1× PBS, fixed in 70% ethanol at 4 °C overnight and resuspended in a solution containing propidium iodide (PI) at a concentration of 50 μg/ml. Cell percentages in the different phases of the cell cycle (G0/G1, S and G2 + M) were measured using BD FACSDiva Software (BD Biosciences).

Apoptosis was detected by flow cytometric analysis of Annexin V–FITC versus PI staining (Invitrogen-eBioscience, San Diego, CA, USA) according to manufacturer's instructions. The annexin V positive/PI negative cells were considered in early apoptosis, Annexin V positive/PI positive populations in late apoptosis.

To measure reactive oxygen species (ROS) production, HeLa cells were treated with 10 nM FK866 for the indicated times, harvested, washed in PBS and stained for 30 min at 37 °C with 25 μM of dihydroethidium (DHE; Thermo Fisher Scientific) dissolved in DMEM without FBS. About 20000 events were acquired by using BD FACSCelesta™ flow cytometer, gated using forward scatter and side scatter to exclude cell debris and analyzed with BD FACSDiva Software.

Immunohistochemistry (IHC)

The formalin-fixed and paraffin-embedded tissue blocks were sectioned (2 μm) and subjected to deparaffinization, rehydration and antigen retrieval, at low or high pH as suggested by primary

antibody datasheets used, by PT Link (Dako Omnis-Agilent). Endogenous peroxidase was blocked for 10 min with 3% hydrogen peroxide in methanol (peroxidase blocking solution, Dako Omnis-Agilent) and, successively, non-specific antibody binding was blocked for 20 min with protein blocking buffer (Dako Omnis-Agilent). Tissue sections were immunostained for 1 h at RT with the following antibodies: anti-TRF2 rabbit polyclonal (Novus Biologicals, 1:500), anti-γH2AX rabbit monoclonal (AbCam, EP854(2)Y, 1:500), anti-Ki67 mouse monoclonal (Dako Omnis, MIB-1, ready to use), anti-8-OHdG mouse monoclonal (Santa Cruz, 15A3, 1:800), mAb anti-PARP1 (Mab 551025; BD Pharmingen, San Jose, CA, USA); mAb anti-NAMPT (E-3; Santa Cruz Biotechnology); mAb anti-NAPRT (B-8; Santa Cruz Biotechnology); mAb anti-SIRT1 (B-10; Santa Cruz Biotechnology). Finally, the tissue sections were covered for 30 min at RT with secondary antibody (EnVision™ FLEX Dako Omnis). The signal was developed by using DAB detection kit (Dako Omnis), then sections were counterstained with Harry's modified Hematoxylin. Finally, slides were dehydrated with increasing alcohol and xylene and mounted with Eukitt (Sigma Aldrich). Immunostaining results were recorded as percentage of positive cells or as staining intensity per percentage of positive cells (immunoreactive score - IRS).

IF-FISH on tissue sections

The formalin-fixed and paraffin-embedded tissue blocks were sectioned (2 μm) and subjected to deparaffinization and rehydration with decreasing concentrations of ethanol. For antigen retrieval, sections were boiled in citrate buffer pH 6.0 (ThermoFisher Scientific) in the microwave for 15 min and washed twice with distilled water. After dehydratation with alcohol series and air drying for about 5 min, tissues were covered with hybridization solution containing Cy3-labeled (CCCTAA)3 peptide nucleic acid (PNA) telomere probe (Panagene, Daejeon, South Korea), denatured at 80°C for 3 min and hybridized for 2 h at RT in a humidified chamber. After hybridization, slides were washed with FISH solution (70% formamide, 10 mM Tris pH 7.2, 0.1% BSA) and then dehydrated with ethanol series and air-dried. Immediately after the hybridization step, the IF protocol was performed. Sections

were blocked for 1 hour in a PBS solution containing 0.3% Triton X-100 and 1,5% BSA, and then incubated overnight with mAb anti-phospho-histone H2A.X (γH2AX, Ser139, clone JBW301; Millipore, dilution 1:300) at 4°C in a humid chamber. The next day, sections were incubated for 30 min at RT with Alexa Fluor-488 secondary antibody. Finally, sections were washed with PBS, dehydrated with alcohol series, and mounted with a drop of ProLong™ Antifade Mountant with DAPI (Invitrogen). Fluorescence signals were acquired at 63x magnification by using the Zeiss Laser Scanning Microscope 510 Meta (Zeiss, Germany). For Telomere-Induced Foci (TIFs) analysis, 90 γH2AX-positive nuclei on a single plane were scored.

Clinical patients' data set analysis

Normalized gene expression data of the Metabric breast cancer cohort (Curtis et al., 2012) and the clinical information were downloaded from cBioPortal (https://www.cbioportal.org) (Cerami et al., 2012; Gao et al., 2013).

A Fisher's exact test was used to evaluate differences of clinical variables between subgroup of patients with high or low expression of TERF2 and NAMPT genes. Disease-specific survival (DSS) was performed by Kaplan-Meier analysis and the log-rank test was used to assess differences between curves. High and low gene expression were defined considering z-scores higher or lower 0.5, respectively. A multivariate Cox proportional-hazards regression model was built to evaluate the effect of clinical variables on survival analysis. Hazard risk was adjusted for tumor size, lymph nodes status and stage. The analyses were completely conducted with Matlab R2022b.

References

Cerami, E., Gao, J., Dogrusoz, U., Gross, B. E., Sumer, S. O., Aksoy, B. A., Jacobsen, A., Byrne, C. J., Heuer, M. L., Larsson, E., Antipin, Y., Reva, B., Goldberg, A. P., Sander, C., & Schultz, N. (2012). The cBio cancer genomics portal: an open platform for exploring multidimensional cancer genomics data. *Cancer Discovery*, *2*(5), 401–404. https://doi.org/10.1158/2159-8290.CD-12-0095

Curtis, C., Shah, S. P., Chin, S. F., Turashvili, G., Rueda, O. M., Dunning, M. J., Speed, D., Lynch, A. G., Samarajiwa, S., Yuan, Y., Gräf, S., Ha, G., Haffari, G., Bashashati, A., Russell, R.,

McKinney, S., Aparicio, S., Brenton, J. D., Ellis, I., … Caldas, C. (2012). The genomic and transcriptomic architecture of 2,000 breast tumours reveals novel subgroups. *Nature 2012 486:7403*, *486*(7403), 346–352. https://doi.org/10.1038/nature10983

Gao, J., Aksoy, B. A., Dogrusoz, U., Dresdner, G., Gross, B., Sumer, S. O., Sun, Y., Jacobsen, A., Sinha, R., Larsson, E., Cerami, E., Sander, C., & Schultz, N. (2013). Integrative analysis of complex cancer genomics and clinical profiles using the cBioPortal. *Science Signaling*, *6*(269). https://doi.org/10.1126/scisignal.2004088

Perner, S., Brüderlein, S., Hasel, C., Waibel, I., Holdenried, A., Ciloglu, N., Chopurian, H., Vang Nielsen, K., Plesch, A., Högel, J., & Möller, P. (2003). Quantifying telomere lengths of human individual chromosome arms by centromere-calibrated fluorescence in situ hybridization and digital imaging. *The American Journal of Pathology*, *163*(5), 1751–1756. https://doi.org/10.1016/S0002-9440(10)63534-1

Salvati, E., Leonetti, C., Rizzo, A., Scarsella, M., Mottolese, M., Galati, R., Sperduti, I., Stevens, M. F. G., D'Incalci, M., Blasco, M., Chiorino, G., Bauwens, S., Horard, B., Gilson, E., Stoppacciaro, A., Zupi, G., & Biroccio, A. (2007). Telomere damage induced by the G-quadruplex ligand RHPS4 has an antitumor effect. *Journal of Clinical Investigation*, *117*(11). https://doi.org/10.1172/JCI32461

Surace, C., Berardinelli, F., Masotti, A., Roberti, M. C., Da Sacco, L., D'Elia, G., Sirleto, P., Digilio, M. C., Cusmai, R., Grotta, S., Petrocchi, S., Hachem, M. El, Pisaneschi, E., Ciocca, L., Russo, S., Lepri, F. R., Sgura, A., & Angioni, A. (2014). Telomere shortening and telomere position effect in mild ring 17 syndrome. *Epigenetics & Chromatin*, *7*(1). https://doi.org/10.1186/1756-8935-7-1
