# Supplementary material for: Drug repositioning strategy for the identification of novel telomere‐damaging agents: A role for NAMPT inhibitors
Source: Aging Cell. 2023 Oct 19;22(11):e13944. doi: 10.1111/acel.13944 (PMC10652301; doi:10.1111/acel.13944)
Supplement: Supplementary file 2 — Figure S1–S11. [file ACEL-22-e13944-s005.pdf]

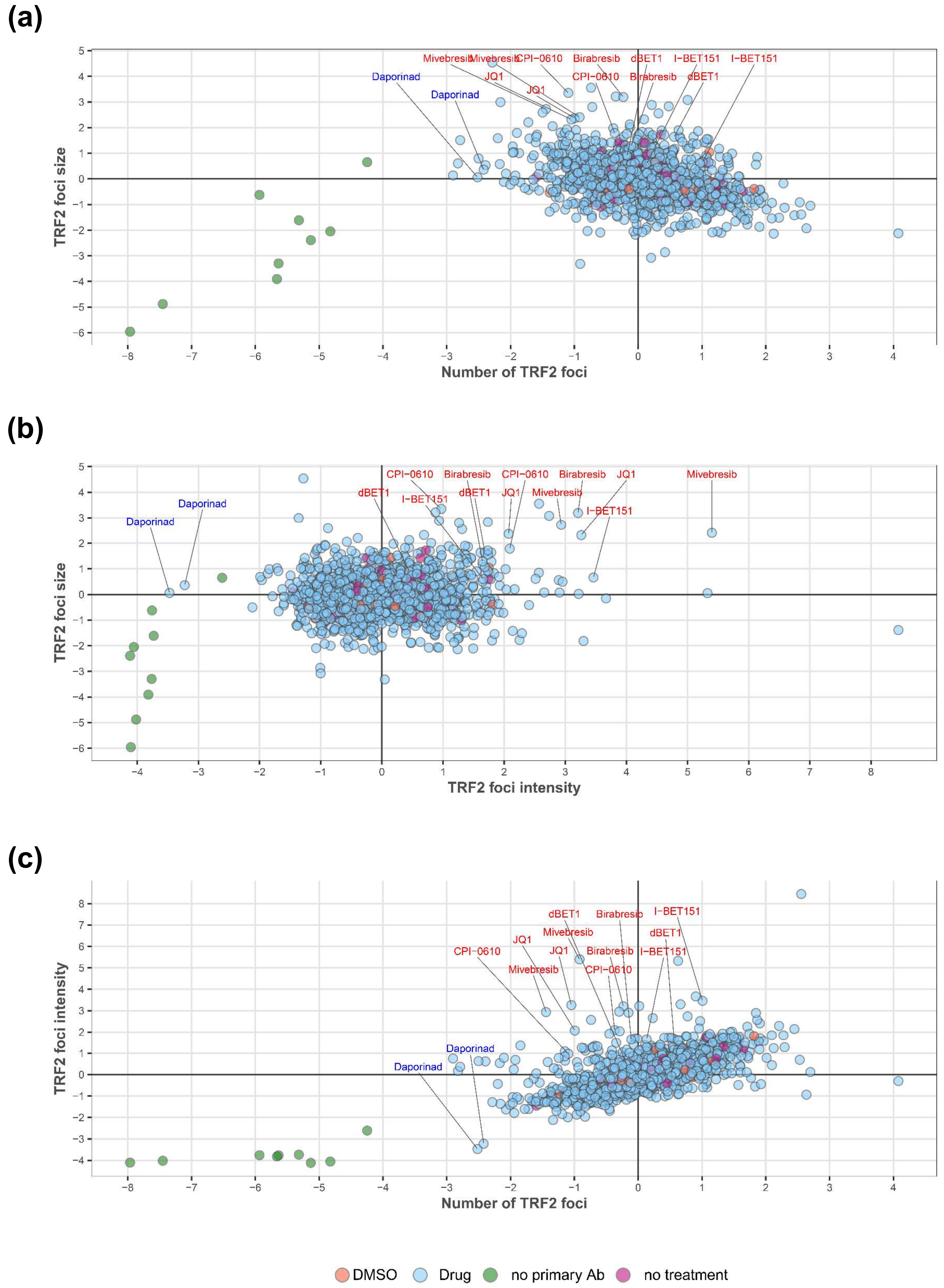

Fig. S1. High-throughput drug screening analysis.

Scatterplots reporting the z-score values calculated for the indicated parameters of TRF2 foci signals referred to each tested drug. "No treatment" and "DMSO only" wells were used as controls which drug-treated wells have been normalized to. Drugs with a z-score below -1 were considered negative regulators of TRF2 foci, while those with a z-score above 1 were considered positively affecting TRF2 foci.

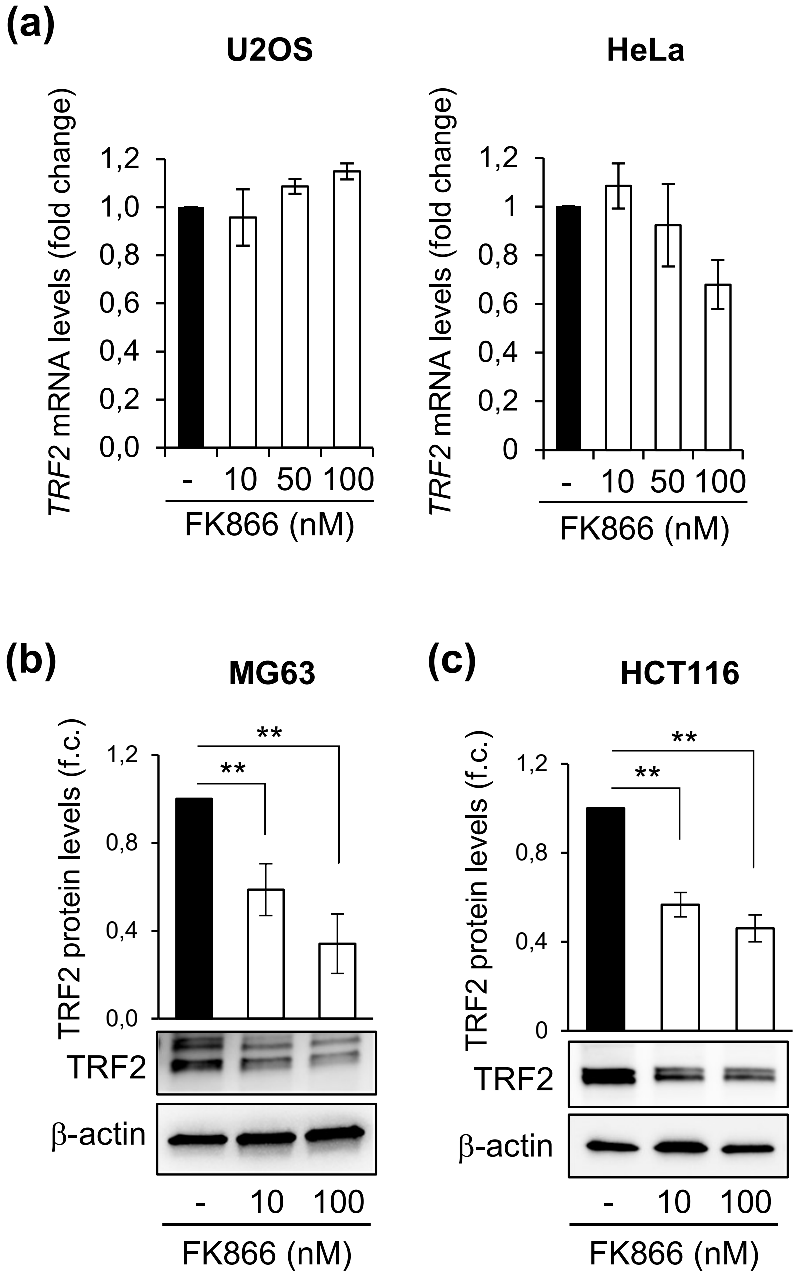

Fig. S2. Evaluation of TRF2 levels in different *in vitro* tumor models. (a) qPCR analysis of TRF2 mRNA in U2OS (left panel) and HeLa (right panel) cells treated with the indicated doses of FK866 for 24 h or DMSO as control. All histograms show the TRF2 expression levels expressed as fold changes of FK866- versus DMSO-treated samples, after β-actin normalization. The mean values of three independent experiments ± SD are reported. **(b - c)** WB analysis of TRF2 protein levels in human osteosarcoma MG63 and in colon carcinoma HCT116 cells treated for 48 h with the indicated doses of FK866 or DMSO as control. β-actin was used as loading control. The mean values of three independent experiments ± SD are reported. Unpaired two-tailed t-test. (** $p < 0.01$)

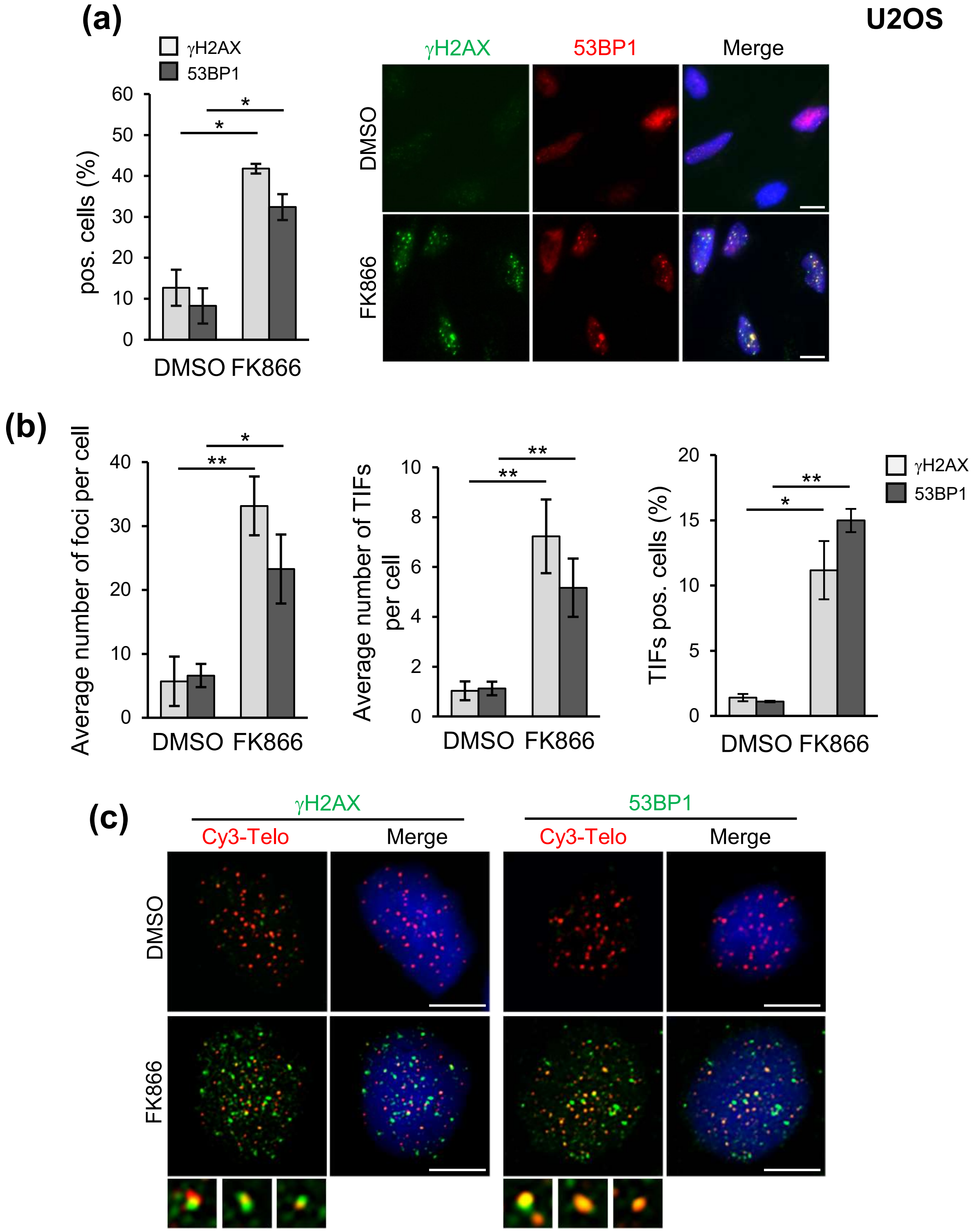

Fig. S3. NAMPT-inhibitor FK866 induces telomere damage. U2OS cells were treated with DMSO as control or 10 nM FK866 for 48 h and then processed for IF and FISH analysis. **(a)** Histograms report the percentage of γH2AX and 53BP1 foci-positive cells quantified in IF experiments (left panel). The average of three independent experiments ± SD is shown. Representative images of IF (right panel). Scale bars, 10 µm. **(b)** The average number of foci per cell (left panel), the average number of Cy3-Telo PNA probe/gH2AX or 53BP1 co-localizations (TIFs) per cell (middle panel) and the percentage of cells displaying ≥4 gH2AX- or 53BP1-TIFs (right panel) were scored in three independent experiments of IF-FISH (n ≥ 90 nuclei). Bars indicate means ± SD. Unpaired two-tailed t-test was used to calculate *p*-values. **(c)** Representative images of Telo-FISH are shown with the enlargements of some colocalizing foci. Scale bars, 10 µm. Unpaired two-tailed t-test. (**p* < 0.05; ***p* < 0.01).

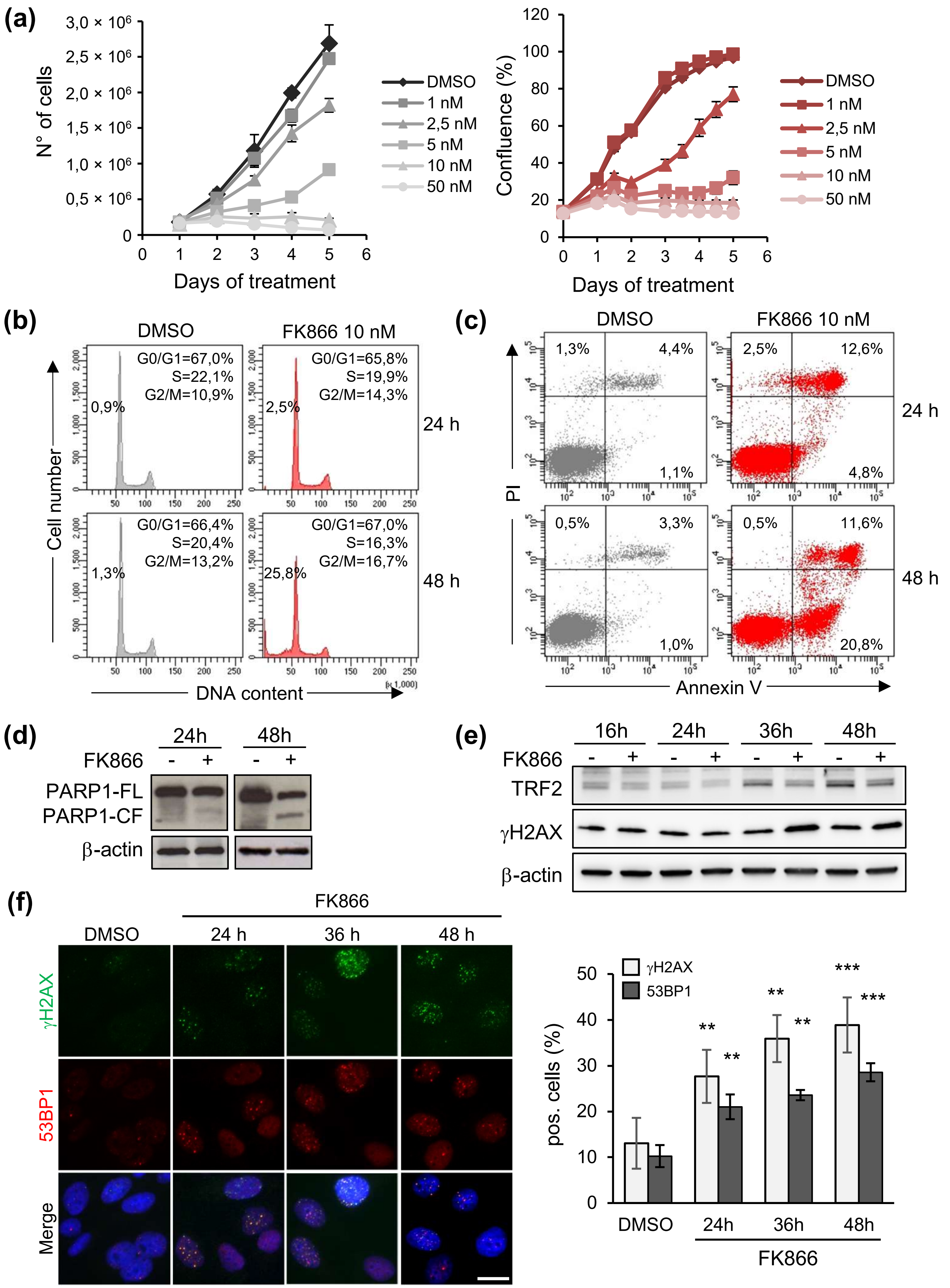
(a)
N° of cells
3,0 × 10⁶
2,5 × 10⁶
2,0 × 10⁶
1,5 × 10⁶
1,0 × 10⁶
0,5 × 10⁶
Days of treatment
DMSO
1 nM
2,5 nM
5 nM
10 nM
50 nM
Confluence (%)
(b)
DMSO
FK866 10 nM
G0/G1=67,0% S=22,1% G2/M=10,9%
0,9%
G0/G1=65,8% S=19,9% G2/M=14,3%
2,5%
24 h
G0/G1=66,4% S=20,4% G2/M=13,2%
1,3%
G0/G1=67,0% S=16,3% G2/M=16,7%
25,8%
48 h
Cell number
DNA content
(c)
DMSO
FK866 10 nM
1,3% 4,4% 1,1%
2,5% 12,6% 4,8%
0,5% 3,3% 1,0%
0,5% 11,6% 20,8%
PI
Annexin V
(d)
24h 48h
FK866 - + - +
PARP1-FL
PARP1-CF
β-actin
(e)
16h 24h 36h 48h
FK866 - + - + - + - +
TRF2
γH2AX
β-actin
(f)
FK866
DMSO 24 h 36 h 48 h
γH2AX
53BP1
Merge
pos. cells (%)
γH2AX
53BP1
** ** ** ** *** ***
DMSO 24h 36h 48h
FK866

Fig. S4. NAMPT-inhibitor FK866 induces cell death. (a) HeLa cells were treated with DMSO as control or FK866 at the indicated doses. Starting from the day of treatment, cell proliferation was evaluated once a day (left panel) and cell confluence was monitored by Incucyte every 12 h up to 5 days. The means of three independent experiments ± SD are reported. **(b)** Cell cycle progression analysis by Propidium Iodide (PI) staining and **(c)** cell death analysis by Annexin V assay were performed in HeLa cells treated with DMSO as control or 10 nM FK866. In parallel, the same samples were processed by WB **(d)** for the evaluation of cleaved (CF) or full-length (FL) form of PARP1 protein. β-actin was used as loading control. **(e)** Hela cells were treated with DMSO as control or 10 nM FK866 and the protein levels of TRF2 and γH2AX were analyzed at the indicated time-points by WB. β-actin was used as loading control. Representative immunoblots of three independent experiments are shown. **(f)** In parallel, the same samples of (e) were processed for IF analysis of γH2AX and 53BP1 staining. Representative images of IF are shown in the left panel. Scale bars, 10 μm. Histograms report the mean percentages of γH2AX and 53BP1 foci-positive cells (right panel) ± SD quantified from three independent experiments ($n \geq 90$ nuclei). Unpaired two-tailed t-test. (**$p < 0.01$;***$p < 0.001$).

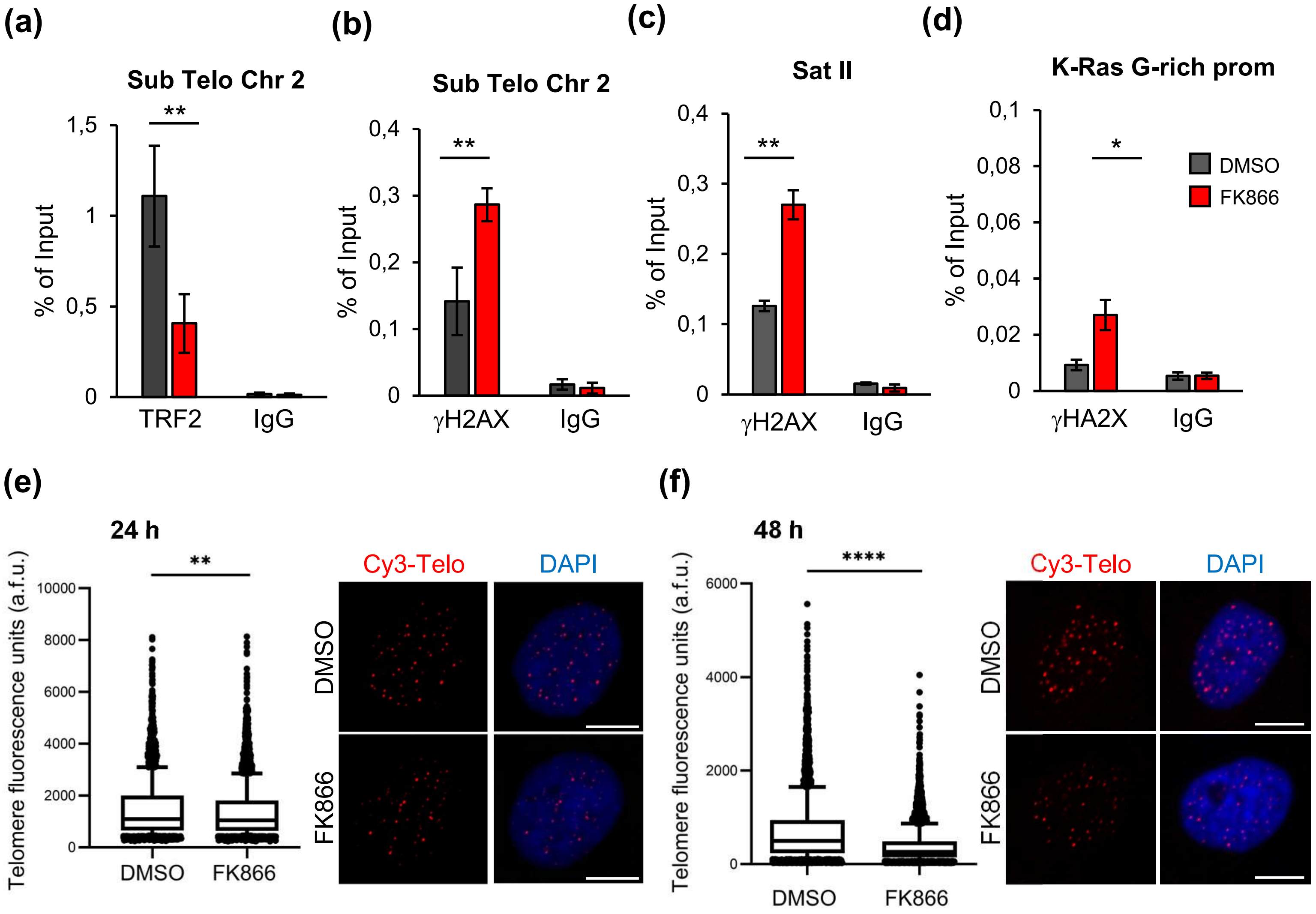

Fig. S5. NAMPT inhibitor FK866 induces telomere dysfunction. HeLa cells were exposed to DMSO as control or 10 nM FK866 for 48h and then processed for ChIP and FISH assays. Histograms report the amount of TRF2 binding to sub-telomeric chromatin **(a)** and γH2AX enrichment at sub-telomere **(b)**, Satellite II regions **(c)** and k-Ras G-rich promoter **(d)** quantified by qPCR-ChIP analysis after normalization relative input chromatin. The mean of three independent experiments ± SD are shown. Unpaired two-tailed t-test was used to calculate *p*-values. Fluorescence intensity quantification of telomeric signals in HeLa cells treated with DMSO as control or 10 nM FK866 for 24 h **(e)** or 48 h **(f)** and processed for Telo-FISH assay (n ≥ 90 nuclei). Box plots in the left panels: middle line represents the median value of arbitrary telomere fluorescence units (a.f.u.), boxes extend from the 25th to 75th percentiles and the whiskers mark the 10th and 90th percentiles. Statistical significance from three independent experiments was calculated by two-tailed Mann–Whitney test. Representative images of Telo-FISH are shown in the respective right panels. (*$p < 0.05$; **$p < 0.01$; ****$p < 0.0001$).

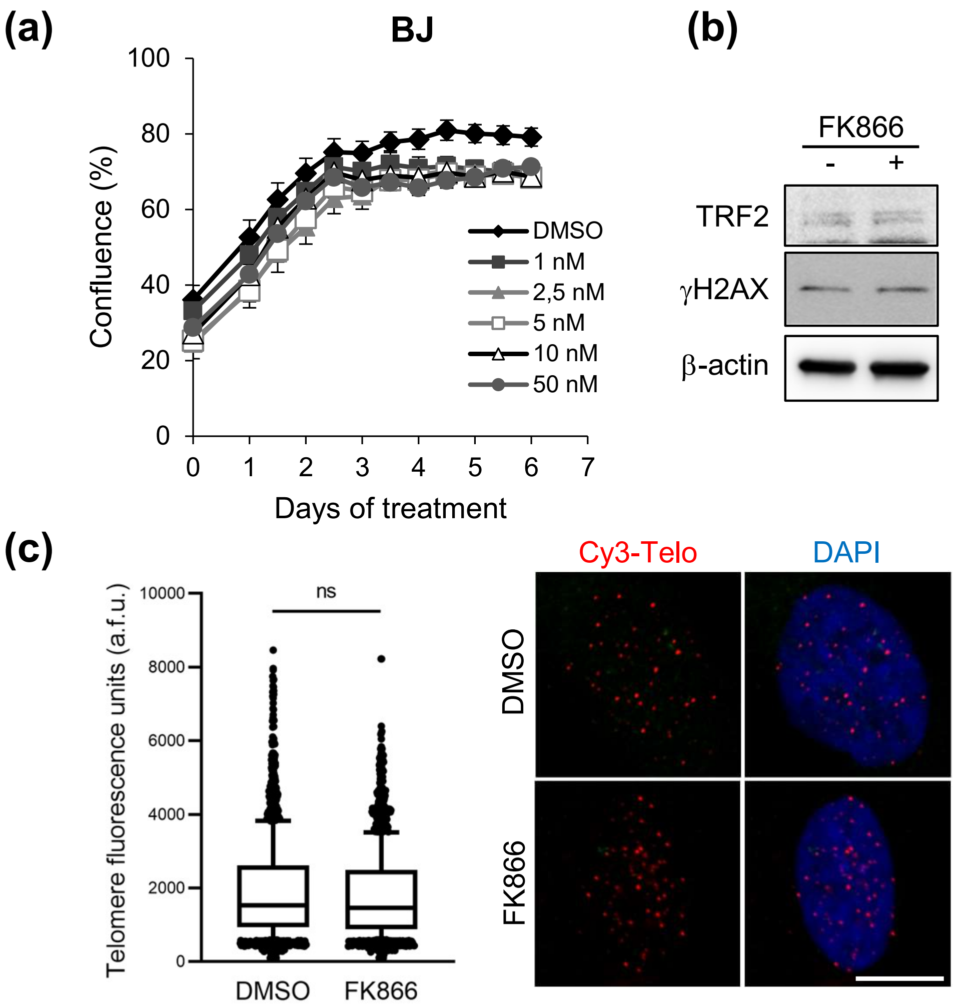

Fig. S6. NAMPT-inhibitor FK866 does not affect telomere length of normal cells. Human normal BJ fibroblasts were treated with DMSO as control or FK866 at the indicated doses. Starting from the day of treatment cell confluence was monitored by Incucyte every 12 h up to 6 days. The mean of three independent experiments ± SD is reported. **(b)** BJ cells were exposed to DMSO as control or 10 nM FK866 for 48h and then processed for WB analysis of TRF2 and γH2AX protein levels. β-actin was used as loading control. Representative images of three independent experiments are shown. **(c)** Fluorescence intensity of each telomeric signal (n ≥ 90 nuclei) was quantified in experiments of telomere FISH in BJ cells treated as in (b). Left panel: in the box plot diagrams the middle line represents median value of arbitrary telomere fluorescence units (a.f.u.), boxes extend from the 25th to 75th percentiles and the whiskers mark the 10th and 90th percentiles. The statistical significance from three independent experiments was evaluated by two-tailed Mann–Whitney test. Representative images of analyzed interphasic nuclei are shown on the right panel. Scale bars, 10 μm. ns, not significant.

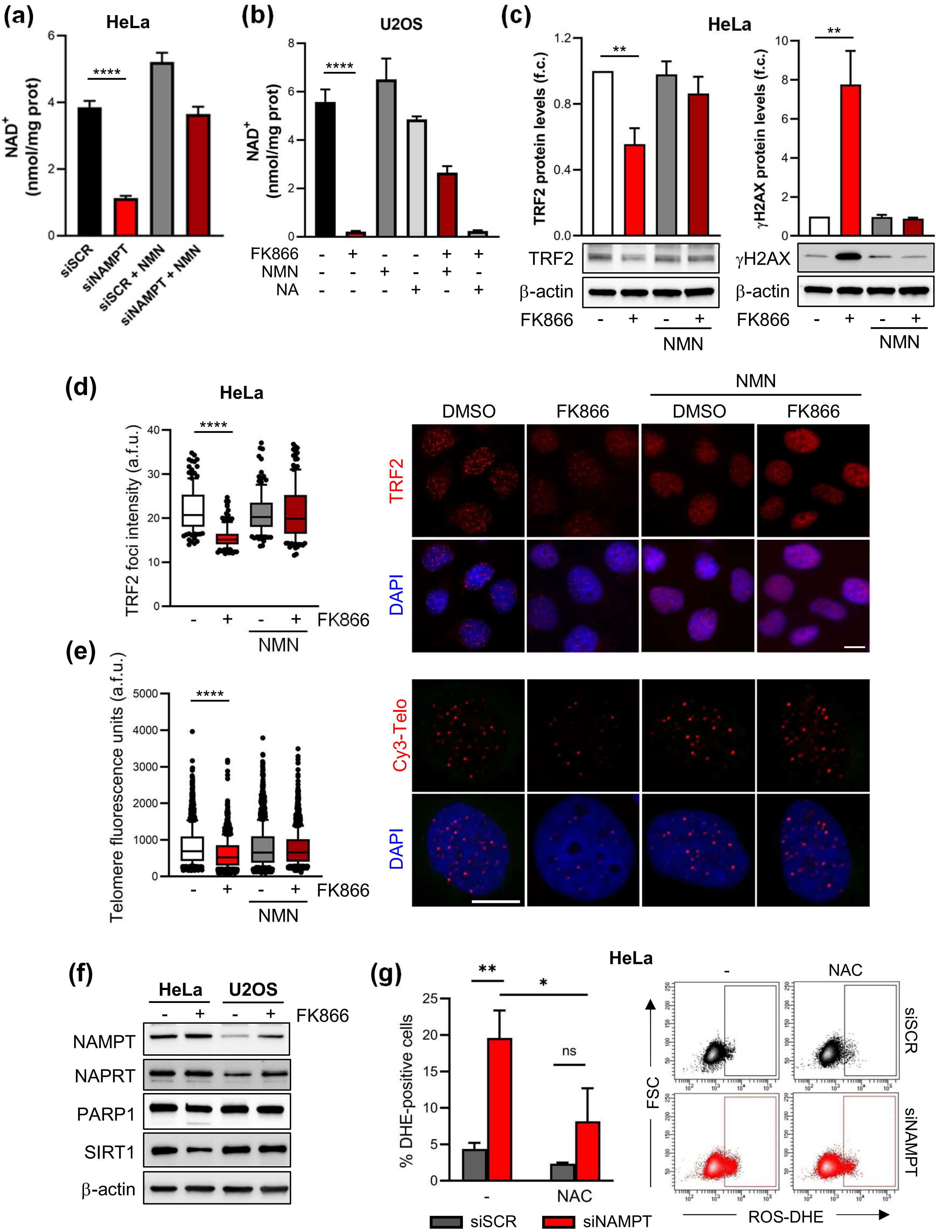
(a)
HeLa
NAD+ (nmol/mg prot)
****
siSCR
siNAMPT
siSCR + NMN
siNAMPT + NMN
(b)
U2OS
NAD+ (nmol/mg prot)
****
FK866
NMN
NA
(c)
HeLa
TRF2 protein levels (f.c.)
γH2AX protein levels (f.c.)
**
TRF2
β-actin
γH2AX
FK866
NMN
(d)
HeLa
TRF2 foci intensity (a.f.u.)
****
DMSO
FK866
TRF2
DAPI
(e)
Telomere fluorescence units (a.f.u.)
Cy3-Telo
(f)
HeLa
U2OS
NAMPT
NAPRT
PARP1
SIRT1
(g)
% DHE-positive cells
ns
NAC
siSCR
siNAMPT
FSC
ROS-DHE

Figure S7. NAD-depletion and ROS production in response to NAMPT inhibition. (a) HeLa cells transiently silenced for NAMPT (siNAMPT) or exposed to scramble siRNA (siSCR) were processed after 48 h of transfection for the evaluation of NAD^+ content. The same analysis was performed upon co-treatment with 250 μM NMN. **(b)** NAD+ content in U2OS cells treated for 48 h with 10 nM FK866 or DMSO as control, alone or in combination with 250 μM NMN or 25 μM NA. The mean of three independent experiments ± SD is shown. **(c)** WB analysis of TRF2 and γH2AX protein levels in HeLa cells treated for 48 h with 10 nM FK866 or DMSO as control, alone or in combination with 250 μM NMN. β-actin was used as loading control. The mean values of three independent experiments ± SD are reported. Unpaired two-tailed t-test (**$p < 0.01$). Hela cells treated as in **(c)** were processed for TRF2 IF analysis **(d)** and telomere length evaluation by Telo-FISH assay **(e)**. Box plots in the left panels: middle line represents the median value of arbitrary fluorescence units (a.f.u.), boxes extend from the 25th to 75th percentiles and the whiskers mark the 10th and 90th percentiles (two-tailed Mann–Whitney test; ****$p < 0.0001$). Representative images of three independent experiments of IF and Telo-FISH are shown in the respective right panels. **(f)** WB analysis of analysis of the indicated proteins in HeLa and U2OS cells treated for 48 h with 10 nM FK866 or DMSO as control. β-actin was used as loading control. Representative images of three independent experiments are shown. **(g)** FACS analysis of ROS-positive cells in siSCR or siNAMPT HeLa cells in presence or absence of the antioxidant NAC (5 mM). The analysis was performed after 48 h of NAC exposure, 72 h post-transfection. In the left panel the average of three independent experiments with SD is reported. The FACS analysis of a representative experiment is shown in the right panel. (Unpaired two-tailed t-test; *$p < 0.05$; **$p < 0.01$; ****$p < 0.0001$).

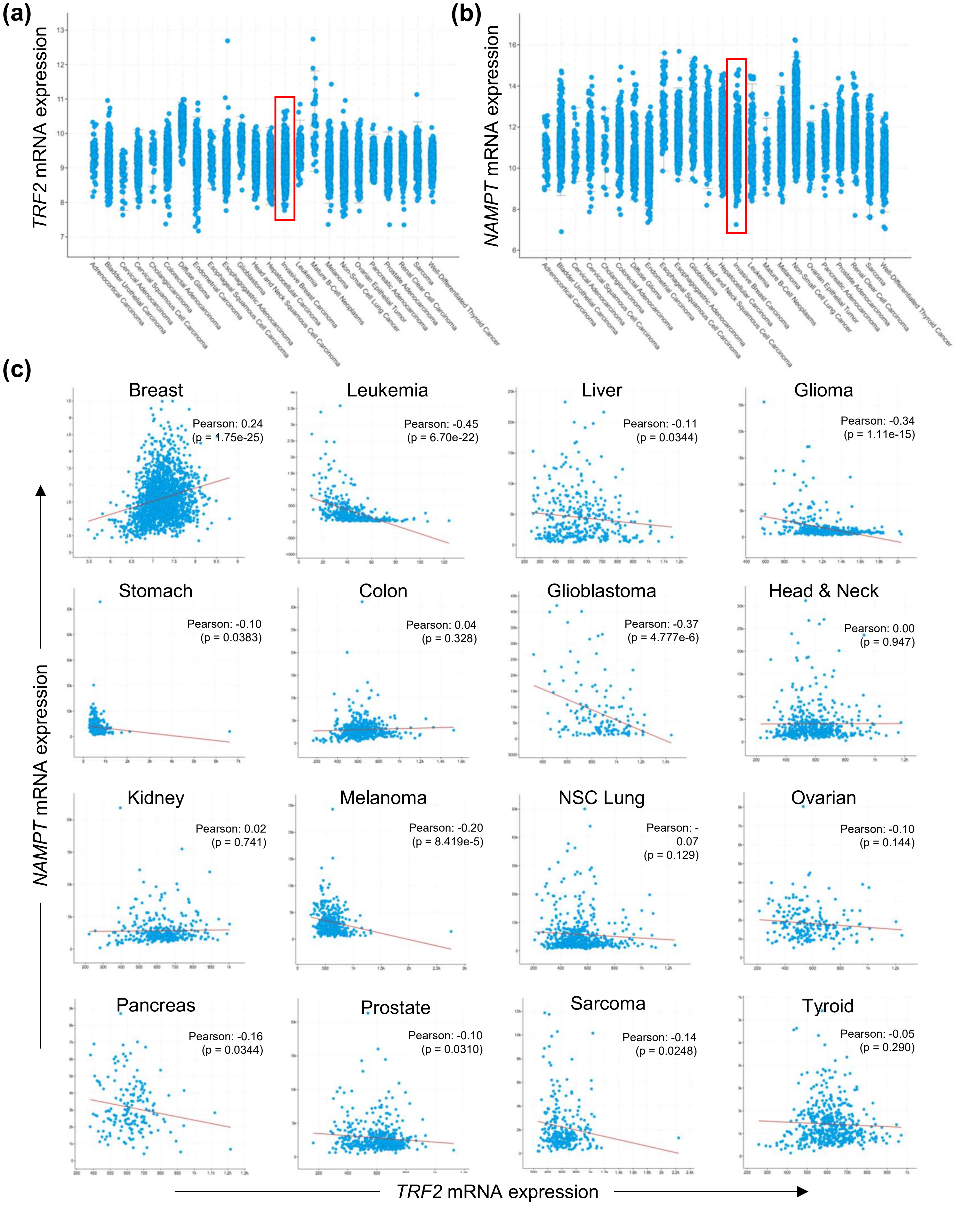

Fig. S8. Correlation analysis between *TRF2* and *NAMPT* expression in human malignancies. RNAseq analysis of *TRF2* **(a)** and *NAMPT* **(b)** mRNA expression in 24 types of malignancies compared with relative normal tissues. **(c)** Pearson's correlation analysis between *TRF2* and *NAMPT* mRNA expression in a selected panel of cancer histotypes. Pearson's correlation and *p*-values are shown inside each graph. The reported results were obtained interrogating publicly available cancer genomic datasets from the cBioPortal platform.

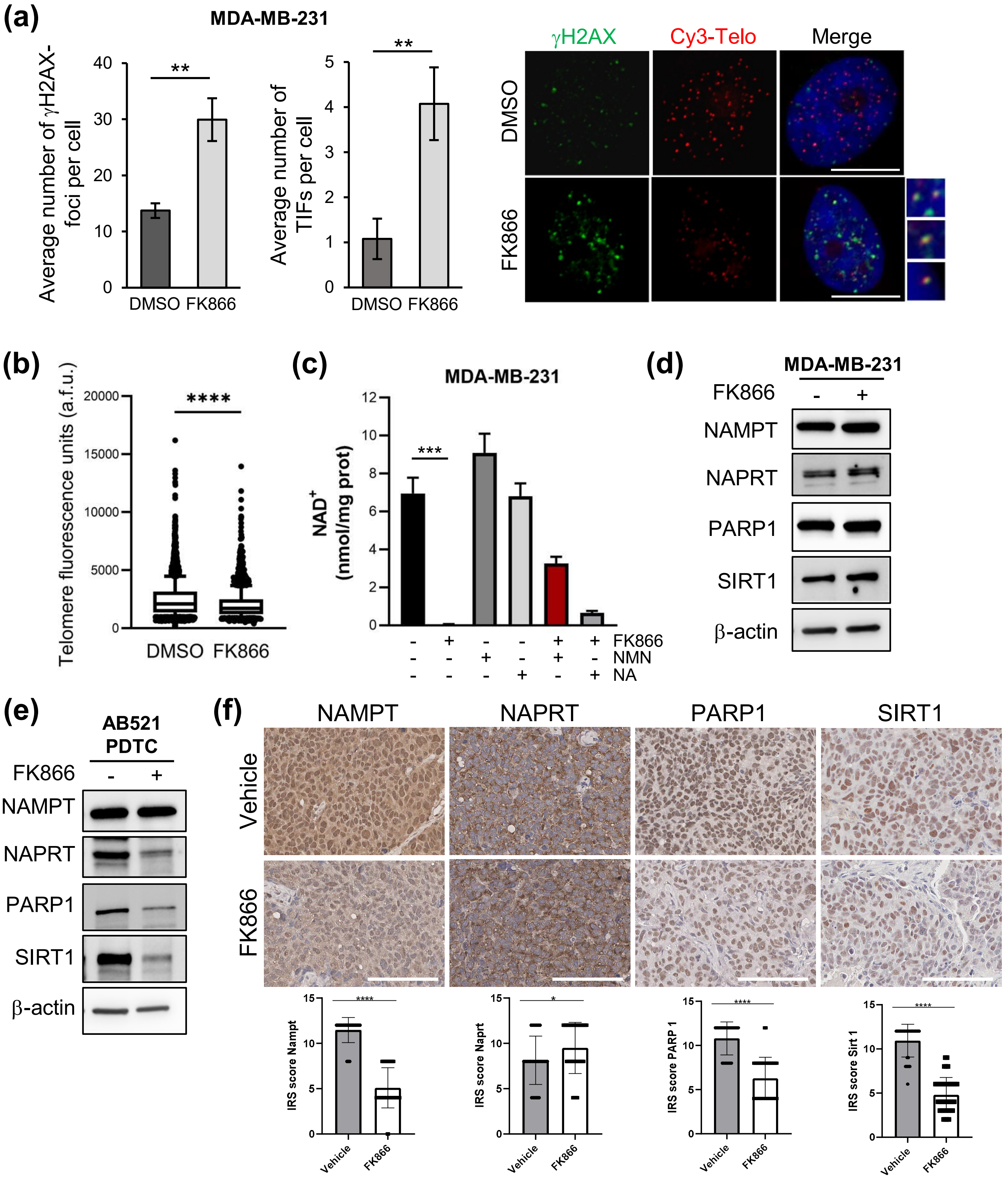

(a)
MDA-MB-231
Average number of γH2AX-foci per cell
Average number of TIFs per cell
DMSO FK866
γH2AX
Cy3-Telo
Merge
DMSO
FK866
(b)
Telomere fluorescence units (a.f.u.)
DMSO FK866
(c)
MDA-MB-231
NAD+ (nmol/mg prot)
FK866
NMN
NA
(d)
MDA-MB-231
FK866
NAMPT
NAPRT
PARP1
SIRT1
β-actin
(e)
AB521 PDTC
FK866
NAMPT
NAPRT
PARP1
SIRT1
β-actin
(f)
NAMPT
NAPRT
PARP1
SIRT1
Vehicle
FK866
IRS score Nampt
IRS score Naprt
IRS score PARP 1
IRS score Sirt 1
Vehicle
FK866

Fig. S9. Effects of FK866 treatment on telomeres and NAD-pathway in triple negative breast cancer (TNBC) models. (a-d) Human TNBC MDA-MB-231 cells were treated for 48 h with 10 nM FK866 or DMSO as control and then processed for telomere integrity and NAD-pathway. **(a)** The average number of γH2AX-foci (left panel) and γH2AX-TIFs (middle panel) per cell was scored in three independent experiments of telomere IF-FISH (n ≥ 90 nuclei). Bars indicate means ± SD. Unpaired two-tailed t-test was used to calculate *p*-values. Representative images of Telo-FISH are shown with the enlargements of some colocalizing foci (right panel). Scale bars, 10 μm. **(b)** Fluorescence intensity quantification of telomeric signals (n ≥ 90 nuclei). Box plots in the left panels: middle line represents the median value of arbitrary telomere fluorescence units (a.f.u.), boxes extend from the 25th to 75th percentiles and the whiskers mark the 10th and 90th percentiles. Statistical significance from three independent experiments was calculated by two-tailed Mann–Whitney test. **(c)** NAD+ content in MDA-MB-231 cells treated for 48 h with 10 nM FK866 or DMSO as control, alone or in combination with 250 μM NMN or 25 μM NA. The mean of three independent experiments ± SD is shown. **(d)** WB analysis of analysis of the indicated proteins in MDA-MB-231 cells treated for 48 h with 10 nM FK866 or DMSO as control. **(e)** WB analysis of analysis of the indicated proteins in lysates from AB521 PDTCs treated for 72 h with 10 nM FK866 or DMSO as control. (d-e) β-actin was used as loading control. **(f)** Three mice per group of untreated and FK866-exposed mice as described in M&M section were sacrificed at the end of the treatment and tumor samples were excised for *ex vivo* IHC analyses. In the upper panel representative images of IHC staining for the indicated markers are reported. Scale bar: 100 μm. The histograms in the bottom panel show the quantification of the IHC assays expressed as immunoreactivity score (IRS). Bars indicate means ± SD. Unpaired two-tailed t-test; **p* < 0.05; ***p* < 0.01; ****p*<0.001; *****p*<0.0001.

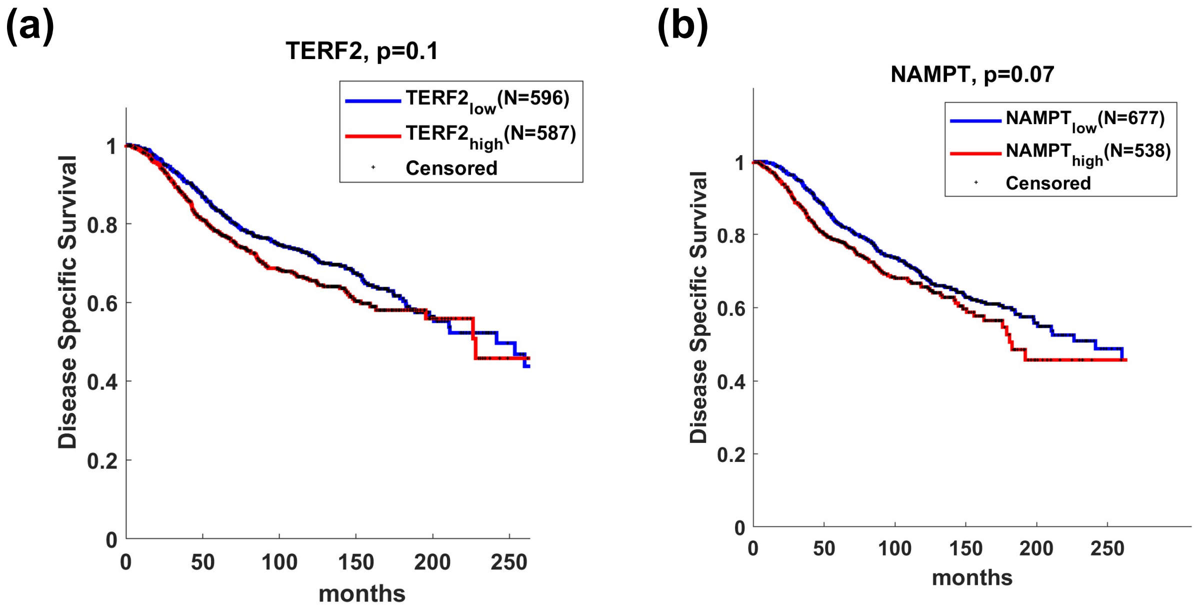

Fig. S10. Clinical outcome of BC patients stratified for *TRF2* or *NAMPT* mRNA expression levels. Disease Specific Survival evaluated by Kaplan–Meier curves on a cohort of 1215 BC patients from the Metabric dataset. Survival curves of patients, stratified on the basis of *TRF2* **(a)** or *NAMPT* **(b)** mRNA expression levels, are reported. The log-rank test was used to assess differences between curves. High and low gene expression were defined considering z-scores higher or lower 0.5, respectively.

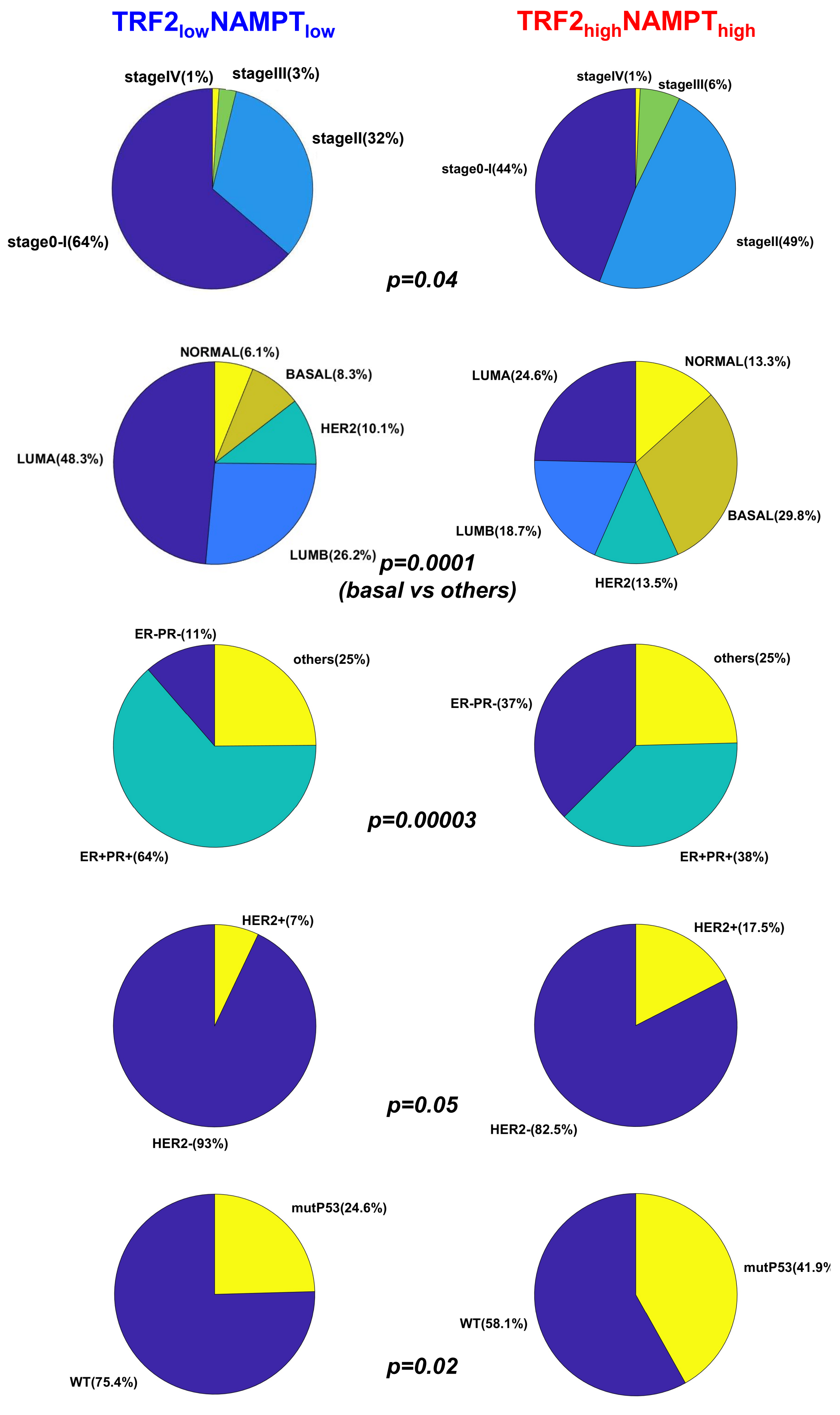

Fig. S11. Clinical stratification of BC patients according to *TRF2* and *NAMPT* mRNA expression. Pie chart representing the frequency of the indicated clinical variables between subgroups of BC patients from the Metabric dataset, characterized by low or high expression of both *TRF2* and *NAMPT* genes. A Fisher's exact test was used to evaluate differences of clinical variables between the subgroups of patients.
