# Supplementary material for: Drug repositioning strategy for the identification of novel telomere‐damaging agents: A role for NAMPT inhibitors
Source: Aging Cell. 2023 Oct 19;22(11):e13944. doi: 10.1111/acel.13944 (PMC10652301; doi:10.1111/acel.13944)
Supplement: Supplementary file 3 — Table S1. [file ACEL-22-e13944-s003.pdf]

Table S1 - Details of screening results

Drug name & concentration	Number of TRF2 foci	TRF2 foci intensity	TRF2 foci size	Mechanism/Targets
1 - Daporinad_100nM	-2.42	-3.22	0.36	NAMPT inhibitor
2 - Ensartinib_100nM	0.2	-1.00	-3.08	ALK inhibitor
3 - Decernotinib_1000nM	0.42	-1.01	-2.86	JAK3 inhibitor
4 - Afatinib_100nM	-2.01	-1.41	-0.23	EGFR inhibitor
5 - Cerdulatinib_1000nM	-1.33	-2.12	-0.50	JAK, SYK inhibitor
6 - Dexamethasone_1000nM	-0.01	-0.84	-2.00	Glucocorticoid, immunomodulatory agent
7 - Fludarabine_1000nM	-0.59	-1.08	-2.09	Antimetabolite; Purine analog
8 - Pemetrexed_10000nM	-0.79	0.00	-2.03	Dihydrofolate reductase inhibitor
9 - Raltitrexed_100nM	0.07	0.77	-2.08	DHFR/GARFT/thymidylate synthase inhibitor
10 - Ruxolitinib_1000nM	-0.07	-0.55	-2.05	JAK1&2 inhibitor
11 - Tretinoin_1000nM	-1.94	-1.79	-0.07	Retinoic acid receptor agonist
12 - Trifluridine_1000nM	-0.04	-0.13	-2.18	Antimetabolite; Nucleoside analog
13 - CPI-613_1000nM	1.66	1.35	-1.25	Pyruvate dehydrogenase, alpha-ketoglutarate dehydrogenase inhibitor
14 - Dasatinib_100nM	-1.34	-0.57	2.06	Abl, Src, Kit, EphR... Inhibitor
15 - Daunorubicin_100nM	0.91	1.24	-1.73	Topoisomerase II inhibitor
16 - dBET1_1000nM	-0.17	0.45	0.90	BET-targeting PROTAC
17 - Gemcitabine_100nM	0.29	0.63	-1.86	Antimetabolite; Nucleoside analog
18 - SCH772984_100nM	1.27	1.37	-0.24	ERK1 & 2 inhibitor
19 - A-1155463_1000nM	1.33	1.71	-0.13	BCL-XL inhibitor
20 - Carboplatin_10000nM	0.75	1.80	-1.80	Platinum-based antineoplastic agent
21 - Doxorubicin_100nM	1.24	0.91	-2.05	Topoisomerase II inhibitor
22 - Floxuridine_1000nM	0.21	1.36	-1.49	Antimetabolite; Analog of 5-fluorouracil
23 - Topotecan_1000nM	0.36	1.26	-1.69	Topoisomerase I inhibitor. Camptothecin analog
24 - AT9283_100nM	2.64	-0.94	-1.93	Aurora A & B, Jak2, Flt, Abl inhibitor
25 - Amsacrine_1000nM	1.30	1.49	-2.11	DNA intercalation, Topo II inhibitor
26 - AR-42_1000nM	-0.30	2.95	0.50	HDAC inhibitor
27 - Epirubicin_100nM	1.16	0.93	-2.08	Topoisomerase II inhibitor
28 - GSK269962_1000nM	-0.02	0.98	1.53	ROCK1 and ROCK2 inhibitor
29 - Marimastat_1000nM	0.52	1.73	0.92	MMP-9, MMP-1, MMP-2, MMP-14, MMP-7 inhibitor
30 - Aldoxorubicin_100nM	0.66	3.30	-1.82	Topoisomerase II inhibitor, Albumin binding
31 - EPZ031686_1000nM	0.32	1.31	2.55	SMYD3 inhibitor
32 - RGFP966_1000nM	0.95	2.17	0.21	HDAC3 inhibitor
33 - Tozasertib_1000nM	4.08	-0.30	-2.12	pan-Aurora inhibitor
34 - CPI-0610_1000nM	-1.09	0.97	3.35	BET family inhibitor
35 - I-BET151_1000nM	1.01	3.46	0.66	BET family inhibitor
36 - Birabresib_1000nM	-0.23	3.21	3.19	BET family inhibitor
37 - JQ1_1000nM	-1.05	3.26	2.32	BET family inhibitor
38 - Mivebresib_1000nM	-1.44	2.93	2.72	BET family inhibitor
39 - OSU-03012_2500nM	0.62	5.33	0.05	PDPK1 inhibitor
40 - ML390_5000nM	2.56	8.45	-1.39	DHODH inhibitor
