# Supplementary material for: Drug repositioning strategy for the identification of novel telomere‐damaging agents: A role for NAMPT inhibitors
Source: Aging Cell. 2023 Oct 19;22(11):e13944. doi: 10.1111/acel.13944 (PMC10652301; doi:10.1111/acel.13944)
Supplement: Supplementary file 4 — Table S2. [file ACEL-22-e13944-s002.pdf]

Table S2 - Details of drugs used for the screening

Preferred name	Mechanism/Targets	Class explained	High phase/Approval status	Supplier Ref	Supplier	Final conc (nM)
8-amino-adenosine	Nucleoside analog, RNA synthesis inhibitor	A. Conv. Chemo	Probe		Santa Cruz Biotechnology	5000
8-amino-adenosine	Nucleoside analog, RNA synthesis inhibitor	A. Conv. Chemo	Probe		Santa Cruz Biotechnology	500
8-chloro-adenosine	Nucleoside analog, RNA synthesis inhibitor	A. Conv. Chemo	Investigational (Ph 2)		Santa Cruz Biotechnology	5000
8-chloro-adenosine	Nucleoside analog, RNA synthesis inhibitor	A. Conv. Chemo	Investigational (Ph 2)		Santa Cruz Biotechnology	500
ABT-751	Mitotic inhibitor. Colchicine site binding microtubule depolymerizer.	A. Conv. Chemo	Investigational (Ph 2)		Selleck	1000
ABT-751	Mitotic inhibitor. Colchicine site binding microtubule depolymerizer.	A. Conv. Chemo	Investigational (Ph 2)		Selleck	100
Aldoxorubicin	Topoisomerase II inhibitor, Albumin binding	A. Conv. Chemo	Investigational (Ph 3)		Medchem Express	100
Aldoxorubicin	Topoisomerase II inhibitor, Albumin binding	A. Conv. Chemo	Investigational (Ph 3)		Medchem Express	10
Allopurinol	Xanthine oxidase inhibitor	A. Conv. Chemo	Approved	HY-B0219	Medchem Express	1000
Allopurinol	Xanthine oxidase inhibitor	A. Conv. Chemo	Approved	HY-B0219	Medchem Express	100
Amsacrine	DNA intercalation, Topo II inhibitor	A. Conv. Chemo	Approved	sc-214540	Santa Cruz Biotechnology	1000
Amsacrine	DNA intercalation, Topo II inhibitor	A. Conv. Chemo	Approved	sc-214540	Santa Cruz Biotechnology	100
Auranofin	Antirheumatic agent	A. Conv. Chemo	Approved	A6733	Sigma-Aldrich	250
Auranofin	Antirheumatic agent	A. Conv. Chemo	Approved	A6733	Sigma-Aldrich	25
Bleomycin	Glycopeptide antibiotic; causes DNA breaks	A. Conv. Chemo	Approved	S1214	Selleck	1000
Bleomycin	Glycopeptide antibiotic; causes DNA breaks	A. Conv. Chemo	Approved	S1214	Selleck	100
Cabazitaxel	Taxane microtubule stabilizer, antimitotic	A. Conv. Chemo	Approved		Medchem Express	100
Cabazitaxel	Taxane microtubule stabilizer, antimitotic	A. Conv. Chemo	Approved	HY-15459	Medchem Express	10
Capecitabine	5-FU prodrug	A. Conv. Chemo	Approved	C-2799	LC Laboratories	1000
Capecitabine	5-FU prodrug	A. Conv. Chemo	Approved	C-2799	LC Laboratories	100
Carboplatin	Platinum-based antineoplastic agent	A. Conv. Chemo	Approved	S1215-3	Selleck	10000
Carboplatin	Platinum-based antineoplastic agent	A. Conv. Chemo	Approved	S1215-3	Selleck	1000
Chloroquine	Antimalaria agent; chemo/radio sensitizer	A. Conv. Chemo	Approved	C6628	Sigma-Aldrich	10000
Chloroquine	Antimalaria agent; chemo/radio sensitizer	A. Conv. Chemo	Approved	C6628	Sigma-Aldrich	1000
Cisplatin	Platinum-based antineoplastic agent	A. Conv. Chemo	Approved	S1166-4	Selleck	10000
Cisplatin	Platinum-based antineoplastic agent	A. Conv. Chemo	Approved	S1166-4	Selleck	1000
Cladribine	Antimetabolite; Purine analog	A. Conv. Chemo	Approved	HY-13599	Medchem Express	100
Cladribine	Antimetabolite; Purine analog	A. Conv. Chemo	Approved	HY-13599	Medchem Express	10
Clofarabine	Antimetabolite; Purine analog	A. Conv. Chemo	Approved	2600	Tocris Biosciences	1000
Clofarabine	Antimetabolite; Purine analog	A. Conv. Chemo	Approved	2600	Tocris Biosciences	100
Cytarabine	Antimetabolite, interferes with DNA synthesis	A. Conv. Chemo	Approved	HY-13605	Medchem Express	1000
Cytarabine	Antimetabolite, interferes with DNA synthesis	A. Conv. Chemo	Approved	HY-13605	Medchem Express	100
Cytarabine/Idarubicin	Std. Induction therapy combination	A. Conv. Chemo	Approved		Medchem Express/Sigma-Aldric	500/50
Cytarabine/Idarubicin	Std. Induction therapy combination	A. Conv. Chemo	Approved		Medchem Express/Sigma-Aldric	50/5
Dactinomycin	RNA and DNA synthesis inhibitor	A. Conv. Chemo	Approved	HY-17559	Medchem Express	100
Dactinomycin	RNA and DNA synthesis inhibitor	A. Conv. Chemo	Approved		Medchem Express	10
Daunorubicin	Topoisomerase II inhibitor	A. Conv. Chemo	Approved	HY-13062	Medchem Express	100
Daunorubicin	Topoisomerase II inhibitor	A. Conv. Chemo	Approved	HY-13062	Medchem Express	10
Docetaxel	Mitotic inhibitor, taxane microtubule stabilizer	A. Conv. Chemo	Approved	D-1000	LC Laboratories	100
Docetaxel	Mitotic inhibitor, taxane microtubule stabilizer	A. Conv. Chemo	Approved	D-1000	LC Laboratories	10
Doxorubicin	Topoisomerase II inhibitor	A. Conv. Chemo	Approved	D1515	Sigma-Aldrich	100
Doxorubicin	Topoisomerase II inhibitor	A. Conv. Chemo	Approved	D1515	Sigma-Aldrich	10
Epirubicin	Topoisomerase II inhibitor	A. Conv. Chemo	Approved	HY-13624A	Medchem Express	100
Epirubicin	Topoisomerase II inhibitor	A. Conv. Chemo	Approved	HY-13624A	Medchem Express	10
Eribulin	Mitotic inhibitor, microtubule depolymerizer.	A. Conv. Chemo	Approved	115457	Eisai Europe	100
Eribulin	Mitotic inhibitor, microtubule depolymerizer.	A. Conv. Chemo	Approved	115457	Eisai Europe	10
Etoposide	Topoisomerase II inhibitor	A. Conv. Chemo	Approved		Medchem Express	1000
Etoposide	Topoisomerase II inhibitor	A. Conv. Chemo	Approved		Medchem Express	100

Floxuridine	Antimetabolite; Analog of 5-fluorouracil	A. Conv. Chemo	Approved	HY-B0097	Medchem Express	1000
Floxuridine	Antimetabolite; Analog of 5-fluorouracil	A. Conv. Chemo	Approved		Medchem Express	100
Fludarabine	Antimetabolite; Purine analog	A. Conv. Chemo	Approved	HY-B0028	Medchem Express	1000
Fludarabine	Antimetabolite; Purine analog	A. Conv. Chemo	Approved	HY-B0028	Medchem Express	100
Fluorouracil	Antimetabolite	A. Conv. Chemo	Approved	HY-90006	Medchem Express	1000
Fluorouracil	Antimetabolite	A. Conv. Chemo	Approved	HY-90006	Medchem Express	100
Gemcitabine	Antimetabolite; Nucleoside analog	A. Conv. Chemo	Approved	HY-B0003	Medchem Express	100
Gemcitabine	Antimetabolite; Nucleoside analog	A. Conv. Chemo	Approved	HY-B0003	Medchem Express	10
Hydroxyurea	Antineoplastic agent	A. Conv. Chemo	Approved	H8627	Sigma-Aldrich	100000
Hydroxyurea	Antineoplastic agent	A. Conv. Chemo	Approved	H8627	Sigma-Aldrich	10000
Idarubicin	Topoisomerase II inhibitor	A. Conv. Chemo	Approved	I1656	Sigma-Aldrich	100
Idarubicin	Topoisomerase II inhibitor	A. Conv. Chemo	Approved	I1656	Sigma-Aldrich	10
Indibulin	Mitoric inhibitor. Microtubule depolymerizer	A. Conv. Chemo	Investigational (Ph 2)		Tocris Biosciences	1000
Indibulin	Mitoric inhibitor. Microtubule depolymerizer	A. Conv. Chemo	Investigational (Ph 2)		Tocris Biosciences	100
Ixabepilone	Mitotic inhibitor. Epothilone microtubule stabilizer.	A. Conv. Chemo	Approved	HY-10222	Medchem Express	100
Ixabepilone	Mitotic inhibitor. Epothilone microtubule stabilizer.	A. Conv. Chemo	Approved	HY-10222	Medchem Express	10
Mercaptopurine	Antimetabolite	A. Conv. Chemo	Approved	HY-13677	Medchem Express	1000
Mercaptopurine	Antimetabolite	A. Conv. Chemo	Approved		Medchem Express	100
Mitomycin C	Antineoplastic anatibiotic; DNA crosslinker	A. Conv. Chemo	Approved	HY-13316	Medchem Express	1000
Mitomycin C	Antineoplastic anatibiotic; DNA crosslinker	A. Conv. Chemo	Approved	HY-13316	Medchem Express	100
Mitotane	Antineoplastic agent	A. Conv. Chemo	Approved	HY-13690	Medchem Express	1000
Mitotane	Antineoplastic agent	A. Conv. Chemo	Approved		Medchem Express	100
Mitoxantrone	Topoisomerase II inhibitor	A. Conv. Chemo	Approved		Medchem Express	100
Mitoxantrone	Topoisomerase II inhibitor	A. Conv. Chemo	Approved		Medchem Express	10
Nelarabine	Nucleoside analog, DNA, RNA synth inhibitor	A. Conv. Chemo	Approved	SRP003328r	SequoiaResearchProducts	1000
Nelarabine	Nucleoside analog, DNA, RNA synth inhibitor	A. Conv. Chemo	Approved	SRP003328r	SequoiaResearchProducts	100
Omacetaxine	Protein synthesis inhib (80 S ribosome)	A. Conv. Chemo	Approved	sc-202652	Santa Cruz Biotechnology	1000
Omacetaxine	Protein synthesis inhib (80 S ribosome)	A. Conv. Chemo	Approved	sc-202652	Santa Cruz Biotechnology	100
Oxaliplatin	Platinum-based antineoplastic agent	A. Conv. Chemo	Approved	S1224-3	Selleck	10000
Oxaliplatin	Platinum-based antineoplastic agent	A. Conv. Chemo	Approved	S1224-3	Selleck	1000
Paclitaxel	Mitotic inhibitor, taxane microtubule stabilizer	A. Conv. Chemo	Approved	HY-B0015	Medchem Express	100
Paclitaxel	Mitotic inhibitor, taxane microtubule stabilizer	A. Conv. Chemo	Approved	HY-B0015	Medchem Express	10
Pentostatin	Antimetabolite; Purine analog	A. Conv. Chemo	Approved		Medchem Express	1000
Pentostatin	Antimetabolite; Purine analog	A. Conv. Chemo	Approved	HY-A0006	Medchem Express	100
Pixantrone	topoisomerase II inhibitor	A. Conv. Chemo	Investigational (Ph 3)	HY-13727A	Medchem Express	10000
Pixantrone	topoisomerase II inhibitor	A. Conv. Chemo	Investigational (Ph 3)	HY-13727A	Medchem Express	1000
Plicamycin	RNA synthesis inhibitor	A. Conv. Chemo	Approved	sc-200909-7	Santa Cruz Biotechnology	1000
Plicamycin	RNA synthesis inhibitor	A. Conv. Chemo	Approved		Santa Cruz Biotechnology	100
Raltitrexed	DHFR/GARFT/thymidylate synthase inhibitor	A. Conv. Chemo	Approved	HY-10821	Medchem Express	100
Raltitrexed	DHFR/GARFT/thymidylate synthase inhibitor	A. Conv. Chemo	Approved	HY-10821	Medchem Express	10
SN-38	Active metabolite of irinotecan. Topoisomerase I inhibitor	A. Conv. Chemo	(Approved)		ChemieTek	1000
SN-38	Active metabolite of irinotecan. Topoisomerase I inhibitor	A. Conv. Chemo	(Approved)	CT-SN38	ChemieTek	100
Temozolomide	Alkylating agent	A. Conv. Chemo	Approved	S1237	Selleck	10000
Temozolomide	Alkylating agent	A. Conv. Chemo	Approved	S1237	Selleck	1000
Teniposide	Topoisomerase II inhibitor	A. Conv. Chemo	Approved		Medchem Express	1000
Teniposide	Topoisomerase II inhibitor	A. Conv. Chemo	Approved		Medchem Express	100
Thioguanine	Antimetabolite; Purine analog	A. Conv. Chemo	Approved	HY-13765	Medchem Express	1000
Thioguanine	Antimetabolite; Purine analog	A. Conv. Chemo	Approved		Medchem Express	100
Topotecan	Topoisomerase I inhibitor. Camptothecin analog	A. Conv. Chemo	Approved	HY-13768A	Medchem Express	1000
Topotecan	Topoisomerase I inhibitor. Camptothecin analog	A. Conv. Chemo	Approved	HY-13768A	Medchem Express	100
Trifluridine	Antimetabolite; Nucleoside analog	A. Conv. Chemo	Approved	HY-A0061	Medchem Express	1000
Trifluridine	Antimetabolite; Nucleoside analog	A. Conv. Chemo	Approved	HY-A0061	Medchem Express	100
Valrubicin	Topoisomerase II inhibitor	A. Conv. Chemo	Approved	HY-13772	Medchem Express	500

Valrubicin	Topoisomerase II inhibitor	A. Conv. Chemo	Approved	HY-13772	Medchem Express	50
Vinblastine	Mitotic inhibitor. Vinca alkaloid microtubule depolymerizer	A. Conv. Chemo	Approved	HY-13780	Medchem Express	100
Vinblastine	Mitotic inhibitor. Vinca alkaloid microtubule depolymerizer	A. Conv. Chemo	Approved	HY-13780	Medchem Express	10
Vincristine	Mitotic inhibitor. Vinca alkaloid microtubule depolymerizer	A. Conv. Chemo	Approved	S1241	Selleck	100
Vincristine	Mitotic inhibitor. Vinca alkaloid microtubule depolymerizer	A. Conv. Chemo	Approved	S1241	Selleck	10
Vinflunine	Mitotic inhibitor. Vinca alkaloid microtubule depolymerizer	A. Conv. Chemo	Approved	HY-B0628A	Medchem Express	100
Vinflunine	Mitotic inhibitor. Vinca alkaloid microtubule depolymerizer	A. Conv. Chemo	Approved	HY-B0628A	Medchem Express	10
Vinorelbine	Mitotic inhibitor. Vinca alkaloid microtubule depolymerizer	A. Conv. Chemo	Approved	S4269	Selleck	1000
Vinorelbine	Mitotic inhibitor. Vinca alkaloid microtubule depolymerizer	A. Conv. Chemo	Approved	S4269	Selleck	100
A-419259	HCK and other SRC family kinase inhibitor	B. Kinase inhibitor	Probe		Medchem Express	1000
A-419259	HCK and other SRC family kinase inhibitor	B. Kinase inhibitor	Probe		Medchem Express	100
ABC294640	Sphingosine kinase 2 inhibitor	B. Kinase inhibitor	Investigational (Ph 2)		Selleck	5000
ABC294640	Sphingosine kinase 2 inhibitor	B. Kinase inhibitor	Investigational (Ph 2)		Selleck	500
Abemaciclib	CDK4/6 inhibitor	B. Kinase inhibitor	Investigational (Ph 3)		Medchem Express	250
Abemaciclib	CDK4/6 inhibitor	B. Kinase inhibitor	Investigational (Ph 3)		Medchem Express	25
Acalabrutinib	BTK inhibitor	B. Kinase inhibitor	Investigational (Ph 3)		ChemieTek	100
Acalabrutinib	BTK inhibitor	B. Kinase inhibitor	Investigational (Ph 3)		ChemieTek	10
Afatinib	EGFR inhibitor	B. Kinase inhibitor	Approved	S1011	Selleck	100
Afatinib	EGFR inhibitor	B. Kinase inhibitor	Approved	S1011	Selleck	10
Afuresertib	AKT1-selective inhibitor	B. Kinase inhibitor	Investigational (Ph 2)		Medchem Express	100
Afuresertib	AKT1-selective inhibitor	B. Kinase inhibitor	Investigational (Ph 2)		Medchem Express	10
Alectinib	ALK (incl gatekeeper mut) inhib	B. Kinase inhibitor	Approved (Japan, US)	CT-CH542	ChemieTek	100
Alectinib	ALK (incl gatekeeper mut) inhib	B. Kinase inhibitor	Approved (Japan, US)		ChemieTek	10
Alisertib	Aurora A inhibitor	B. Kinase inhibitor	Investigational (Ph 3)	HY-10971	Medchem Express	1000
Alisertib	Aurora A inhibitor	B. Kinase inhibitor	Investigational (Ph 3)	HY-10971	Medchem Express	100
Alpelisib	PI3Kalpha selective inhibitor	B. Kinase inhibitor	Investigational (Ph 2)		Selleck	250
Alpelisib	PI3Kalpha selective inhibitor	B. Kinase inhibitor	Investigational (Ph 2)		Selleck	25
Altiratinib	MET/Tie-2 inhibitor	B. Kinase inhibitor	Investigational (Ph 1)		ChemieTek	1000
Altiratinib	MET/Tie-2 inhibitor	B. Kinase inhibitor	Investigational (Ph 1)		ChemieTek	100
Alvocidib	CDK inhibitor	B. Kinase inhibitor	Investigational (Ph 2)		Selleck	1000
Alvocidib	CDK inhibitor	B. Kinase inhibitor	Investigational (Ph 2)		Selleck	100
Amcasertib	Cancer stem cell kinase inhibitor	B. Kinase inhibitor	Investigational (Ph 2)		Medchem Express	1000
Amcasertib	Cancer stem cell kinase inhibitor	B. Kinase inhibitor	Investigational (Ph 2)		Medchem Express	100
AMG-337	Met inhibitor	B. Kinase inhibitor	Investigational (Ph 2)		Medchem Express	1000
AMG-337	Met inhibitor	B. Kinase inhibitor	Investigational (Ph 2)		Medchem Express	100
AMG-925	FLT-3, CDK4 inhibitor	B. Kinase inhibitor	Probe		ChemieTek	100
AMG-925	FLT-3, CDK4 inhibitor	B. Kinase inhibitor	Probe		ChemieTek	10
AMG319	PI3Kdelta inhibitor	B. Kinase inhibitor	Investigational (Ph 2)		Selleck	100
AMG319	PI3Kdelta inhibitor	B. Kinase inhibitor	Investigational (Ph 2)		Selleck	10
Amuvatinib	Broad spectrum TK inhib	B. Kinase inhibitor	Investigational (Ph 2)		Selleck	1000
Amuvatinib	Broad spectrum TK inhib	B. Kinase inhibitor	Investigational (Ph 2)		Selleck	100
Apatinib	VEGFR inhibitor	B. Kinase inhibitor	Investigational (Ph 3)	S2221	Selleck	1000
Apatinib	VEGFR inhibitor	B. Kinase inhibitor	Investigational (Ph 3)	S2221	Selleck	100
Asciminib	Allosteric ABL inhibitor, blocks myristoyl binding	B. Kinase inhibitor	Investigational (Ph 1)		Active Biochem	100
Asciminib	Allosteric ABL inhibitor, blocks myristoyl binding	B. Kinase inhibitor	Investigational (Ph 1)		Active Biochem	10
ASP3026	ALK inhibitor	B. Kinase inhibitor	Investigational (Ph 1)		ChemieTek	1000
ASP3026	ALK inhibitor	B. Kinase inhibitor	Investigational (Ph 1)		ChemieTek	100
AT13148	p70S6K, PKA, ROCK (AKT) inhibitor	B. Kinase inhibitor	Investigational (Ph 1)		Medchem Express	1000
AT13148	p70S6K, PKA, ROCK (AKT) inhibitor	B. Kinase inhibitor	Investigational (Ph 1)		Medchem Express	100
AT7519	CDK1, 2, 4, 6 and 9 inhibitor	B. Kinase inhibitor	Investigational (Ph 1)		Selleck	1000
AT7519	CDK1, 2, 4, 6 and 9 inhibitor	B. Kinase inhibitor	Investigational (Ph 1)		Selleck	100
AT9283	Aurora A & B, Jak2, Flt, Abl inhibitor	B. Kinase inhibitor	Investigational (Ph 2)		Selleck	100
AT9283	Aurora A & B, Jak2, Flt, Abl inhibitor	B. Kinase inhibitor	Investigational (Ph 2)		Selleck	10

Axitinib	VEGFR, PDGFR, KIT inhibitor	B. Kinase inhibitor	Approved	A-1107	LC Laboratories	1000
Axitinib	VEGFR, PDGFR, KIT inhibitor	B. Kinase inhibitor	Approved		LC Laboratories	100
AZ 3146	Mps1 kinase (TTK) inhibitor	B. Kinase inhibitor	Probe	3994	Tocris Biosciences	1000
AZ 3146	Mps1 kinase (TTK) inhibitor	B. Kinase inhibitor	Probe	3994	Tocris Biosciences	100
AZ191	DYRK1A inhibitor	B. Kinase inhibitor	Probe		Selleck	1000
AZ191	DYRK1A inhibitor	B. Kinase inhibitor	Probe		Selleck	100
AZD-1080	GSK3 inhibitor	B. Kinase inhibitor	Investigational (Ph 1)		Selleck	1000
AZD-1080	GSK3 inhibitor	B. Kinase inhibitor	Investigational (Ph 1)		Selleck	100
AZD-5363	AKT inhibitor	B. Kinase inhibitor	Investigational (Ph 2)		ChemieTek	1000
AZD-5363	AKT inhibitor	B. Kinase inhibitor	Investigational (Ph 2)		ChemieTek	100
AZD-5438	CDK1,2,9 inhibitor	B. Kinase inhibitor	Investigational (Ph 1)		Selleck	1000
AZD-5438	CDK1,2,9 inhibitor	B. Kinase inhibitor	Investigational (Ph 1)		Selleck	100
AZD-6482	PI3Kbeta-selective inhibitor	B. Kinase inhibitor	Investigational (Ph 1)		Selleck	250
AZD-6482	PI3Kbeta-selective inhibitor	B. Kinase inhibitor	Investigational (Ph 1)		Selleck	25
AZD-8186	PI3Kbeta inhibitor	B. Kinase inhibitor	Investigational (Ph 1)		Active Biochem	100
AZD-8186	PI3Kbeta inhibitor	B. Kinase inhibitor	Investigational (Ph 1)		Active Biochem	10
AZD0156	ATM inihibitor	B. Kinase inhibitor	Investigational (Ph 1)		ChemieTek	100
AZD0156	ATM inihibitor	B. Kinase inhibitor	Investigational (Ph 1)		ChemieTek	10
AZD1152-HQPA	Aurora B inhibitor	B. Kinase inhibitor	Investigational (Ph 3)	CT-A1152H	ChemieTek	100
AZD1152-HQPA	Aurora B inhibitor	B. Kinase inhibitor	Investigational (Ph 3)	CT-A1152H	ChemieTek	10
AZD1208	PIM1, 2, 3 kinase inhibitor	B. Kinase inhibitor	Investigational (Ph 1)		Medchem Express	1000
AZD1208	PIM1, 2, 3 kinase inhibitor	B. Kinase inhibitor	Investigational (Ph 1)		Medchem Express	100
AZD1480	JAK1/2, FGFR inhibitor	B. Kinase inhibitor	Investigational (Ph 1)	CT-A1480	ChemieTek	100
AZD1480	JAK1/2, FGFR inhibitor	B. Kinase inhibitor	Investigational (Ph 1)		ChemieTek	10
AZD1775	Wee1 inhibitor	B. Kinase inhibitor	Investigational (Ph 2)		ChemieTek	1000
AZD1775	Wee1 inhibitor	B. Kinase inhibitor	Investigational (Ph 2)		ChemieTek	100
AZD3759	EGFR inhibitor, BBB penetrable	B. Kinase inhibitor	Investigational (Ph 2)		Medchem Express	100
AZD3759	EGFR inhibitor, BBB penetrable	B. Kinase inhibitor	Investigational (Ph 2)		Medchem Express	10
AZD4547	FGFR inhibitor	B. Kinase inhibitor	Investigational (Ph 2)		ChemieTek	100
AZD4547	FGFR inhibitor	B. Kinase inhibitor	Investigational (Ph 2)		ChemieTek	10
AZD6738	ATR inihibitor	B. Kinase inhibitor	Investigational (Ph 1)		Medchem Express	2500
AZD6738	ATR inihibitor	B. Kinase inhibitor	Investigational (Ph 1)		Medchem Express	250
AZD7545	PDHK inhibitor	B. Kinase inhibitor	Probe		Selleck	1000
AZD7762	Chk1 inhibitor	B. Kinase inhibitor	Investigational (Ph 1)	Axon 1399	Axon Medchem	100
AZD7762	Chk1 inhibitor	B. Kinase inhibitor	Investigational (Ph 1)	Axon 1399	Axon Medchem	10
AZD8055	mTOR inhibitor	B. Kinase inhibitor	Investigational (Ph 1)		ChemieTek	1000
AZD8055	mTOR inhibitor	B. Kinase inhibitor	Investigational (Ph 1)		ChemieTek	100
Bafetinib	Abl, Lyn inhibitor	B. Kinase inhibitor	Investigational (Ph 2)		Selleck	100
Bafetinib	Abl, Lyn inhibitor	B. Kinase inhibitor	Investigational (Ph 2)		Selleck	10
Baricitinib	JAK inhibitor	B. Kinase inhibitor	Approved (EU)		Selleck	250
Baricitinib	JAK inhibitor	B. Kinase inhibitor	Approved (EU)		Selleck	25
Bentamapimod	JNK inhibitor	B. Kinase inhibitor	Investigational (Ph 1)		Medchem Express	1000
Bentamapimod	JNK inhibitor	B. Kinase inhibitor	Investigational (Ph 1)		Medchem Express	100
BGB-283	Raf inhibitor	B. Kinase inhibitor	Investigational (Ph 1)		Active Biochem	1000
BGB-283	Raf inhibitor	B. Kinase inhibitor	Investigational (Ph 1)		Active Biochem	100
BGB324	Axl inhibitor	B. Kinase inhibitor	Investigational (Ph 1)		Medchem Express	1000
BGB324	Axl inhibitor	B. Kinase inhibitor	Investigational (Ph 1)		Medchem Express	100
BI 2536	PLK1 inhibitor	B. Kinase inhibitor	Investigational (Ph 2)		Selleck	100
BI 2536	PLK1 inhibitor	B. Kinase inhibitor	Investigational (Ph 2)		Selleck	10
Binimetinib	MEK1/2 inhibitor	B. Kinase inhibitor	Investigational (Ph 2)		ChemieTek	100
Binimetinib	MEK1/2 inhibitor	B. Kinase inhibitor	Investigational (Ph 2)		ChemieTek	10
BMS-754807	IGF1R inhibitor	B. Kinase inhibitor	Investigational (Ph 2)		Medchem Express	1000
BMS-754807	IGF1R inhibitor	B. Kinase inhibitor	Investigational (Ph 2)		Medchem Express	100

BMS-777607	Met, Axl, Ron and Tyro3 inhibitor	B. Kinase inhibitor	Investigational (Ph 2)		Selleck	250
BMS-777607	Met, Axl, Ron and Tyro3 inhibitor	B. Kinase inhibitor	Investigational (Ph 2)		Selleck	25
BMS-911543	JAK2 inhibitor	B. Kinase inhibitor	Investigational (Ph 1)		ChemieTek	1000
BMS-911543	JAK2 inhibitor	B. Kinase inhibitor	Investigational (Ph 1)		ChemieTek	100
BMS863233	Cdc7 inhibitor	B. Kinase inhibitor	Investigational (Ph 2)	S7547	Selleck	10000
BMS863233	Cdc7 inhibitor	B. Kinase inhibitor	Investigational (Ph 2)		Selleck	1000
Bosutinib	Abl, Src inhibitor	B. Kinase inhibitor	Approved		LC Laboratories	1000
Bosutinib	Abl, Src inhibitor	B. Kinase inhibitor	Approved	B-1788	LC Laboratories	100
Brigatinib	ALK inhibitor, including gatekeeper mutant ALK	B. Kinase inhibitor	Approved (US)		ChemieTek	100
Brigatinib	ALK inhibitor, including gatekeeper mutant ALK	B. Kinase inhibitor	Approved (US)		ChemieTek	10
Brivanib	VEGFR inhibitor	B. Kinase inhibitor	Investigational (Ph 3)		Selleck	100
Brivanib	VEGFR inhibitor	B. Kinase inhibitor	Investigational (Ph 3)		Selleck	10
Bryostatin 1	PKC activator	B. Kinase inhibitor	Investigational (Ph 1)		Santa Cruz Biotechnology	10
Bryostatin 1	PKC activator	B. Kinase inhibitor	Investigational (Ph 1)		Santa Cruz Biotechnology	1
Buparlisib	PI3K inhibitor, pan-class I	B. Kinase inhibitor	Investigational (Ph 2)		Selleck	1000
Buparlisib	PI3K inhibitor, pan-class I	B. Kinase inhibitor	Investigational (Ph 2)		Selleck	100
BX-912	PDK1 inhib	B. Kinase inhibitor	Probe		Selleck	1000
BX-912	PDK1 inhib	B. Kinase inhibitor	Probe		Selleck	100
Cabozantinib	VEGFR2, Met, FLT3, Tie2, Kit and Ret inhibitor	B. Kinase inhibitor	Approved	CT-XL184	ChemieTek	100
Cabozantinib	VEGFR2, Met, FLT3, Tie2, Kit and Ret inhibitor	B. Kinase inhibitor	Approved		ChemieTek	10
Canertinib	pan-HER inhibitor	B. Kinase inhibitor	Investigational (Ph 3)	C-1201	LC Laboratories	1000
Canertinib	pan-HER inhibitor	B. Kinase inhibitor	Investigational (Ph 3)	C-1201	LC Laboratories	100
Capmatinib	MET inhibitor	B. Kinase inhibitor	Investigational (Ph 2)		Selleck	100
Capmatinib	MET inhibitor	B. Kinase inhibitor	Investigational (Ph 2)		Selleck	10
CC-115	mTOR/DNA-PK inhibitor	B. Kinase inhibitor	Investigational (Ph 1)		Medchem Express	1000
CC-115	mTOR/DNA-PK inhibitor	B. Kinase inhibitor	Investigational (Ph 1)		Medchem Express	100
CC-223	mTOR inhibitor	B. Kinase inhibitor	Investigational (Ph 2)		Medchem Express	1000
CC-223	mTOR inhibitor	B. Kinase inhibitor	Investigational (Ph 2)		Medchem Express	100
CCT196969	pan-RAF/Src inhibitor	B. Kinase inhibitor	Probe		ChemieTek	2500
CCT196969	pan-RAF/Src inhibitor	B. Kinase inhibitor	Probe		ChemieTek	250
Cediranib	KDR/Flt/VEGFR inhibitor	B. Kinase inhibitor	Investigational (Ph 3)	S1017	Selleck	100
Cediranib	KDR/Flt/VEGFR inhibitor	B. Kinase inhibitor	Investigational (Ph 3)	S1017	Selleck	10
CEP-32496	BRAF inhibitor	B. Kinase inhibitor	Investigational (Ph 2)		Selleck	1000
CEP-32496	BRAF inhibitor	B. Kinase inhibitor	Investigational (Ph 2)		Selleck	100
CEP-37440	ALK inhibitor	B. Kinase inhibitor	Investigational (Ph 1)		ChemieTek	500
CEP-37440	ALK inhibitor	B. Kinase inhibitor	Investigational (Ph 1)		ChemieTek	50
Cerdulatinib	JAK, SYK inhibitor	B. Kinase inhibitor	Investigational (Ph 1)		Medchem Express	1000
Cerdulatinib	JAK, SYK inhibitor	B. Kinase inhibitor	Investigational (Ph 1)		Medchem Express	100
Ceritinib	ALK inhibitor	B. Kinase inhibitor	Approved	S7083	Selleck	250
Ceritinib	ALK inhibitor	B. Kinase inhibitor	Approved	S7083	Selleck	25
Cobimetinib	MEK1/2 inhibitor	B. Kinase inhibitor	Approved (US)	HY-13064	Medchem Express	100
Cobimetinib	MEK1/2 inhibitor	B. Kinase inhibitor	Approved (US)	HY-13064	Medchem Express	10
Copanlisib	PI3K alpha, delta selective inhibitor	B. Kinase inhibitor	Investigational (Ph 2)		Selleck	100
Copanlisib	PI3K alpha, delta selective inhibitor	B. Kinase inhibitor	Investigational (Ph 2)		Selleck	10
Crenolanib	PDGFRA and PDGFRB inhibitor	B. Kinase inhibitor	Investigational (Ph 2)		Selleck	1000
Crenolanib	PDGFRA and PDGFRB inhibitor	B. Kinase inhibitor	Investigational (Ph 2)		Selleck	100
Crizotinib	ALK, c-Met inhibitor	B. Kinase inhibitor	Approved	S1068	Selleck	100
Crizotinib	ALK, c-Met inhibitor	B. Kinase inhibitor	Approved	S1068	Selleck	10
Dabrafenib	B-Raf(V600E) inhibitor	B. Kinase inhibitor	Approved	CT-DABR	ChemieTek	250
Dabrafenib	B-Raf(V600E) inhibitor	B. Kinase inhibitor	Approved	CT-DABR	ChemieTek	25
Dacomitinib	pan-HER inhibitor	B. Kinase inhibitor	Investigational (Ph 3)		ChemieTek	100
Dacomitinib	pan-HER inhibitor	B. Kinase inhibitor	Investigational (Ph 3)		ChemieTek	10
Dactolisib	mTOR/(PI3K) inhibitor	B. Kinase inhibitor	Investigational (Ph 2)		LC Laboratories	100

Dactolisib	mTOR/(PI3K) inhibitor	B. Kinase inhibitor	Investigational (Ph 2)		LC Laboratories	10
Danusertib	Aurora, Ret, TrkA, FGFR-1 inhibitor	B. Kinase inhibitor	Investigational (Ph 2)		Medchem Express	1000
Danusertib	Aurora, Ret, TrkA, FGFR-1 inhibitor	B. Kinase inhibitor	Investigational (Ph 2)		Medchem Express	100
Dasatinib	Abl, Src, Kit, EphR... Inhibitor	B. Kinase inhibitor	Approved	D-3307	LC Laboratories	100
Dasatinib	Abl, Src, Kit, EphR... Inhibitor	B. Kinase inhibitor	Approved	D-3307	LC Laboratories	10
Decernotinib	JAK3 inhibitor	B. Kinase inhibitor	Investigational (Ph 3)		ChemieTek	1000
Decernotinib	JAK3 inhibitor	B. Kinase inhibitor	Investigational (Ph 3)		ChemieTek	100
DEL-22379	ERK dimerization inhibitor	B. Kinase inhibitor	Probe		Selleck	5000
DEL-22379	ERK dimerization inhibitor	B. Kinase inhibitor	Probe		Selleck	500
Dinaciclib	CDK inhibitor	B. Kinase inhibitor	Investigational (Ph 3)		ChemieTek	100
Dinaciclib	CDK inhibitor	B. Kinase inhibitor	Investigational (Ph 3)		ChemieTek	10
Doramapimod	p38MAPK inhibitor	B. Kinase inhibitor	Investigational (Ph 1)		Medchem Express	1000
Doramapimod	p38MAPK inhibitor	B. Kinase inhibitor	Investigational (Ph 1)		Medchem Express	100
Dovitinib	FGFR inhibitor	B. Kinase inhibitor	Investigational (Ph 3)	S1018	Selleck	1000
Dovitinib	FGFR inhibitor	B. Kinase inhibitor	Investigational (Ph 3)	S1018	Selleck	100
Duvelisib	PI3K inhibitor	B. Kinase inhibitor	Investigational (Ph 3)		Selleck	50
Duvelisib	PI3K inhibitor	B. Kinase inhibitor	Investigational (Ph 3)		Selleck	5
Encorafenib	B-Raf(V600E) inhibitor	B. Kinase inhibitor	Investigational (Ph 2)		Selleck	100
Encorafenib	B-Raf(V600E) inhibitor	B. Kinase inhibitor	Investigational (Ph 2)		Selleck	10
ENMD-2076	pan-Aurora, VEGFR inhibitor	B. Kinase inhibitor	Investigational (Ph 2)		Selleck	1000
ENMD-2076	pan-Aurora, VEGFR inhibitor	B. Kinase inhibitor	Investigational (Ph 2)		Selleck	100
Ensartinib	ALK inhibitor	B. Kinase inhibitor	Investigational (Ph 3)		Medchem Express	100
Ensartinib	ALK inhibitor	B. Kinase inhibitor	Investigational (Ph 3)		Medchem Express	10
Entospletinib	SYK inhibitor	B. Kinase inhibitor	Investigational (Ph 2)		Selleck	500
Entospletinib	SYK inhibitor	B. Kinase inhibitor	Investigational (Ph 2)		Selleck	50
Entrectinib	TRK, ROS1, ALK inhibitor	B. Kinase inhibitor	Investigational (Ph 2)		Medchem Express	100
Entrectinib	TRK, ROS1, ALK inhibitor	B. Kinase inhibitor	Investigational (Ph 2)		Medchem Express	10
Enzastaurin	PKCbeta inhibitor	B. Kinase inhibitor	Investigational (Ph 3)	E-4506	LC Laboratories	1000
Enzastaurin	PKCbeta inhibitor	B. Kinase inhibitor	Investigational (Ph 3)	E-4506	LC Laboratories	100
Erdafitinib	FGFR inhibitor	B. Kinase inhibitor	Investigational (Ph 2)		Medchem Express	100
Erdafitinib	FGFR inhibitor	B. Kinase inhibitor	Investigational (Ph 2)		Medchem Express	10
Erlotinib	EGFR inhibitor	B. Kinase inhibitor	Approved	HY-50896	Medchem Express	1000
Erlotinib	EGFR inhibitor	B. Kinase inhibitor	Approved	HY-50896	Medchem Express	100
Fedratinib	JAK2-selective inhibitor	B. Kinase inhibitor	Investigational (Ph 2)		ChemieTek	1000
Fedratinib	JAK2-selective inhibitor	B. Kinase inhibitor	Investigational (Ph 2)		ChemieTek	100
Filgotinib	JAK1-selective inhibitor	B. Kinase inhibitor	Investigational (Ph 2)		Selleck	1000
Filgotinib	JAK1-selective inhibitor	B. Kinase inhibitor	Investigational (Ph 2)		Selleck	100
Foretinib	MET, VEGFR2 inhibitor	B. Kinase inhibitor	Investigational (Ph 2)	S1111	Selleck	100
Foretinib	MET, VEGFR2 inhibitor	B. Kinase inhibitor	Investigational (Ph 2)	S1111	Selleck	10
Fostamatinib	Syk inhibitor	B. Kinase inhibitor	Investigational (Ph 2)	S2206-2	Selleck	10000
Fostamatinib	Syk inhibitor	B. Kinase inhibitor	Investigational (Ph 2)	S2206-2	Selleck	1000
FRAX486	PAK1, 2, 3 inhibitor	B. Kinase inhibitor	Probe		ChemieTek	500
FRAX486	PAK1, 2, 3 inhibitor	B. Kinase inhibitor	Probe		ChemieTek	50
Galunisertib	TGF-B/Smad inhibitor	B. Kinase inhibitor	Investigational (Ph 2)		Selleck	100
Galunisertib	TGF-B/Smad inhibitor	B. Kinase inhibitor	Investigational (Ph 2)		Selleck	10
Gandotinib	JAK2 inhibitor	B. Kinase inhibitor	Investigational (Ph 2)		Medchem Express	1000
Gandotinib	JAK2 inhibitor	B. Kinase inhibitor	Investigational (Ph 2)		Medchem Express	100
GDC-0084	PI3K/mTOR inhibitor	B. Kinase inhibitor	Investigational (Ph 1)		Medchem Express	1000
GDC-0084	PI3K/mTOR inhibitor	B. Kinase inhibitor	Investigational (Ph 1)		Medchem Express	100
GDC-0623	MEK1/2 inhibitor	B. Kinase inhibitor	Investigational (Ph 2)		Active Biochem	250
GDC-0623	MEK1/2 inhibitor	B. Kinase inhibitor	Investigational (Ph 2)		Active Biochem	25
GDC-0853	BTK inhibitor	B. Kinase inhibitor	Investigational (Ph 2)		Medchem Express	100
GDC-0853	BTK inhibitor	B. Kinase inhibitor	Investigational (Ph 2)		Medchem Express	10

Gedatolisib	PI3K/mTOR inhibitor	B. Kinase inhibitor	Investigational (Ph 2))		Selleck	100
Gedatolisib	PI3K/mTOR inhibitor	B. Kinase inhibitor	Investigational (Ph 2))		Selleck	10
Gefitinib	EGFR inhibitor	B. Kinase inhibitor	Approved	G-4408	LC Laboratories	1000
Gefitinib	EGFR inhibitor	B. Kinase inhibitor	Approved	G-4408	LC Laboratories	100
Gilteritinib	FLT3/AXL inhibitor	B. Kinase inhibitor	Investigational (Ph 2)		ChemieTek	100
Gilteritinib	FLT3/AXL inhibitor	B. Kinase inhibitor	Investigational (Ph 2)		ChemieTek	10
Glesatinib	MET, AXL, TIE, VEGFR, RON inhibitor	B. Kinase inhibitor	Investigational (Ph 2)		ChemieTek	250
Glesatinib	MET, AXL, TIE, VEGFR, RON inhibitor	B. Kinase inhibitor	Investigational (Ph 2)		ChemieTek	25
GNE-0877	LRRK2 inhibitor	B. Kinase inhibitor	Probe		Selleck	100
GNE-0877	LRRK2 inhibitor	B. Kinase inhibitor	Probe		Selleck	10
GNE-7915	LRRK2 inhibitor	B. Kinase inhibitor	Probe		ChemieTek	100
Golvatinib	MET, VEGFR2 inhibitor	B. Kinase inhibitor	Investigational (Ph 2)		Selleck	250
Golvatinib	MET, VEGFR2 inhibitor	B. Kinase inhibitor	Investigational (Ph 2)		Selleck	25
GSK-1070916	AURb, AURc inhibitor	B. Kinase inhibitor	Investigational (Ph 1)		Selleck	100
GSK-1070916	AURb, AURc inhibitor	B. Kinase inhibitor	Investigational (Ph 1)		Selleck	10
GSK-2334470	PDK1 inhibitor	B. Kinase inhibitor	Probe		ChemieTek	1000
GSK-2334470	PDK1 inhibitor	B. Kinase inhibitor	Probe		ChemieTek	100
GSK-461364	PLK1 inhibitor	B. Kinase inhibitor	Investigational (Ph 1)		Medchem Express	1000
GSK-461364	PLK1 inhibitor	B. Kinase inhibitor	Investigational (Ph 1)		Medchem Express	100
GSK-690693	AKT, PKA, PKC inhibitor	B. Kinase inhibitor	Investigational (Ph 1)		Selleck	1000
GSK-690693	AKT, PKA, PKC inhibitor	B. Kinase inhibitor	Investigational (Ph 1)		Selleck	100
GSK2256098	FAK inhibitor	B. Kinase inhibitor	Investigational (Ph 2)		Medchem Express	100
GSK2256098	FAK inhibitor	B. Kinase inhibitor	Investigational (Ph 2)		Medchem Express	10
GSK2636771	PI3K beta selective inhibitor	B. Kinase inhibitor	Investigational (Ph 1)		ChemieTek	1000
GSK2636771	PI3K beta selective inhibitor	B. Kinase inhibitor	Investigational (Ph 1)		ChemieTek	100
GSK2656157	PERK inhibitor	B. Kinase inhibitor	Probe		Medchem Express	250
GSK2656157	PERK inhibitor	B. Kinase inhibitor	Probe		Medchem Express	25
GSK269962	ROCK1 and ROCK2 inhibitor	B. Kinase inhibitor	Probe		Tocris Biosciences	1000
GSK269962	ROCK1 and ROCK2 inhibitor	B. Kinase inhibitor	Probe		Tocris Biosciences	100
GSK650394	SGK1 & 2 inhibitor	B. Kinase inhibitor	Probe		Tocris Biosciences	100
Hydroxyfasudil	ROCK, PKA, PKG, PRK inhibitor	B. Kinase inhibitor	(Approved Japan)	sc-202176	Santa Cruz Biotechnology	1900
Hydroxyfasudil	ROCK, PKA, PKG, PRK inhibitor	B. Kinase inhibitor	(Approved Japan)	sc-202176	Santa Cruz Biotechnology	190
Ibrutinib	Btk inhibitor	B. Kinase inhibitor	Approved	S2680	Selleck	100
Ibrutinib	Btk inhibitor	B. Kinase inhibitor	Approved	S2680	Selleck	10
Icotinib	EGFR inhibitor	B. Kinase inhibitor	Investigational (Ph 2)		Selleck	1000
Icotinib	EGFR inhibitor	B. Kinase inhibitor	Investigational (Ph 2)		Selleck	100
Idelalisib	PI3K inhibitor, p110δ-selective	B. Kinase inhibitor	Approved	CT-CAL101-[illegible]	ChemieTek	1000
Idelalisib	PI3K inhibitor, p110δ-selective	B. Kinase inhibitor	Approved	CT-CAL101-[illegible]	ChemieTek	100
Imatinib	Abl, Kit, PDGFRB inhibitor	B. Kinase inhibitor	Approved	C-5508	LC Laboratories	1000
Imatinib	Abl, Kit, PDGFRB inhibitor	B. Kinase inhibitor	Approved	C-5508	LC Laboratories	100
Infigratinib	FGFR inhibitor	B. Kinase inhibitor	Investigational (Ph 1)		ChemieTek	100
Infigratinib	FGFR inhibitor	B. Kinase inhibitor	Investigational (Ph 1)		ChemieTek	10
Ipatasertib	AKT inhibitor	B. Kinase inhibitor	Investigational (Ph 2)		ChemieTek	1000
Ipatasertib	AKT inhibitor	B. Kinase inhibitor	Investigational (Ph 2)		ChemieTek	100
KD025	ROCK2 inhibitor	B. Kinase inhibitor	Investigational (Ph 2)		Medchem Express	500
KD025	ROCK2 inhibitor	B. Kinase inhibitor	Investigational (Ph 2)		Medchem Express	50
KU-60019	ATM inhibitor	B. Kinase inhibitor	Probe		Selleck	2500
KU-60019	ATM inhibitor	B. Kinase inhibitor	Probe		Selleck	250
Lapatinib	HER2, EGFR inhibitor	B. Kinase inhibitor	Approved	L-4804	LC Laboratories	100
Lapatinib	HER2, EGFR inhibitor	B. Kinase inhibitor	Approved	L-4804	LC Laboratories	10
Larotrectinib	TRK inhibitor	B. Kinase inhibitor	Investigational (Ph 2)		Medchem Express	100
Larotrectinib	TRK inhibitor	B. Kinase inhibitor	Investigational (Ph 2)		Medchem Express	10
Lenvatinib	VEGFR inhibitor	B. Kinase inhibitor	Approved (US)	S1164	Selleck	250

Lenvatinib	VEGFR inhibitor	B. Kinase inhibitor	Approved (US)	S1164	Selleck	25
Linifanib	VEGFR, PDGFR, CSF-1R, FLT3 inhibitor	B. Kinase inhibitor	Investigational (Ph 3)		Selleck	100
Linifanib	VEGFR, PDGFR, CSF-1R, FLT3 inhibitor	B. Kinase inhibitor	Investigational (Ph 3)		Selleck	10
Linsitinib	IGF1R, IR inhibitor	B. Kinase inhibitor	Investigational (Ph 2)		ChemieTek	1000
Linsitinib	IGF1R, IR inhibitor	B. Kinase inhibitor	Investigational (Ph 2)		ChemieTek	100
Losmapimod	p38MAPK inhibitor	B. Kinase inhibitor	Investigational (Ph 3)		Selleck	1000
Losmapimod	p38MAPK inhibitor	B. Kinase inhibitor	Investigational (Ph 3)		Selleck	100
Lucitanib	FGFR1, VEGFR inhibitor	B. Kinase inhibitor	Investigational (Ph 2)		Axon Medchem	1000
Lucitanib	FGFR1, VEGFR inhibitor	B. Kinase inhibitor	Investigational (Ph 2)		Axon Medchem	100
LY-2584702	p70S6K inhibitor	B. Kinase inhibitor	Investigational (Ph 1)		Medchem Express	1000
LY-2584702	p70S6K inhibitor	B. Kinase inhibitor	Investigational (Ph 1)		Medchem Express	100
LY-2874455	FGFR inhibitor	B. Kinase inhibitor	Investigational (Ph 1)		Axon Medchem	100
LY-2874455	FGFR inhibitor	B. Kinase inhibitor	Investigational (Ph 1)		Axon Medchem	10
LY3009120	pan-RAF inhibitor	B. Kinase inhibitor	Investigational (Ph 1)		Medchem Express	1000
LY3009120	pan-RAF inhibitor	B. Kinase inhibitor	Investigational (Ph 1)		Medchem Express	100
LY3023414	PI3K/mTOR/DNA-PK inhibitor	B. Kinase inhibitor	Investigational (Ph 2)		Medchem Express	250
LY3023414	PI3K/mTOR/DNA-PK inhibitor	B. Kinase inhibitor	Investigational (Ph 2)		Medchem Express	25
Masitinib	KIT inhibitor	B. Kinase inhibitor	Investigational (Ph 3)	M-7007	LC Laboratories	1000
Masitinib	KIT inhibitor	B. Kinase inhibitor	Investigational (Ph 3)	M-7007	LC Laboratories	100
Merestinib	Met inhibitor	B. Kinase inhibitor	Investigational (Ph 2)		Medchem Express	100
Merestinib	Met inhibitor	B. Kinase inhibitor	Investigational (Ph 2)		Medchem Express	10
Midostaurin	Broad TK (FLT3, KIT, RET, JAK, EGFR...) inhibitor	B. Kinase inhibitor	Approved (US)	HY-10230	Medchem Express	1000
Midostaurin	Broad TK (FLT3, KIT, RET, JAK, EGFR...) inhibitor	B. Kinase inhibitor	Approved (US)	HY-10230	Medchem Express	100
Milciclib	CDK2 inhibitor	B. Kinase inhibitor	Investigational (Ph 2)		Selleck	1000
Milciclib	CDK2 inhibitor	B. Kinase inhibitor	Investigational (Ph 2)		Selleck	100
MK-2206	AKT inhibitor	B. Kinase inhibitor	Investigational (Ph 2)		ChemieTek	100
MK-2206	AKT inhibitor	B. Kinase inhibitor	Investigational (Ph 2)		ChemieTek	10
MK-8745	Aurora A inhibitor	B. Kinase inhibitor	Probe		Selleck	1000
MK-8745	Aurora A inhibitor	B. Kinase inhibitor	Probe		Selleck	100
MK-8776	CHEK1 inhibitor	B. Kinase inhibitor	Investigational (Ph 1)		Selleck	250
MK-8776	CHEK1 inhibitor	B. Kinase inhibitor	Investigational (Ph 1)		Selleck	25
Momelotinib	JAK1 & 2 inhibitor	B. Kinase inhibitor	Investigational (Ph 2)	CT-CYT387	ChemieTek	1000
Momelotinib	JAK1 & 2 inhibitor	B. Kinase inhibitor	Investigational (Ph 2)	CT-CYT387	ChemieTek	100
Motesanib	VEGFR, PDGFR, Ret, Kit inhibitor	B. Kinase inhibitor	Investigational (Ph 2)		Selleck	1000
Motesanib	VEGFR, PDGFR, Ret, Kit inhibitor	B. Kinase inhibitor	Investigational (Ph 2)		Selleck	100
Mubritinib	HER2 inhibitor	B. Kinase inhibitor	Investigational (Ph 1)		Selleck	100
Mubritinib	HER2 inhibitor	B. Kinase inhibitor	Investigational (Ph 1)		Selleck	10
Neflamapimod	p38MAPK inhibitor	B. Kinase inhibitor	Investigational (Ph 2)		Tocris Biosciences	1000
Neflamapimod	p38MAPK inhibitor	B. Kinase inhibitor	Investigational (Ph 2)		Tocris Biosciences	100
Neratinib	HER2, EGFR inhibitor	B. Kinase inhibitor	Approved (US)		Selleck	100
Neratinib	HER2, EGFR inhibitor	B. Kinase inhibitor	Approved (US)		Selleck	10
Nilotinib	Abl inhibitor	B. Kinase inhibitor	Approved	N-8207	LC Laboratories	1000
Nilotinib	Abl inhibitor	B. Kinase inhibitor	Approved	N-8207	LC Laboratories	100
Nintedanib	VEGFR, PDGFR, FGFR inhibitor	B. Kinase inhibitor	Approved	S1010-2	Selleck	1000
Nintedanib	VEGFR, PDGFR, FGFR inhibitor	B. Kinase inhibitor	Approved	S1010-2	Selleck	100
NVP-AEW541	IGF1R inhibitor	B. Kinase inhibitor	Investigational (Ph 1)		Selleck	1000
NVP-AEW541	IGF1R inhibitor	B. Kinase inhibitor	Investigational (Ph 1)		Selleck	100
NVP-BGT226	PI3K/mTOR inhibitor	B. Kinase inhibitor	Investigational (Ph 2)		Selleck	100
NVP-BGT226	PI3K/mTOR inhibitor	B. Kinase inhibitor	Investigational (Ph 2)		Selleck	10
NVP-BHG712	EphB4 inhibitor	B. Kinase inhibitor	Probe		Medchem Express	1000
NVP-BHG712	EphB4 inhibitor	B. Kinase inhibitor	Probe		Medchem Express	100
NVP-RAF265	"C-Raf" inhibitor, unclear MoA	B. Kinase inhibitor	Investigational (Ph 2)		Selleck	100
NVP-RAF265	"C-Raf" inhibitor, unclear MoA	B. Kinase inhibitor	Investigational (Ph 2)		Selleck	10

Olmutinib	EGFR(L858R/T790M) inhibitor	B. Kinase inhibitor	Investigational (Ph 2)		ChemieTek	100
Olmutinib	EGFR(L858R/T790M) inhibitor	B. Kinase inhibitor	Investigational (Ph 2)		ChemieTek	10
Omipalisib	PI3K/mTOR inhibitor	B. Kinase inhibitor	Investigational (Ph 1)		Medchem Express	100
Omipalisib	PI3K/mTOR inhibitor	B. Kinase inhibitor	Investigational (Ph 1)		Medchem Express	10
Osimertinib	EGFR(L858R/T790M) inhibitor	B. Kinase inhibitor	Approved	S7297	Selleck	250
Osimertinib	EGFR(L858R/T790M) inhibitor	B. Kinase inhibitor	Approved	S7297	Selleck	25
OSU-03012	PDPK1 inhibitor	B. Kinase inhibitor	Investigational (Ph 1)		Selleck	2500
OSU-03012	PDPK1 inhibitor	B. Kinase inhibitor	Investigational (Ph 1)		Selleck	250
OTS-964	TOPK inhibitor	B. Kinase inhibitor	Probe		ChemieTek	250
OTS-964	TOPK inhibitor	B. Kinase inhibitor	Probe		ChemieTek	25
OTS167	MELK inhibitor	B. Kinase inhibitor	Investigational (Ph 2)		Selleck	100
OTS167	MELK inhibitor	B. Kinase inhibitor	Investigational (Ph 2)		Selleck	10
Pacritinib	FLT3/JAK2	B. Kinase inhibitor	Investigational (Ph 3)		Selleck	1000
Pacritinib	FLT3/JAK2	B. Kinase inhibitor	Investigational (Ph 3)		Selleck	100
Palbociclib	CDK4/6 inhibitor	B. Kinase inhibitor	Approved	S1116-2	Selleck	10000
Palbociclib	CDK4/6 inhibitor	B. Kinase inhibitor	Approved		Selleck	1000
Palomid-529	AKT, MTOR, PI3K inhibitor	B. Kinase inhibitor	Investigational (Ph 1)		Selleck	1000
Palomid-529	AKT, MTOR, PI3K inhibitor	B. Kinase inhibitor	Investigational (Ph 1)		Selleck	100
Pazopanib	VEGFR inhibitor	B. Kinase inhibitor	Approved	P-6706	LC Laboratories	1000
Pazopanib	VEGFR inhibitor	B. Kinase inhibitor	Approved	P-6706	LC Laboratories	100
PD0325901	MEK1/2 inhibitor	B. Kinase inhibitor	Investigational (Ph 2)		Selleck	100
PD0325901	MEK1/2 inhibitor	B. Kinase inhibitor	Investigational (Ph 2)		Selleck	10
Peficitinb	JAK3-selective inhibitor	B. Kinase inhibitor	Investigational (Ph 3)		Selleck	250
Peficitinb	JAK3-selective inhibitor	B. Kinase inhibitor	Investigational (Ph 3)		Selleck	25
Perifosine	AKT/PI3K inhibitor	B. Kinase inhibitor	Investigational (Ph 3)	S1037	Selleck	10000
Perifosine	AKT/PI3K inhibitor	B. Kinase inhibitor	Investigational (Ph 3)		Selleck	1000
Pexidartinib	KIT, CSF1R, FLT3 inhibitor	B. Kinase inhibitor	Investigational (Ph 3)		Medchem Express	1000
Pexidartinib	KIT, CSF1R, FLT3 inhibitor	B. Kinase inhibitor	Investigational (Ph 3)		Medchem Express	100
PF-00477736	Chk1 inhibitor	B. Kinase inhibitor	Investigational (Ph 1)		Axon Medchem	1000
PF-00477736	Chk1 inhibitor	B. Kinase inhibitor	Investigational (Ph 1)		Axon Medchem	100
PF-00562271	FAK inhibitor	B. Kinase inhibitor	Investigational (Ph 1)		Selleck	1000
PF-00562271	FAK inhibitor	B. Kinase inhibitor	Investigational (Ph 1)		Selleck	100
PF-03758309	PAK inhibitor	B. Kinase inhibitor	Investigational (Ph 1)		ChemieTek	1000
PF-03758309	PAK inhibitor	B. Kinase inhibitor	Investigational (Ph 1)		ChemieTek	100
PF-04708671	p70S6K inhibitor	B. Kinase inhibitor	Probe		Sigma-Aldrich	1000
PF-04708671	p70S6K inhibitor	B. Kinase inhibitor	Probe		Sigma-Aldrich	100
PF-06463922	ALK, ROS1 inhibitor	B. Kinase inhibitor	Investigational (Ph 2)		Selleck	100
PF-06463922	ALK, ROS1 inhibitor	B. Kinase inhibitor	Investigational (Ph 2)		Selleck	10
PF-4800567	CK1epsilon inhibitor	B. Kinase inhibitor	Probe		Tocris Biosciences	1000
PF-4800567	CK1epsilon inhibitor	B. Kinase inhibitor	Probe		Tocris Biosciences	100
PF-670462	CK1epsilon and CK1delta inhibitor	B. Kinase inhibitor	Probe		Tocris Biosciences	1000
PF-670462	CK1epsilon and CK1delta inhibitor	B. Kinase inhibitor	Probe		Tocris Biosciences	100
PF06650833	IRAK4 inhibitor	B. Kinase inhibitor	Investigational (Ph 2)		Medchem Express	1000
PF06650833	IRAK4 inhibitor	B. Kinase inhibitor	Investigational (Ph 2)		Medchem Express	100
PH-797804	p38MAPK inhibitor	B. Kinase inhibitor	Investigational (Ph 2)		Selleck	100
PH-797804	p38MAPK inhibitor	B. Kinase inhibitor	Investigational (Ph 2)		Selleck	10
PHA 408	IKK-2 inhibitor	B. Kinase inhibitor	Probe		Axon Medchem	1000
PHA 408	IKK-2 inhibitor	B. Kinase inhibitor	Probe		Axon Medchem	100
Pictilisib	PI3K inhibitor, pan-class I	B. Kinase inhibitor	Investigational (Ph 2)	HY-50094	Medchem Express	1000
Pictilisib	PI3K inhibitor, pan-class I	B. Kinase inhibitor	Investigational (Ph 2)	HY-50094	Medchem Express	100
PIM-447	PIM1, 2, 3 kinase inhibitor	B. Kinase inhibitor	Investigational (Ph 1)		Medchem Express	1000
PIM-447	PIM1, 2, 3 kinase inhibitor	B. Kinase inhibitor	Investigational (Ph 1)		Medchem Express	100
Ponatinib	Broad TK inhibitor	B. Kinase inhibitor	Approved		Selleck	100

Ponatinib	Broad TK inhibitor	B. Kinase inhibitor	Approved	S1490	Selleck	10
Poziotinib	pan-HER inhibitor	B. Kinase inhibitor	Investigational (Ph 2)		Medchem Express	100
Poziotinib	pan-HER inhibitor	B. Kinase inhibitor	Investigational (Ph 2)		Medchem Express	10
Prexasertib	Chk1 inhibitor	B. Kinase inhibitor	Investigational (Ph 2)		Medchem Express	1000
Prexasertib	Chk1 inhibitor	B. Kinase inhibitor	Investigational (Ph 2)		Medchem Express	100
PS-1145	IKK-2 inhibitor	B. Kinase inhibitor	Probe		Axon Medchem	2500
PS-1145	IKK-2 inhibitor	B. Kinase inhibitor	Probe		Axon Medchem	250
Quizartinib	FLT3 inhibitor	B. Kinase inhibitor	Investigational (Ph 3)	CT-AC220	ChemieTek	100
Quizartinib	FLT3 inhibitor	B. Kinase inhibitor	Investigational (Ph 3)	CT-AC220	ChemieTek	10
Rabusertib	Chk1 inhibitor	B. Kinase inhibitor	Investigational (Ph 2)		Selleck	100
Rabusertib	Chk1 inhibitor	B. Kinase inhibitor	Investigational (Ph 2)		Selleck	10
Radotinib	ABL, PDGFR inhibitor	B. Kinase inhibitor	Investigational (Ph 3)		MedKoo Biosciences	1000
Radotinib	ABL, PDGFR inhibitor	B. Kinase inhibitor	Investigational (Ph 3)		MedKoo Biosciences	100
Ralimetinib	p38MAPK inhibitor	B. Kinase inhibitor	Investigational (Ph 2)		Selleck	1000
Ralimetinib	p38MAPK inhibitor	B. Kinase inhibitor	Investigational (Ph 2)		Selleck	100
Ravoxertinib	ERK inhibitor	B. Kinase inhibitor	Investigational (Ph 1)		Medchem Express	1000
Ravoxertinib	ERK inhibitor	B. Kinase inhibitor	Investigational (Ph 1)		Medchem Express	100
Regorafenib	B-Raf, c-Kit, VEGFR2 inhibitor	B. Kinase inhibitor	Approved	HY-10331	Medchem Express	1000
Regorafenib	B-Raf, c-Kit, VEGFR2 inhibitor	B. Kinase inhibitor	Approved	HY-10331	Medchem Express	100
Ribociclib	CDK4/6 inhibitor	B. Kinase inhibitor	Approved (US)		Selleck	1000
Ribociclib	CDK4/6 inhibitor	B. Kinase inhibitor	Approved (US)		Selleck	100
Ripasudil	ROCK inhibitor	B. Kinase inhibitor	Approved (Japan)	S7995	Selleck	1000
Ripasudil	ROCK inhibitor	B. Kinase inhibitor	Approved (Japan)	S7995	Selleck	100
RO5126766	dual RAF/MEK inhibitor	B. Kinase inhibitor	Investigational (Ph 1)		Medchem Express	100
RO5126766	dual RAF/MEK inhibitor	B. Kinase inhibitor	Investigational (Ph 1)		Medchem Express	10
Rociletinib	EGFR(L858R/T790M) inhibitor	B. Kinase inhibitor	Investigational (Ph 3)		ChemieTek	1000
Rociletinib	EGFR(L858R/T790M) inhibitor	B. Kinase inhibitor	Investigational (Ph 3)		ChemieTek	100
Ruboxistaurin	PKCbeta inhibitor	B. Kinase inhibitor	Investigational (Ph 3)	Axon 1401-2	Axon Medchem	1000
Ruboxistaurin	PKCbeta inhibitor	B. Kinase inhibitor	Investigational (Ph 3)	Axon 1401-2	Axon Medchem	100
Ruxolitinib	JAK1&2 inhibitor	B. Kinase inhibitor	Approved	CT-INCB-2	ChemieTek	1000
Ruxolitinib	JAK1&2 inhibitor	B. Kinase inhibitor	Approved	CT-INCB-2	ChemieTek	100
Sapanisertib	mTOR inhibitor	B. Kinase inhibitor	Investigational (Ph 1)		ChemieTek	100
Sapanisertib	mTOR inhibitor	B. Kinase inhibitor	Investigational (Ph 1)		ChemieTek	10
Sapitinib	Pan-HER inhibitor	B. Kinase inhibitor	Investigational (Ph 2)		Selleck	100
Sapitinib	Pan-HER inhibitor	B. Kinase inhibitor	Investigational (Ph 2)		Selleck	10
Saracatinib	Src, Abl inhibitor	B. Kinase inhibitor	Investigational (Ph 3)	S-8906	LC Laboratories	1000
Saracatinib	Src, Abl inhibitor	B. Kinase inhibitor	Investigational (Ph 3)		LC Laboratories	100
SCH772984	ERK1 & 2 inhibitor	B. Kinase inhibitor	Probe		ChemieTek	1000
SCH772984	ERK1 & 2 inhibitor	B. Kinase inhibitor	Probe		ChemieTek	100
Seliciclib	CDK2/7/9 inhibitor	B. Kinase inhibitor	Investigational (Ph 2)		LC Laboratories	1000
Seliciclib	CDK2/7/9 inhibitor	B. Kinase inhibitor	Investigational (Ph 2)		LC Laboratories	100
Selonsertib	ASK1 inhibitor	B. Kinase inhibitor	Investigational (Ph 2)		ChemieTek	100
Selonsertib	ASK1 inhibitor	B. Kinase inhibitor	Investigational (Ph 2)		ChemieTek	10
Selumetinib	MEK1/2 inhibitor	B. Kinase inhibitor	Investigational (Ph 3)	HY-50706	Medchem Express	1000
Selumetinib	MEK1/2 inhibitor	B. Kinase inhibitor	Investigational (Ph 3)	HY-50706	Medchem Express	100
Senexin B	CDK8/19 inhibitor	B. Kinase inhibitor	Probe		ApexBio	100
Senexin B	CDK8/19 inhibitor	B. Kinase inhibitor	Probe		ApexBio	10
Serabelisib	PI3Kalpha selective inhibitor	B. Kinase inhibitor	Investigational (Ph 2)		Medchem Express	1000
Serabelisib	PI3Kalpha selective inhibitor	B. Kinase inhibitor	Investigational (Ph 2)		Medchem Express	100
SGI-1776	PIM kinase inhibitor	B. Kinase inhibitor	Investigational (Ph 1)		Selleck	1000
SGI-1776	PIM kinase inhibitor	B. Kinase inhibitor	Investigational (Ph 1)		Selleck	100
Silmitasertib	CSNK2A1 inhibitor	B. Kinase inhibitor	Investigational (Ph 2)		Selleck	1000
Silmitasertib	CSNK2A1 inhibitor	B. Kinase inhibitor	Investigational (Ph 2)		Selleck	100

Sitravatinib	RET, TRK, PDGFR, VEGFR, KIT, DDR... inhibitor	B. Kinase inhibitor	Investigational (Ph 1)		ChemieTek	250
Sitravatinib	RET, TRK, PDGFR, VEGFR, KIT, DDR... inhibitor	B. Kinase inhibitor	Investigational (Ph 1)		ChemieTek	25
SNS-032	CDK inhibitor	B. Kinase inhibitor	Investigational (Ph 2)		Medchem Express	1000
SNS-032	CDK inhibitor	B. Kinase inhibitor	Investigational (Ph 2)		Medchem Express	100
Sonolisib	PI3K inhibitor, pan-class I. Irreversible	B. Kinase inhibitor	Investigational (Ph 2)		Active Biochem	1000
Sonolisib	PI3K inhibitor, pan-class I. Irreversible	B. Kinase inhibitor	Investigational (Ph 2)		Active Biochem	100
Sorafenib	B-Raf, FGFR-1, VEGFR-2 & -3, PDGFR-beta, KIT, and FLT3 inhib	B. Kinase inhibitor	Approved	S-8502	LC Laboratories	100
Sorafenib	B-Raf, FGFR-1, VEGFR-2 & -3, PDGFR-beta, KIT, and FLT3 inhib	B. Kinase inhibitor	Approved	S-8502	LC Laboratories	10
Sotrastaurin	PKC inhibitor	B. Kinase inhibitor	Investigational (Ph 2)		Axon Medchem	1000
Sotrastaurin	PKC inhibitor	B. Kinase inhibitor	Investigational (Ph 2)		Axon Medchem	100
Spebrutinib	BTK inhibitor	B. Kinase inhibitor	Investigational (Ph 2)		Medchem Express	100
Spebrutinib	BTK inhibitor	B. Kinase inhibitor	Investigational (Ph 2)		Medchem Express	10
Sunitinib	Broad TK inhibitor	B. Kinase inhibitor	Approved	S-8803	LC Laboratories	100
Sunitinib	Broad TK inhibitor	B. Kinase inhibitor	Approved	S-8803	LC Laboratories	10
TAK-285	HER2 inhibitor	B. Kinase inhibitor	Investigational (Ph 1)		Selleck	250
TAK-285	HER2 inhibitor	B. Kinase inhibitor	Investigational (Ph 1)		Selleck	25
TAK-530	pan-RAF inhibitor	B. Kinase inhibitor	Investigational (Ph 1)		Medchem Express	1000
TAK-530	pan-RAF inhibitor	B. Kinase inhibitor	Investigational (Ph 1)		Medchem Express	100
TAK-901	Aurora, Src family, JAK3, RTK inhibitor	B. Kinase inhibitor	Investigational (Ph 1)		Selleck	100
TAK-901	Aurora, Src family, JAK3, RTK inhibitor	B. Kinase inhibitor	Investigational (Ph 1)		Selleck	10
Talmapimod	p38MAPK alpha selective inhibitor	B. Kinase inhibitor	Investigational (Ph 2)		Axon Medchem	1000
Talmapimod	p38MAPK alpha selective inhibitor	B. Kinase inhibitor	Investigational (Ph 2)		Axon Medchem	100
Tamatinib	Syk inhibitor	B. Kinase inhibitor	Investigational (Ph 1)		Selleck	1000
Tamatinib	Syk inhibitor	B. Kinase inhibitor	Investigational (Ph 1)		Selleck	100
Tandutinib	FLT3, PDGFR, KIT inhibitor	B. Kinase inhibitor	Investigational (Ph 2)		LC Laboratories	100
Tandutinib	FLT3, PDGFR, KIT inhibitor	B. Kinase inhibitor	Investigational (Ph 2)		LC Laboratories	10
Tanzisertib	JNK inhibitor	B. Kinase inhibitor	Investigational (Ph 2)		Medchem Express	1000
Tanzisertib	JNK inhibitor	B. Kinase inhibitor	Investigational (Ph 2)		Medchem Express	100
Taselisib	PI3K alpha, delta, (gamma) selective inhibitor	B. Kinase inhibitor	Investigational (Ph 3)		Medchem Express	100
Taselisib	PI3K alpha, delta, (gamma) selective inhibitor	B. Kinase inhibitor	Investigational (Ph 3)		Medchem Express	10
Telatinib	VEGFR, KIT, PDGFR inhibitor	B. Kinase inhibitor	Investigational (Ph 2)		Selleck	1000
Telatinib	VEGFR, KIT, PDGFR inhibitor	B. Kinase inhibitor	Investigational (Ph 2)		Selleck	100
Tepotinib	c-Met inhibitor	B. Kinase inhibitor	Investigational (Ph 1)		ChemieTek	100
Tepotinib	c-Met inhibitor	B. Kinase inhibitor	Investigational (Ph 1)		ChemieTek	10
Tesevatinib	EGFR, ERBB2, VEGFR, EPHB4	B. Kinase inhibitor	Investigational (Ph 2)		Santa Cruz Biotechnology	100
Tesevatinib	EGFR, ERBB2, VEGFR, EPHB4	B. Kinase inhibitor	Investigational (Ph 2)		Santa Cruz Biotechnology	10
TEW-7197	TGF-β receptor ALK4/ALK5 inhibitor	B. Kinase inhibitor	Investigational (Ph 1)		Selleck	250
TEW-7197	TGF-β receptor ALK4/ALK5 inhibitor	B. Kinase inhibitor	Investigational (Ph 1)		Selleck	25
TG100-115	PI3K gamma/delta inhibitor	B. Kinase inhibitor	Investigational (Ph 2)		Selleck	1000
TG100-115	PI3K gamma/delta inhibitor	B. Kinase inhibitor	Investigational (Ph 2)		Selleck	100
TGR-1202	PI3Kdelta inhibitor	B. Kinase inhibitor	Investigational (Ph 3)		Medchem Express	250
TGR-1202	PI3Kdelta inhibitor	B. Kinase inhibitor	Investigational (Ph 3)		Medchem Express	25
TGX-221	PI3K beta selective inhibitor	B. Kinase inhibitor	Probe		ChemieTek	1000
TGX-221	PI3K beta selective inhibitor	B. Kinase inhibitor	Probe		ChemieTek	100
THZ2	CDK7 inhibitor	B. Kinase inhibitor	Probe		Medchem Express	1000
THZ2	CDK7 inhibitor	B. Kinase inhibitor	Probe		Medchem Express	100
Tideglusib	GSK3 inhibitor	B. Kinase inhibitor	Investigational (Ph 2)		Selleck	300
Tideglusib	GSK3 inhibitor	B. Kinase inhibitor	Investigational (Ph 2)		Selleck	30
Tirabrutinib	BTK inhibitor	B. Kinase inhibitor	Investigational (Ph 1)		Medchem Express	100
Tirabrutinib	BTK inhibitor	B. Kinase inhibitor	Investigational (Ph 1)		Medchem Express	10
Tivantinib	MET inhibitor	B. Kinase inhibitor	Investigational (Ph 2)		ChemieTek	100
Tivantinib	MET inhibitor	B. Kinase inhibitor	Investigational (Ph 2)		ChemieTek	10
Tivozanib	VEGFR1, 2, 3, c-Kit, PDGFRB inhibitor	B. Kinase inhibitor	Investigational (Ph 3)	CT-AV951	ChemieTek	1000

Tivozanib	VEGFR1, 2, 3, c-Kit, PDGFRB inhibitor	B. Kinase inhibitor	Investigational (Ph 3)	CT-AV951	ChemieTek	100
Tofacitinib	JAK3, JAK2(V617F) inhibitor	B. Kinase inhibitor	Approved	T-1377	LC Laboratories	500
Tofacitinib	JAK3, JAK2(V617F) inhibitor	B. Kinase inhibitor	Approved	T-1377	LC Laboratories	50
Tozasertib	pan-Aurora inhibitor	B. Kinase inhibitor	Investigational (Ph 2)		Selleck	1000
Tozasertib	pan-Aurora inhibitor	B. Kinase inhibitor	Investigational (Ph 2)		Selleck	100
Trametinib	MEK1/2 inhibitor	B. Kinase inhibitor	Approved		ChemieTek	25
Trametinib	MEK1/2 inhibitor	B. Kinase inhibitor	Approved	CT-GSK112	ChemieTek	2,5
Triciribine	AKT inhibitor	B. Kinase inhibitor	Investigational (Ph 2)		Selleck	10000
Triciribine	AKT inhibitor	B. Kinase inhibitor	Investigational (Ph 2)		Selleck	1000
Tucatinib	HER2 inhibitor	B. Kinase inhibitor	Investigational (Ph 1)		Selleck	250
Tucatinib	HER2 inhibitor	B. Kinase inhibitor	Investigational (Ph 1)		Selleck	25
UCN-01	PKCbeta, PDK1, Chk, Cdk2 inhibitor	B. Kinase inhibitor	Investigational (Ph 2)		Sigma-Aldrich	1000
UCN-01	PKCbeta, PDK1, Chk, Cdk2 inhibitor	B. Kinase inhibitor	Investigational (Ph 2)		Sigma-Aldrich	100
Ulixertinib	ERK inhibitor	B. Kinase inhibitor	Investigational (Ph 2)		ChemieTek	1000
Ulixertinib	ERK inhibitor	B. Kinase inhibitor	Investigational (Ph 2)		ChemieTek	100
UNC2881	MER inhibitor	B. Kinase inhibitor	Probe		Selleck	250
UNC2881	MER inhibitor	B. Kinase inhibitor	Probe		Selleck	25
Upadacitinib	JAK1-selective inhibitor	B. Kinase inhibitor	Investigational (Ph 3)		Medchem Express	1000
Upadacitinib	JAK1-selective inhibitor	B. Kinase inhibitor	Investigational (Ph 3)		Medchem Express	100
Uprosertib	AKT inhibitor	B. Kinase inhibitor	Investigational (Ph 2)		Medchem Express	1000
Uprosertib	AKT inhibitor	B. Kinase inhibitor	Investigational (Ph 2)		Medchem Express	100
Vandetanib	VEGFR,EGFR, RET inhibitor	B. Kinase inhibitor	Approved	V-9402	LC Laboratories	100
Vandetanib	VEGFR,EGFR, RET inhibitor	B. Kinase inhibitor	Approved	V-9402	LC Laboratories	10
Varlitinib	EGFR HER2 inhibitor	B. Kinase inhibitor	Investigational (Ph 2)		Selleck	1000
Varlitinib	EGFR HER2 inhibitor	B. Kinase inhibitor	Investigational (Ph 2)		Selleck	100
Vatalanib	VEGFR-1 & -2 inhibitor	B. Kinase inhibitor	Investigational (Ph 3)	V-8303	LC Laboratories	1000
Vatalanib	VEGFR-1 & -2 inhibitor	B. Kinase inhibitor	Investigational (Ph 3)	V-8303	LC Laboratories	100
VE-821	ATR inihibitor	B. Kinase inhibitor	Probe		Selleck	1000
VE-821	ATR inihibitor	B. Kinase inhibitor	Probe		Selleck	100
Vemurafenib	B-Raf(V600E) inhibitor	B. Kinase inhibitor	Approved	CT-P4032-2	ChemieTek	1000
Vemurafenib	B-Raf(V600E) inhibitor	B. Kinase inhibitor	Approved	CT-P4032-2	ChemieTek	100
Vistusertib	mTOR inhibitor, ATP-competitive	B. Kinase inhibitor	Investigational (Ph 2)		Medchem Express	1000
Vistusertib	mTOR inhibitor, ATP-competitive	B. Kinase inhibitor	Investigational (Ph 2)		Medchem Express	100
Volasertib	PLK1 inhibitor	B. Kinase inhibitor	Investigational (Ph 3)	CT-BI6727	ChemieTek	100
Volasertib	PLK1 inhibitor	B. Kinase inhibitor	Investigational (Ph 3)		ChemieTek	10
VS-4718	FAK inhibitor	B. Kinase inhibitor	Investigational (Ph 1)		ChemieTek	1000
VS-4718	FAK inhibitor	B. Kinase inhibitor	Investigational (Ph 1)		ChemieTek	100
ZSTK474	PI3K gamma selective inhibitor	B. Kinase inhibitor	Investigational (Ph 1)		LC Laboratories	1000
ZSTK474	PI3K gamma selective inhibitor	B. Kinase inhibitor	Investigational (Ph 1)		LC Laboratories	100
Everolimus	binds FKBP12, causes inhibition of mTORC1	C. Rapalog	Approved	E-4040	LC Laboratories	10
Everolimus	binds FKBP12, causes inhibition of mTORC1	C. Rapalog	Approved	E-4040	LC Laboratories	1
Ridaforolimus	binds FKBP12, causes inhibition of mTORC1	C. Rapalog	Investigational (Ph 3)		Active Biochem	10
Ridaforolimus	binds FKBP12, causes inhibition of mTORC1	C. Rapalog	Investigational (Ph 3)		Active Biochem	1
Sirolimus	binds FKBP12, causes inhibition of mTORC1	C. Rapalog	Approved	R-5000	LC Laboratories	10
Sirolimus	binds FKBP12, causes inhibition of mTORC1	C. Rapalog	Approved		LC Laboratories	1
Tacrolimus	Binds FKBP12, causes inhibition of calcineurin	C. Rapalog	Approved	3631	Tocris Biosciences	1000
Tacrolimus	Binds FKBP12, causes inhibition of calcineurin	C. Rapalog	Approved		Tocris Biosciences	100
Temsirolimus	binds FKBP12, causes inhibition of mTORC1	C. Rapalog	Approved	T-8040	LC Laboratories	10
Temsirolimus	binds FKBP12, causes inhibition of mTORC1	C. Rapalog	Approved	T-8040	LC Laboratories	1
Bimatoprost	Prostaglandin analog	D. Immunomodulatory	Approved		Selleck	550
Bimatoprost	Prostaglandin analog	D. Immunomodulatory	Approved		Selleck	55
CC122	IMiD immunomodulator	D. Immunomodulatory	Investigational (Ph 2)		Medchem Express	100
Dexamethasone	Glucocorticoid, immunomodulatory agent	D. Immunomodulatory	Approved	S1322	Selleck	1000

Dexamethasone	Glucocorticoid, immunomodulatory agent	D. Immunomodulatory	Approved	S1322	Selleck	100
Epacadostat	IDO inhibitor	D. Immunomodulatory	Investigational (Ph 3)		Medchem Express	1000
Epacadostat	IDO inhibitor	D. Immunomodulatory	Investigational (Ph 3)		Medchem Express	100
GDC-0919	IDO inhibitor	D. Immunomodulatory	Investigational (Ph 1)		Selleck	1000
GDC-0919	IDO inhibitor	D. Immunomodulatory	Investigational (Ph 1)		Selleck	100
Imiquimod	Immunomodulatory agent, TLR7 agonist	D. Immunomodulatory	Approved	HY-B0180	Medchem Express	250
Imiquimod	Immunomodulatory agent, TLR7 agonist	D. Immunomodulatory	Approved	HY-B0180	Medchem Express	25
Lenalidomide	Immunomodulatory	D. Immunomodulatory	Approved	L-5499	LC Laboratories	10000
Lenalidomide	Immunomodulatory	D. Immunomodulatory	Approved		LC Laboratories	1000
Methylprednisolone	Glucocorticoid, immunomodulatory agent	D. Immunomodulatory	Approved	sc-205749	Santa Cruz Biotechnology	1000
Methylprednisolone	Glucocorticoid, immunomodulatory agent	D. Immunomodulatory	Approved	sc-205749	Santa Cruz Biotechnology	100
Motolimod	TLR8 agonist	D. Immunomodulatory	Investigational (Ph 2)		Medchem Express	1000
Motolimod	TLR8 agonist	D. Immunomodulatory	Investigational (Ph 2)		Medchem Express	100
Pirfenidone	Antifibrotic and anti-inflammatory	D. Immunomodulatory	Approved	CT-PIRF	ChemieTek	1000
Pirfenidone	Antifibrotic and anti-inflammatory	D. Immunomodulatory	Approved	CT-PIRF	ChemieTek	100
Pomalidomide	Immunomodulatory agent, anti-angiogenic	D. Immunomodulatory	Approved	P0018	Sigma-Aldrich	1000
Pomalidomide	Immunomodulatory agent, anti-angiogenic	D. Immunomodulatory	Approved	P0018	Sigma-Aldrich	100
Prednisolone	Glucocorticoid, immunomodulatory agent	D. Immunomodulatory	Approved	sc-205815	Santa Cruz Biotechnology	1000
Prednisolone	Glucocorticoid, immunomodulatory agent	D. Immunomodulatory	Approved	sc-205815	Santa Cruz Biotechnology	100
Resatorvid	TLR4 inhibitor	D. Immunomodulatory	Investigational (Ph 3)		Medchem Express	1000
Resatorvid	TLR4 inhibitor	D. Immunomodulatory	Investigational (Ph 3)		Medchem Express	100
Resiquimod	TLR7/TLR8 agonist	D. Immunomodulatory	Investigational (Ph 2)		Medchem Express	1000
Resiquimod	TLR7/TLR8 agonist	D. Immunomodulatory	Investigational (Ph 2)		Medchem Express	100
Tasquinimod	S100A9 inhibitor, immunomodulatory, anti-angiogenic	D. Immunomodulatory	Investigational (Ph 3)		Medchem Express	1000
Tasquinimod	S100A9 inhibitor, immunomodulatory, anti-angiogenic	D. Immunomodulatory	Investigational (Ph 3)		Medchem Express	100
Thalidomide	Immunosuppressant	D. Immunomodulatory	Approved	HY-14658	Medchem Express	1000
Thalidomide	Immunosuppressant	D. Immunomodulatory	Approved	HY-14658	Medchem Express	100
Vesatolimod	TLR7 agonist	D. Immunomodulatory	Investigational (Ph 2)		Medchem Express	1000
Vesatolimod	TLR7 agonist	D. Immunomodulatory	Investigational (Ph 2)		Medchem Express	100
VGX-1027	Nitric oxide-donating immunomodulator	D. Immunomodulatory	Investigational (Ph 1)		Selleck	1000
VGX-1027	Nitric oxide-donating immunomodulator	D. Immunomodulatory	Investigational (Ph 1)		Selleck	100
A-366	G9a/GLP inhibitor	E. Differentiating/ epigenetic modifier	Probe		Tocris Biosciences	2500
A-366	G9a/GLP inhibitor	E. Differentiating/ epigenetic modifier	Probe		Tocris Biosciences	250
Abexinostat	HDAC1-selective inhibitor	E. Differentiating/ epigenetic modifier	Investigational (Ph 2)		Medchem Express	1000
Abexinostat	HDAC1-selective inhibitor	E. Differentiating/ epigenetic modifier	Investigational (Ph 2)		Medchem Express	100
Acitretin	Retinoid receptor agonist	E. Differentiating/ epigenetic modifier	Approved (non-oncolog	HY-B0107	Medchem Express	1000
Acitretin	Retinoid receptor agonist	E. Differentiating/ epigenetic modifier	Approved (non-oncolog	HY-B0107	Medchem Express	100
AR-42	HDAC inhibitor	E. Differentiating/ epigenetic modifier	Investigational (Ph 1)		Selleck	1000
AR-42	HDAC inhibitor	E. Differentiating/ epigenetic modifier	Investigational (Ph 1)		Selleck	100
Arsenic(III) oxide	Thioredoxin reductase inhibitor; cytotoxic chemotherapeutic	E. Differentiating/ epigenetic modifier	Approved	202673	Sigma-Aldrich	10000
Arsenic(III) oxide	Thioredoxin reductase inhibitor; cytotoxic chemotherapeutic	E. Differentiating/ epigenetic modifier	Approved	202673	Sigma-Aldrich	1000
ARV-825	BET-targeting PROTAC	E. Differentiating/ epigenetic modifier	Probe		ChemieTek	30
ARV-825	BET-targeting PROTAC	E. Differentiating/ epigenetic modifier	Probe		ChemieTek	3
Azacitidine	Nucleoside analog DNA methyl transferase inhibitor	E. Differentiating/ epigenetic modifier	Approved	HY-10586	Medchem Express	1000
Azacitidine	Nucleoside analog DNA methyl transferase inhibitor	E. Differentiating/ epigenetic modifier	Approved	HY-10586	Medchem Express	100
BAY 87-2243	HIF1alpha inhibitor	E. Differentiating/ epigenetic modifier	Investigational (Ph 1)		Selleck	100
BAY 87-2243	HIF1alpha inhibitor	E. Differentiating/ epigenetic modifier	Investigational (Ph 1)		Selleck	10
BAY-1436032	IDH1 R132H/R132C inhibitor	E. Differentiating/ epigenetic modifier	Investigational (Ph 1)		Active Biochem	1000
BAY-1436032	IDH1 R132H/R132C inhibitor	E. Differentiating/ epigenetic modifier	Investigational (Ph 1)		Active Biochem	100
Belinostat	HDAC inhibitor	E. Differentiating/ epigenetic modifier	Approved (US)	CT-BELI	ChemieTek	1000
Belinostat	HDAC inhibitor	E. Differentiating/ epigenetic modifier	Approved (US)	CT-BELI	ChemieTek	100
Bexarotene	Antineoplastic agent; retinoid specifically selective for retinoid X recep	E. Differentiating/ epigenetic modifier	Approved	sc-217753	Santa Cruz Biotechnology	1000
Bexarotene	Antineoplastic agent; retinoid specifically selective for retinoid X recep	E. Differentiating/ epigenetic modifier	Approved	sc-217753	Santa Cruz Biotechnology	100

Birabresib	BET family inhibitor	E. Differentiating/ epigenetic modifier	Investigational (Ph 2)		Selleck	1000
Birabresib	BET family inhibitor	E. Differentiating/ epigenetic modifier	Investigational (Ph 2)		Selleck	100
C646	p300/CREB-binding protein (CBP) inhibitor	E. Differentiating/ epigenetic modifier	Probe		Axon Medchem	2500
CPI-0610	BET family inhibitor	E. Differentiating/ epigenetic modifier	Investigational (Ph 1)		ChemieTek	1000
CPI-0610	BET family inhibitor	E. Differentiating/ epigenetic modifier	Investigational (Ph 1)		ChemieTek	100
CPI-360	EZH2 inhibitor	E. Differentiating/ epigenetic modifier	Probe		Medchem Express	1000
CPI-360	EZH2 inhibitor	E. Differentiating/ epigenetic modifier	Probe		Medchem Express	100
CUDC-907	HDAC1/2/3/10, PI3Kalpha inhibitor	E. Differentiating/ epigenetic modifier	Investigational (Ph 2)		Selleck	1000
CUDC-907	HDAC1/2/3/10, PI3Kalpha inhibitor	E. Differentiating/ epigenetic modifier	Investigational (Ph 2)		Selleck	100
dBET1	BET-targeting PROTAC	E. Differentiating/ epigenetic modifier	Probe		ChemieTek	1000
dBET1	BET-targeting PROTAC	E. Differentiating/ epigenetic modifier	Probe		ChemieTek	100
Decitabine	Nucleoside analog DNA methyl transferase inhibitor	E. Differentiating/ epigenetic modifier	Approved	S1200	Selleck	1000
Decitabine	Nucleoside analog DNA methyl transferase inhibitor	E. Differentiating/ epigenetic modifier	Approved		Selleck	100
Enasidenib	IDH2-R140Q inhibitor	E. Differentiating/ epigenetic modifier	Approved (US)		Medchem Express	1000
Enasidenib	IDH2-R140Q inhibitor	E. Differentiating/ epigenetic modifier	Approved (US)		Medchem Express	100
Entinostat	HDAC inhibitor	E. Differentiating/ epigenetic modifier	Investigational (Ph 2)		LC Laboratories	100
EPZ-5687	EZH2 inhibitor	E. Differentiating/ epigenetic modifier	Probe		ChemieTek	1000
EPZ-5687	EZH2 inhibitor	E. Differentiating/ epigenetic modifier	Probe		ChemieTek	100
EPZ015666	PRMT5 inhibitor	E. Differentiating/ epigenetic modifier	Probe		Selleck	1000
EPZ015666	PRMT5 inhibitor	E. Differentiating/ epigenetic modifier	Probe		Selleck	100
EPZ031686	SMYD3 inhibitor	E. Differentiating/ epigenetic modifier	Probe		Medchem Express	1000
EPZ031686	SMYD3 inhibitor	E. Differentiating/ epigenetic modifier	Probe		Medchem Express	100
Givinostat	HDAC inhibitor	E. Differentiating/ epigenetic modifier	Investigational (Ph 2)		Selleck	100
Givinostat	HDAC inhibitor	E. Differentiating/ epigenetic modifier	Investigational (Ph 2)		Selleck	10
GSK-J4	JMJD3 (histone demethylase) inhibitor	E. Differentiating/ epigenetic modifier	Probe		Medchem Express	10000
GSK-J4	JMJD3 (histone demethylase) inhibitor	E. Differentiating/ epigenetic modifier	Probe		Medchem Express	1000
GSK2801	BAZ2B/A bromodomain inhibitor	E. Differentiating/ epigenetic modifier	Probe		Sigma-Aldrich	1000
GSK2801	BAZ2B/A bromodomain inhibitor	E. Differentiating/ epigenetic modifier	Probe		Sigma-Aldrich	100
GSK2879552	LSD1 inhibitor	E. Differentiating/ epigenetic modifier	Investigational (Ph 1)		ChemieTek	1000
GSK343	EZH2 inhibitor	E. Differentiating/ epigenetic modifier	Probe		Medchem Express	100
GSK343	EZH2 inhibitor	E. Differentiating/ epigenetic modifier	Probe		Medchem Express	10
I-BET151	BET family inhibitor	E. Differentiating/ epigenetic modifier	Probe		ChemieTek	1000
I-BET151	BET family inhibitor	E. Differentiating/ epigenetic modifier	Probe		ChemieTek	100
IOX-1	2-Oxoglutarate Oxygenase Inhibitor	E. Differentiating/ epigenetic modifier	Probe		Selleck	10000
IOX-1	2-Oxoglutarate Oxygenase Inhibitor	E. Differentiating/ epigenetic modifier	Probe		Selleck	1000
IOX-2	PHD2 inhibitor	E. Differentiating/ epigenetic modifier	Probe		Tocris Biosciences	5000
IOX-2	PHD2 inhibitor	E. Differentiating/ epigenetic modifier	Probe		Tocris Biosciences	500
Ivosidenib	IDH1 R132H/R132C inhibitor	E. Differentiating/ epigenetic modifier	Investigational (Ph 3)		ChemieTek	1000
Ivosidenib	IDH1 R132H/R132C inhibitor	E. Differentiating/ epigenetic modifier	Investigational (Ph 3)		ChemieTek	100
JQ1	BET family inhibitor	E. Differentiating/ epigenetic modifier	Probe		Medchem Express	1000
JQ1	BET family inhibitor	E. Differentiating/ epigenetic modifier	Probe		Medchem Express	100
Lomeguatrib	O6-methylguanine-DNA methyltransferase inhibitor	E. Differentiating/ epigenetic modifier	Investigational (Ph 2)		Tocris Biosciences	1000
Lomeguatrib	O6-methylguanine-DNA methyltransferase inhibitor	E. Differentiating/ epigenetic modifier	Investigational (Ph 2)		Tocris Biosciences	100
Lonafarnib	Farnesyl transferase inhibitor	E. Differentiating/ epigenetic modifier	Investigational (Ph 3)		Selleck	10000
Lonafarnib	Farnesyl transferase inhibitor	E. Differentiating/ epigenetic modifier	Investigational (Ph 3)		Selleck	1000
Mivebresib	BET family inhibitor	E. Differentiating/ epigenetic modifier	Investigational (Ph 1)		Medchem Express	1000
Mivebresib	BET family inhibitor	E. Differentiating/ epigenetic modifier	Investigational (Ph 1)		Medchem Express	100
ML390	DHODH inhibitor	E. Differentiating/ epigenetic modifier	Probe		Medchem Express	5000
ML390	DHODH inhibitor	E. Differentiating/ epigenetic modifier	Probe		Medchem Express	500
Mocetinostat	HDAC inhibitor (HDAC1 & 2-selective)	E. Differentiating/ epigenetic modifier	Investigational (Ph 2)		Selleck	1000
Mocetinostat	HDAC inhibitor (HDAC1 & 2-selective)	E. Differentiating/ epigenetic modifier	Investigational (Ph 2)		Selleck	100
Molibresib	BET family inhibitor	E. Differentiating/ epigenetic modifier	Investigational (Ph 1)		ChemieTek	1000
Molibresib	BET family inhibitor	E. Differentiating/ epigenetic modifier	Investigational (Ph 1)		ChemieTek	100

Niraparib	PARP inhibitor	E. Differentiating/ epigenetic modifier	Approved (US)		ChemieTek	1000
Niraparib	PARP inhibitor	E. Differentiating/ epigenetic modifier	Approved (US)		ChemieTek	100
Olaparib	PARP inhibitor	E. Differentiating/ epigenetic modifier	Approved	O-9201	LC Laboratories	1000
Olaparib	PARP inhibitor	E. Differentiating/ epigenetic modifier	Approved	O-9201	LC Laboratories	100
Panobinostat	HDAC inhibitor	E. Differentiating/ epigenetic modifier	Approved	P-3703	LC Laboratories	100
Panobinostat	HDAC inhibitor	E. Differentiating/ epigenetic modifier	Approved	P-3703	LC Laboratories	10
PCI-34051	HDAC8 inhibitor	E. Differentiating/ epigenetic modifier	Probe		Medchem Express	1000
PCI-34051	HDAC8 inhibitor	E. Differentiating/ epigenetic modifier	Probe		Medchem Express	100
PFI-1	BET family inhibitor	E. Differentiating/ epigenetic modifier	Probe		Medchem Express	3000
PFI-1	BET family inhibitor	E. Differentiating/ epigenetic modifier	Probe		Medchem Express	300
Pinometostat	DOT1L inhibitor	E. Differentiating/ epigenetic modifier	Investigational (Ph 1)		Selleck	100
Pinometostat	DOT1L inhibitor	E. Differentiating/ epigenetic modifier	Investigational (Ph 1)		Selleck	10
Pracinostat	HDAC inhibitor	E. Differentiating/ epigenetic modifier	Investigational (Ph 2)		Selleck	1000
Pracinostat	HDAC inhibitor	E. Differentiating/ epigenetic modifier	Investigational (Ph 2)		Selleck	100
PTC-209	BMI-1 inhibitor	E. Differentiating/ epigenetic modifier	Probe		Selleck	1000
PTC-209	BMI-1 inhibitor	E. Differentiating/ epigenetic modifier	Probe		Selleck	100
Quisinostat	HDAC inhibitor	E. Differentiating/ epigenetic modifier	Investigational (Ph 2)	A-1162	Active Biochem	100
Quisinostat	HDAC inhibitor	E. Differentiating/ epigenetic modifier	Investigational (Ph 2)	A-1162	Active Biochem	10
Resminostat	HDAC1, 3, 6 inhibitor	E. Differentiating/ epigenetic modifier	Investigational (Ph 2)		Medchem Express	1000
Resminostat	HDAC1, 3, 6 inhibitor	E. Differentiating/ epigenetic modifier	Investigational (Ph 2)		Medchem Express	100
RGFP966	HDAC3 inhibitor	E. Differentiating/ epigenetic modifier	Probe		Selleck	1000
RGFP966	HDAC3 inhibitor	E. Differentiating/ epigenetic modifier	Probe		Selleck	100
Rocilinostat	HDAC-6 selective inhibitor	E. Differentiating/ epigenetic modifier	Investigational (Ph 1)		ChemieTek	1000
Rocilinostat	HDAC-6 selective inhibitor	E. Differentiating/ epigenetic modifier	Investigational (Ph 1)		ChemieTek	100
Romidepsin	HDAC inhibitor	E. Differentiating/ epigenetic modifier	Approved	HY-15149	Medchem Express	100
Romidepsin	HDAC inhibitor	E. Differentiating/ epigenetic modifier	Approved	HY-15149	Medchem Express	10
Roxadustat	HIF prolyl hydroxylase inhibitor	E. Differentiating/ epigenetic modifier	Investigational (Ph 2)		Medchem Express	1000
Roxadustat	HIF prolyl hydroxylase inhibitor	E. Differentiating/ epigenetic modifier	Investigational (Ph 2)		Medchem Express	100
Rucaparib	PARP inhibitor	E. Differentiating/ epigenetic modifier	Approved (US)	S1098	Selleck	1000
Rucaparib	PARP inhibitor	E. Differentiating/ epigenetic modifier	Approved (US)	S1098	Selleck	100
SGC-CBP30	CREBBP/EP300 bromodomain inhibitor	E. Differentiating/ epigenetic modifier	Probe		Medchem Express	2500
SGC-CBP30	CREBBP/EP300 bromodomain inhibitor	E. Differentiating/ epigenetic modifier	Probe		Medchem Express	250
SGC0946	DOT1L inhibitor	E. Differentiating/ epigenetic modifier	Probe		Selleck	1000
SGC0946	DOT1L inhibitor	E. Differentiating/ epigenetic modifier	Probe		Selleck	100
StemRegenin 1	AHR antagonist, stem cell regenerating	E. Differentiating/ epigenetic modifier	Probe		ChemieTek	1000
StemRegenin 1	AHR antagonist, stem cell regenerating	E. Differentiating/ epigenetic modifier	Probe		ChemieTek	100
Tacedinaline	HDAC inhibitor	E. Differentiating/ epigenetic modifier	Investigational (Ph 3)		LC Laboratories	10
Talazoparib	PARP1/2 inhibitor	E. Differentiating/ epigenetic modifier	Investigational (Ph 3)		Medchem Express	100
Talazoparib	PARP1/2 inhibitor	E. Differentiating/ epigenetic modifier	Investigational (Ph 3)		Medchem Express	10
Tazemetostat	EZH2 inhibitor	E. Differentiating/ epigenetic modifier	Investigational (Ph 2)		ChemieTek	1000
Tazemetostat	EZH2 inhibitor	E. Differentiating/ epigenetic modifier	Investigational (Ph 2)		ChemieTek	100
Tipifarnib	Farnesyltransferase inhibitor	E. Differentiating/ epigenetic modifier	Investigational (Ph 3)		Selleck	1000
Tipifarnib	Farnesyltransferase inhibitor	E. Differentiating/ epigenetic modifier	Investigational (Ph 3)		Selleck	100
Tretinoin	Retinoic acid receptor agonist	E. Differentiating/ epigenetic modifier	Approved	HY-14649	Medchem Express	1000
Tretinoin	Retinoic acid receptor agonist	E. Differentiating/ epigenetic modifier	Approved	HY-14649	Medchem Express	100
Tubacin	HDAC6 inhibitor	E. Differentiating/ epigenetic modifier	Probe		Selleck	1000
Tubacin	HDAC6 inhibitor	E. Differentiating/ epigenetic modifier	Probe		Selleck	100
Tubastatin A	HDAC6 inhibitor	E. Differentiating/ epigenetic modifier	Probe		ChemieTek	1000
Tubastatin A	HDAC6 inhibitor	E. Differentiating/ epigenetic modifier	Probe		ChemieTek	100
Tucidinostat	HDAC1/2/3/10 inhibitor	E. Differentiating/ epigenetic modifier	Investigational (Ph 2)		Medchem Express	1000
Tucidinostat	HDAC1/2/3/10 inhibitor	E. Differentiating/ epigenetic modifier	Investigational (Ph 2)		Medchem Express	100
UM729	Enhancer of aryl hydrocarbon receptor (AhR) antagonists	E. Differentiating/ epigenetic modifier	Probe		Medchem Express	1000
UM729	Enhancer of aryl hydrocarbon receptor (AhR) antagonists	E. Differentiating/ epigenetic modifier	Probe		Medchem Express	100

UNC0638	G9a/GLP inhibitor	E. Differentiating/ epigenetic modifier	Probe		Tocris Biosciences	1000
UNC0638	G9a/GLP inhibitor	E. Differentiating/ epigenetic modifier	Probe		Tocris Biosciences	100
UNC0642	G9a/GLP inhibitor	E. Differentiating/ epigenetic modifier	Probe	5132	Tocris Biosciences	1000
UNC0642	G9a/GLP inhibitor	E. Differentiating/ epigenetic modifier	Probe	5132	Tocris Biosciences	100
UNC1215	L3MBTL3 inhibitor	E. Differentiating/ epigenetic modifier	Probe		Medchem Express	1000
UNC1215	L3MBTL3 inhibitor	E. Differentiating/ epigenetic modifier	Probe		Medchem Express	100
Valproic acid	HDAC inhibitor	E. Differentiating/ epigenetic modifier	Approved		Sigma-Aldrich	10000
Valproic acid	HDAC inhibitor	E. Differentiating/ epigenetic modifier	Approved	P4543	Sigma-Aldrich	1000
Veliparib	PARP inhibitor	E. Differentiating/ epigenetic modifier	Investigational (Ph 3)		Selleck	1000
Veliparib	PARP inhibitor	E. Differentiating/ epigenetic modifier	Investigational (Ph 3)		Selleck	100
Vidofludimus	DHODH inhibitor	E. Differentiating/ epigenetic modifier	Investigational (Ph 2)		Medchem Express	1000
Vidofludimus	DHODH inhibitor	E. Differentiating/ epigenetic modifier	Investigational (Ph 2)		Medchem Express	100
Vorinostat	HDAC inhibitor	E. Differentiating/ epigenetic modifier	Approved	V-8477	LC Laboratories	1000
Vorinostat	HDAC inhibitor	E. Differentiating/ epigenetic modifier	Approved	V-8477	LC Laboratories	100
XAV-939	Tankyrase-1 and -2	E. Differentiating/ epigenetic modifier	Probe		Selleck	1000
XAV-939	Tankyrase-1 and -2	E. Differentiating/ epigenetic modifier	Probe		Selleck	100
4-hydroxytamoxifen	Selective estrogen receptor modulator	F. Hormone therapy	Investigational as a gel preparation		Medchem Express	1000
4-hydroxytamoxifen	Selective estrogen receptor modulator	F. Hormone therapy	Investigational as a gel preparation		Medchem Express	100
Abiraterone	P450 17alpha-hydroxylase-17,20-lyase inhibitor	F. Hormone therapy	Approved	S1123	Selleck	500
Abiraterone	P450 17alpha-hydroxylase-17,20-lyase inhibitor	F. Hormone therapy	Approved	S1123	Selleck	50
Aminoglutethimide	Anti-steroid, aromatase inhibitor	F. Hormone therapy	Approved	A9657	Sigma-Aldrich	1000
Aminoglutethimide	Anti-steroid, aromatase inhibitor	F. Hormone therapy	Approved	A9657	Sigma-Aldrich	100
Anastrozole	Aromatase inhibitor	F. Hormone therapy	Approved	HY-14274	Medchem Express	1000
Anastrozole	Aromatase inhibitor	F. Hormone therapy	Approved	HY-14274	Medchem Express	100
Apalutamide	AR antagonist	F. Hormone therapy	Investigational (Ph 2)		Axon Medchem	100
Bicalutamide	Nonsteriodal antiandrogen	F. Hormone therapy	Approved	CT-BIC	ChemieTek	1000
Bicalutamide	Nonsteriodal antiandrogen	F. Hormone therapy	Approved	CT-BIC	ChemieTek	100
Clomifene	Selective estrogen receptor modulator	F. Hormone therapy	Approved	S2561	Selleck	1000
Clomifene	Selective estrogen receptor modulator	F. Hormone therapy	Approved	S2561	Selleck	100
Enzalutamide	AR antagonist	F. Hormone therapy	Approved	HY-70002	Medchem Express	1000
Enzalutamide	AR antagonist	F. Hormone therapy	Approved	HY-70002	Medchem Express	100
Exemestane	Aromatase inhibitor	F. Hormone therapy	Approved	HY-13632	Medchem Express	1000
Exemestane	Aromatase inhibitor	F. Hormone therapy	Approved	HY-13632	Medchem Express	100
Finasteride	type II 5-alpha reductase inhibitor	F. Hormone therapy	Approved	3293	Tocris Biosciences	1000
Finasteride	type II 5-alpha reductase inhibitor	F. Hormone therapy	Approved	3293	Tocris Biosciences	100
Flutamide	Nonsteroidal antiandrogen	F. Hormone therapy	Approved	4094	Tocris Biosciences	1000
Flutamide	Nonsteroidal antiandrogen	F. Hormone therapy	Approved	4094	Tocris Biosciences	100
Fulvestrant	Estrogen receptor antagonist	F. Hormone therapy	Approved	S1191	Selleck	100
Fulvestrant	Estrogen receptor antagonist	F. Hormone therapy	Approved	S1191	Selleck	10
Goserelin	Gonadotropin releasing hormone superagonist	F. Hormone therapy	Approved	HY-13673A	Medchem Express	1000
Goserelin	Gonadotropin releasing hormone superagonist	F. Hormone therapy	Approved	HY-13673A	Medchem Express	100
Lasofoxifene	Selective estrogen receptor modulator	F. Hormone therapy	Approved	sc-211721	Santa Cruz Biotechnology	100
Lasofoxifene	Selective estrogen receptor modulator	F. Hormone therapy	Approved	sc-211721	Santa Cruz Biotechnology	10
Letrozole	Aromatase inhibitor	F. Hormone therapy	Approved	HY-14248	Medchem Express	1000
Letrozole	Aromatase inhibitor	F. Hormone therapy	Approved	HY-14248	Medchem Express	100
Megestrol acetate	Progestogen	F. Hormone therapy	Approved	HY-13676	Medchem Express	1000
Megestrol acetate	Progestogen	F. Hormone therapy	Approved		Medchem Express	100
Nilutamide	Nonsteroidal antiandrogen	F. Hormone therapy	Approved	sc-203644	Santa Cruz Biotechnology	1000
Nilutamide	Nonsteroidal antiandrogen	F. Hormone therapy	Approved	sc-203644	Santa Cruz Biotechnology	100
ODM-201	AR antagonist	F. Hormone therapy	Investigational (Ph 3)		Medchem Express	25
Orteronel	CYP17A1, androgen synth inhib.	F. Hormone therapy	Investigational (Ph 3)		Selleck	1000
Orteronel	CYP17A1, androgen synth inhib.	F. Hormone therapy	Investigational (Ph 3)		Selleck	100
Raloxifene	Selective estrogen receptor modulator	F. Hormone therapy	Approved	HY-13738A	Medchem Express	1000

Raloxifene	Selective estrogen receptor modulator	F. Hormone therapy	Approved	HY-13738A	Medchem Express	100
Tamoxifen	Estrogen receptor antagonist	F. Hormone therapy	Approved	HY-13757	Medchem Express	1000
Tamoxifen	Estrogen receptor antagonist	F. Hormone therapy	Approved	HY-13757	Medchem Express	100
Toremifene	selective estrogen receptor modulator	F. Hormone therapy	Approved	sc-253712	Santa Cruz Biotechnology	1000
Toremifene	selective estrogen receptor modulator	F. Hormone therapy	Approved	sc-253712	Santa Cruz Biotechnology	100
A-1155463	BCL-XL inhibitor	G. Apoptotic modulator	Probe		ChemieTek	1000
A-1155463	BCL-XL inhibitor	G. Apoptotic modulator	Probe		ChemieTek	100
A-1210477	MCL-1 inhibitor	G. Apoptotic modulator	Probe		Active Biochem	5000
A-1210477	MCL-1 inhibitor	G. Apoptotic modulator	Probe		Active Biochem	500
A-1331852	Bcl-XL inhibitor	G. Apoptotic modulator	Probe		ChemieTek	100
A-1331852	Bcl-XL inhibitor	G. Apoptotic modulator	Probe		ChemieTek	10
AMG-232	MDM2 inhibitor	G. Apoptotic modulator	Investigational (Ph 2)		ChemieTek	1000
AMG-232	MDM2 inhibitor	G. Apoptotic modulator	Investigational (Ph 2)		ChemieTek	100
APR-246	p53 activator, thioredoxin reductase 1 inhibitor	G. Apoptotic modulator	Investigational (Ph 1)		Tocris Biosciences	1000
APR-246	p53 activator, thioredoxin reductase 1 inhibitor	G. Apoptotic modulator	Investigational (Ph 1)		Tocris Biosciences	100
AT 101	Bcl-2 family inhibitor	G. Apoptotic modulator	Investigational (Ph 2)		Selleck	10000
AT 101	Bcl-2 family inhibitor	G. Apoptotic modulator	Investigational (Ph 2)		Selleck	1000
AT-406	XIAP, cIAP1, cIAP2 inhibitor	G. Apoptotic modulator	Investigational (Ph 1)		Selleck	1000
AT-406	XIAP, cIAP1, cIAP2 inhibitor	G. Apoptotic modulator	Investigational (Ph 1)		Selleck	100
Birinapant	IAPs, SMAC mimetic	G. Apoptotic modulator	Investigational (Ph 2)		ChemieTek	100
Birinapant	IAPs, SMAC mimetic	G. Apoptotic modulator	Investigational (Ph 2)		ChemieTek	10
Eltanexor	XPO1/CRM1 inhibitor	G. Apoptotic modulator	Investigational (Ph 2)		Medchem Express	1000
Eltanexor	XPO1/CRM1 inhibitor	G. Apoptotic modulator	Investigational (Ph 2)		Medchem Express	100
Idasanutlin	p53-MDM2 inhibitor	G. Apoptotic modulator	Investigational (Ph 3)		Medchem Express	1000
Idasanutlin	p53-MDM2 inhibitor	G. Apoptotic modulator	Investigational (Ph 3)		Medchem Express	100
Navitoclax	Bcl-2/Bcl-xL inhibitor	G. Apoptotic modulator	Investigational (Ph 2)		Selleck	1000
Navitoclax	Bcl-2/Bcl-xL inhibitor	G. Apoptotic modulator	Investigational (Ph 2)		Selleck	100
Necrostatin 2	Necroptosis inhibitor	G. Apoptotic modulator	Probe		Medchem Express	1000
Necrostatin 2	Necroptosis inhibitor	G. Apoptotic modulator	Probe		Medchem Express	100
NVP-CGM097	p53-MDM2 inhibitor	G. Apoptotic modulator	Investigational (Ph 1)		Medchem Express	2500
NVP-CGM097	p53-MDM2 inhibitor	G. Apoptotic modulator	Investigational (Ph 1)		Medchem Express	250
NVP-LCL161	IAPs, SMAC mimetic	G. Apoptotic modulator	Investigational (Ph 2)		ChemieTek	2500
NVP-LCL161	IAPs, SMAC mimetic	G. Apoptotic modulator	Investigational (Ph 2)		ChemieTek	250
PAC-1	procaspase-3 activator	G. Apoptotic modulator	Investigational (Ph 1)		Selleck	1000
PAC-1	procaspase-3 activator	G. Apoptotic modulator	Investigational (Ph 1)		Selleck	100
S-63845	MCL-1 inhibitor	G. Apoptotic modulator	Probe		ChemieTek	100
S-63845	MCL-1 inhibitor	G. Apoptotic modulator	Probe		ChemieTek	10
Sabutoclax	pan-Bcl-2 family inhibitor	G. Apoptotic modulator	Probe		Selleck	250
SAR405838	MDM2 inhibitor	G. Apoptotic modulator	Investigational (Ph 1)		Selleck	1000
SAR405838	MDM2 inhibitor	G. Apoptotic modulator	Investigational (Ph 1)		Selleck	100
Selinexor	XPO1/CRM1 inhibitor	G. Apoptotic modulator	Investigational (Ph 2)		Selleck	1000
Selinexor	XPO1/CRM1 inhibitor	G. Apoptotic modulator	Investigational (Ph 2)		Selleck	100
Sepantronium bromide	Survivin inhibitor	G. Apoptotic modulator	Investigational (Ph 2)		Selleck	1000
Sepantronium bromide	Survivin inhibitor	G. Apoptotic modulator	Investigational (Ph 2)		Selleck	100
Venetoclax	Bcl-2-selective inhibitor	G. Apoptotic modulator	Approved (US)	CT-A199-2	ChemieTek	100
Venetoclax	Bcl-2-selective inhibitor	G. Apoptotic modulator	Approved (US)	CT-A199-2	ChemieTek	10
Verdinexor	XPO1/CRM1 inhibitor	G. Apoptotic modulator	Investigational (Ph 1)		Medchem Express	100
Verdinexor	XPO1/CRM1 inhibitor	G. Apoptotic modulator	Investigational (Ph 1)		Medchem Express	10
WEHI-539	Bcl-XL inhibitor	G. Apoptotic modulator	Probe		Medchem Express	250
WEHI-539	Bcl-XL inhibitor	G. Apoptotic modulator	Probe		Medchem Express	25
Atorvastatin	HMG CoA reductase inhibitor	H. Metabolic modifier	Approved	CT-ATOR	ChemieTek	1000
Atorvastatin	HMG CoA reductase inhibitor	H. Metabolic modifier	Approved	CT-ATOR	ChemieTek	100
AVN944	IMPDH inhibitor	H. Metabolic modifier	Investigational (Ph 2)		ChemieTek	100

AZD3965	MCT1 inhibitor	H. Metabolic modifier	Investigational (Ph 1)		ChemieTek	100
AZD3965	MCT1 inhibitor	H. Metabolic modifier	Investigational (Ph 1)		ChemieTek	10
CPI-613	pyruvate dehydrogenase, alpha-ketoglutarate dehydrogenase inhibitor	H. Metabolic modifier	Investigational (Ph 2)		Selleck	1000
CPI-613	pyruvate dehydrogenase, alpha-ketoglutarate dehydrogenase inhibitor	H. Metabolic modifier	Investigational (Ph 2)		Selleck	100
Daporinad	NAMPT inhibitor	H. Metabolic modifier	Investigational (Ph 2)		Axon Medchem	100
Daporinad	NAMPT inhibitor	H. Metabolic modifier	Investigational (Ph 2)		Axon Medchem	10
Disulfiram (+Cu 2:1)	alcohol dehydrogenase inhibitor	H. Metabolic modifier	Approved (non-oncology)		Selleck	5000
Disulfiram (+Cu 2:1)	alcohol dehydrogenase inhibitor	H. Metabolic modifier	Approved (non-oncology)		Selleck	500
Erastin	VDAC inhibitor, induces ferroptosis	H. Metabolic modifier	Probe		Medchem Express	1000
Erastin	VDAC inhibitor, induces ferroptosis	H. Metabolic modifier	Probe		Medchem Express	100
Lovastatin	HMG-CoA reductase inhibitor	H. Metabolic modifier	approved (non-oncology	S2061	Selleck	1000
Lovastatin	HMG-CoA reductase inhibitor	H. Metabolic modifier	approved (non-oncology	S2061	Selleck	100
Metformin	AMPK activator	H. Metabolic modifier	Approved (non-oncolog	2864	Tocris Biosciences	10000
Metformin	AMPK activator	H. Metabolic modifier	Approved (non-oncolog	2864	Tocris Biosciences	1000
Methotrexate	Antimetabolite; Anti-folate agent	H. Metabolic modifier	Approved	S1210	Selleck	500
Methotrexate	Antimetabolite; Anti-folate agent	H. Metabolic modifier	Approved	S1210	Selleck	50
Pemetrexed	Dihydrofolate reductase inhibitor	H. Metabolic modifier	Approved	P-7177	LC Laboratories	10000
Pemetrexed	Dihydrofolate reductase inhibitor	H. Metabolic modifier	Approved		LC Laboratories	1000
Pevonedistat	NAE inhibitor	H. Metabolic modifier	Investigational (Ph 1)		ChemieTek	1000
Pevonedistat	NAE inhibitor	H. Metabolic modifier	Investigational (Ph 1)		ChemieTek	100
Pravastatin	HMG CoA reductase inhibitor	H. Metabolic modifier	Approved (non-oncolog	2318	Tocris Biosciences	10000
Pravastatin	HMG CoA reductase inhibitor	H. Metabolic modifier	Approved (non-oncolog	2318	Tocris Biosciences	1000
RSL3	GPX4 inhibitor, induces ferroptosis	H. Metabolic modifier	Probe		Medchem Express	1000
RSL3	GPX4 inhibitor, induces ferroptosis	H. Metabolic modifier	Probe		Medchem Express	100
Simvastatin	HMG CoA reductase inhibitor	H. Metabolic modifier	Approved (non-oncolog	S6196	Sigma-Aldrich	1000
Simvastatin	HMG CoA reductase inhibitor	H. Metabolic modifier	Approved (non-oncolog	S6196	Sigma-Aldrich	100
TH588	MTH1 inhibitor	H. Metabolic modifier	Probe		Selleck	2500
TH588	MTH1 inhibitor	H. Metabolic modifier	Probe		Selleck	250
Triapine	ribonucleotide reductase inhibitor	H. Metabolic modifier	Investigational (Ph 2)		Selleck	1000
Triapine	ribonucleotide reductase inhibitor	H. Metabolic modifier	Investigational (Ph 2)		Selleck	100
URB597	FAAH inhibitor	H. Metabolic modifier	Investigational (Ph 1)		Selleck	100
URB597	FAAH inhibitor	H. Metabolic modifier	Investigational (Ph 1)		Selleck	10
Filanesib	KSP/Eg5 inhibitor	I. Kinesin inhibitor	Investigational (Ph 2)		Medchem Express	100
Filanesib	KSP/Eg5 inhibitor	I. Kinesin inhibitor	Investigational (Ph 2)		Medchem Express	10
GSK923295	CENP-E inhibitor	I. Kinesin inhibitor	Investigational (Ph 1)		Medchem Express	1000
GSK923295	CENP-E inhibitor	I. Kinesin inhibitor	Investigational (Ph 1)		Medchem Express	100
Litronesib	Eg5 inhibitor	I. Kinesin inhibitor	Investigational (Ph 2)		Medchem Express	100
Litronesib	Eg5 inhibitor	I. Kinesin inhibitor	Investigational (Ph 2)		Medchem Express	10
SB 743921	Mitotic inhibitor. Eg5/KSP inhibitor	I. Kinesin inhibitor	Investigational (Ph 2)		Selleck	10
SB 743921	Mitotic inhibitor. Eg5/KSP inhibitor	I. Kinesin inhibitor	Investigational (Ph 2)		Selleck	1
Celecoxib	Selective COX-2 inhibitor	J. NSAID	Approved	S1261	Selleck	1000
Celecoxib	Selective COX-2 inhibitor	J. NSAID	Approved	S1261	Selleck	100
BIIB021	HSP90 inhibitor	K. HSP inhibitor	Investigational (Ph 2)		Selleck	1000
BIIB021	HSP90 inhibitor	K. HSP inhibitor	Investigational (Ph 2)		Selleck	100
CUDC-305	HSP90 inhibitor	K. HSP inhibitor	Investigational (Ph 1)		ChemieTek	1000
CUDC-305	HSP90 inhibitor	K. HSP inhibitor	Investigational (Ph 1)		ChemieTek	100
Ganetespib	HSP90 inhibitor	K. HSP inhibitor	Investigational (Ph 3)		Selleck	100
Ganetespib	HSP90 inhibitor	K. HSP inhibitor	Investigational (Ph 3)		Selleck	10
Luminespib	HSP90 inhibitor	K. HSP inhibitor	Investigational (Ph 2)		ChemieTek	10
Onalespib	HSP90 inhibitor	K. HSP inhibitor	Investigational (Ph 2)		Medchem Express	250
Onalespib	HSP90 inhibitor	K. HSP inhibitor	Investigational (Ph 2)		Medchem Express	25
VER 155008	HSP70 inhibitor	K. HSP inhibitor	Probe		Axon Medchem	1000
VER 155008	HSP70 inhibitor	K. HSP inhibitor	Probe		Axon Medchem	100

Bortezomib	Proteasome inhibitor (26S subunit)	L. Protease/proteasome inhibitor	Approved	CT-BZ001	ChemieTek	100
Bortezomib	Proteasome inhibitor (26S subunit)	L. Protease/proteasome inhibitor	Approved	CT-BZ001	ChemieTek	10
Carfilzomib	Proteasome inhibitor (20S subunit)	L. Protease/proteasome inhibitor	Approved	CT-CARF	ChemieTek	100
Carfilzomib	Proteasome inhibitor (20S subunit)	L. Protease/proteasome inhibitor	Approved	CT-CARF	ChemieTek	10
Ixazomib	20S proteasome inhibitor	L. Protease/proteasome inhibitor	Approved	S2180	Selleck	100
Ixazomib	20S proteasome inhibitor	L. Protease/proteasome inhibitor	Approved	S2180	Selleck	10
ONX-0914	LMP7 (immunoproteasome)	L. Protease/proteasome inhibitor	Probe		Selleck	1000
ONX-0914	LMP7 (immunoproteasome)	L. Protease/proteasome inhibitor	Probe		Selleck	100
Oprozomib	proteasome (20 S) inhibitor	L. Protease/proteasome inhibitor	Investigational (Ph 1)		ChemieTek	250
Oprozomib	proteasome (20 S) inhibitor	L. Protease/proteasome inhibitor	Investigational (Ph 1)		ChemieTek	25
Tosedostat	Aminopeptidase inhibitor	L. Protease/proteasome inhibitor	Investigational (Ph 3)		Tocris Biosciences	1000
Tosedostat	Aminopeptidase inhibitor	L. Protease/proteasome inhibitor	Investigational (Ph 3)		Tocris Biosciences	100
VLX1570	proteasome deubiquitinase inhibitor	L. Protease/proteasome inhibitor	Investigational (Ph 2)		Medchem Express	1000
VLX1570	proteasome deubiquitinase inhibitor	L. Protease/proteasome inhibitor	Investigational (Ph 2)		Medchem Express	100
Glasdegib	Smo inhibitor	M. Hedgehog inhibitor	Investigational (Ph 2)		Medchem Express	10
Saridegib	Smothened (Hh) inhib	M. Hedgehog inhibitor	Investigational (Ph 2)		Active Biochem	100
Sonidegib	Smothened (Hh) inhib	M. Hedgehog inhibitor	Approved	CT-LDE225-:	ChemieTek	1000
Sonidegib	Smothened (Hh) inhib	M. Hedgehog inhibitor	Approved		ChemieTek	100
Taladegib	Smothened (Hh) inhib	M. Hedgehog inhibitor	Investigational (Ph 2)		Medchem Express	1000
Taladegib	Smothened (Hh) inhib	M. Hedgehog inhibitor	Investigational (Ph 2)		Medchem Express	100
Vismodegib	Smothened (Hh) inhibitor	M. Hedgehog inhibitor	Approved		LC Laboratories	1000
Vismodegib	Smothened (Hh) inhibitor	M. Hedgehog inhibitor	Approved	V-4050	LC Laboratories	100
1-methyl-D-tryptophan	Indolamine 2,3-dioxygenase 1 and 2 inhibitor	X. Other	Investigational (Ph 2)	452483	Sigma-Aldrich	10000
1-methyl-D-tryptophan	Indolamine 2,3-dioxygenase 1 and 2 inhibitor	X. Other	Investigational (Ph 2)	452483	Sigma-Aldrich	1000
Anagrelide	PDE-3, PLA2 inhibitor	X. Other	Approved	2432	Tocris Biosciences	1000
Anagrelide	PDE-3, PLA2 inhibitor	X. Other	Approved	2432	Tocris Biosciences	100
BCI	Dusp6 inhibitor	X. Other	Probe		Sigma-Aldrich	5000
BCI	Dusp6 inhibitor	X. Other	Probe		Sigma-Aldrich	500
BRD7116	Leukemic stem cell inhibitor	X. Other	Probe		Medchem Express	1000
BRD7116	Leukemic stem cell inhibitor	X. Other	Probe		Medchem Express	100
Cilengitide	alphaVbeta3 integrin inhibitor	X. Other	Investigational (Ph 3)		Selleck	1000
Cilengitide	alphaVbeta3 integrin inhibitor	X. Other	Investigational (Ph 3)		Selleck	100
Darapladib	lipoprotein-associated phospholipase A2 inhibitor	X. Other	Investigational (Ph 3)		Selleck	100
Deferoxamine	Iron chelator	X. Other	Approved (non-oncolog	D9533	Sigma-Aldrich	10000
Deferoxamine	Iron chelator	X. Other	Approved (non-oncolog	D9533	Sigma-Aldrich	1000
Digoxin	Cardiac glycoside	X. Other	Approved (non-oncolog	HY-B1049	Medchem Express	100
Digoxin	Cardiac glycoside	X. Other	Approved (non-oncology)		Medchem Express	10
E7820	Integrin alpha2 expression inhibitor	X. Other	Investigational (Ph 2)		Medchem Express	5000
E7820	Integrin alpha2 expression inhibitor	X. Other	Investigational (Ph 2)		Medchem Express	500
Fingolimod	S1PR antagonist	X. Other	Approved	F-4633	LC Laboratories	1000
Fingolimod	S1PR antagonist	X. Other	Approved		LC Laboratories	100
Galiellalactone	STAT3-DNA interaction inhibitor	X. Other	Probe		Santa Cruz Biotechnology	2500
Galiellalactone	STAT3-DNA interaction inhibitor	X. Other	Probe		Santa Cruz Biotechnology	250
GSK2830371	Wip1 inhibitor	X. Other	Probe		ChemieTek	500
GSK2830371	Wip1 inhibitor	X. Other	Probe		ChemieTek	50
Itraconazole	antifungal, hedgehog signaling inhibitor	X. Other	Approved (non-oncolog	S2476	Selleck	500
Itraconazole	antifungal, hedgehog signaling inhibitor	X. Other	Approved (non-oncolog	S2476	Selleck	50
JPH203	LAT1 inhibitor	X. Other	Probe		Medchem Express	500
Marimastat	MMP-9, MMP-1, MMP-2, MMP-14, MMP-7 inhibitor	X. Other	Investigational (Ph 3)		Selleck	1000
Marimastat	MMP-9, MMP-1, MMP-2, MMP-14, MMP-7 inhibitor	X. Other	Investigational (Ph 3)		Selleck	100
Mepacrine	Unclear. PLA2 inhibitor. NF-kB inhibitor, p53 activator	X. Other	Approved	Q3251	Sigma-Aldrich	10000
Mepacrine	Unclear. PLA2 inhibitor. NF-kB inhibitor, p53 activator	X. Other	Approved	Q3251	Sigma-Aldrich	1000
Miltefosine	Antimicrobial, inhibits PI3K/AKT	X. Other	Approved	S3056	Selleck	10000

Miltefosine	Antimicrobial, inhibits PI3K/AKT	X. Other	Approved		Selleck	1000
MK-0752	gamma-secretase/notch inhibitor	X. Other	Investigational (Ph 2)		Selleck	100
MK-0752	gamma-secretase/notch inhibitor	X. Other	Investigational (Ph 2)		Selleck	10
ML323	USP1-UAF1 inhibitor	X. Other	Probe		Selleck	1000
ML323	USP1-UAF1 inhibitor	X. Other	Probe		Selleck	100
MST-312	Telomerase inhibitor	X. Other	Probe		Sigma-Aldrich	1000
MST-312	Telomerase inhibitor	X. Other	Probe		Sigma-Aldrich	100
Napabucasin	CSC inhibitor, STAT3 mediated	X. Other	Investigational (Ph 3)		Medchem Express	2000
Napabucasin	CSC inhibitor, STAT3 mediated	X. Other	Investigational (Ph 3)		Medchem Express	200
NMS-873	p97/VCP inhibitor	X. Other	Probe		Selleck	1000
NMS-873	p97/VCP inhibitor	X. Other	Probe		Selleck	100
NVP-LGK974	PORCN inhibitor	X. Other	Investigational (Ph 1)		Selleck	1000
NVP-LGK974	PORCN inhibitor	X. Other	Investigational (Ph 1)		Selleck	100
NVP-SHP099	SHP2 inhibitor	X. Other	Probe		Medchem Express	1000
NVP-SHP099	SHP2 inhibitor	X. Other	Probe		Medchem Express	100
Omaveloxolone	Nrf2 activator	X. Other	Investigational (Ph 2)		Selleck	1000
Omaveloxolone	Nrf2 activator	X. Other	Investigational (Ph 2)		Selleck	100
PF-3845	FAAH inhibitor	X. Other	Probe		Selleck	1000
PF-3845	FAAH inhibitor	X. Other	Probe		Selleck	100
Pilocarpine	Non-selective muscarinic receptor agonist	X. Other	Approved	0694	Tocris Biosciences	4000
Pilocarpine	Non-selective muscarinic receptor agonist	X. Other	Approved	0694	Tocris Biosciences	400
Plerixafor	CXCR4 antagonist	X. Other	Approved	HY-50912	Medchem Express	10000
Plerixafor	CXCR4 antagonist	X. Other	Approved	HY-50912	Medchem Express	1000
Rigosertib	Ras-Raf interaction inhibitor, contaminated by microtubile depolymeriz	X. Other	Investigational (Ph 3)		Medchem Express	1000
Rigosertib	Ras-Raf interaction inhibitor, contaminated by microtubile depolymeriz	X. Other	Investigational (Ph 3)		Medchem Express	100
Salinomycin	Ionophore	X. Other	Veterinary approval	HY-17439	Medchem Express	5000
Salinomycin	Ionophore	X. Other	Veterinary approval	HY-17439	Medchem Express	500
SH-4-54	STAT3 inhibitor	X. Other	Probe		Selleck	2500
SH-4-54	STAT3 inhibitor	X. Other	Probe		Selleck	250
Tarenflurbil	Gamma-secretase inhibitor	X. Other	Investigational (Ph 3)		Cayman Chemical Company	1000
Tarenflurbil	Gamma-secretase inhibitor	X. Other	Investigational (Ph 3)		Cayman Chemical Company	100
TIC10	ERK & AKT inhibitor, TRAIL inducer	X. Other	Investigational (Ph 2)		Selleck	2500
TIC10	ERK & AKT inhibitor, TRAIL inducer	X. Other	Investigational (Ph 2)		Selleck	250
TRAM-34	intermediate-conductance Ca2+-activated K+ channel inh.	X. Other	Probe		Selleck	100
TRAM-34	intermediate-conductance Ca2+-activated K+ channel inh.	X. Other	Probe		Selleck	10
Varespladib	Secretory phospholipase A2 inhibitor	X. Other	Investigational (Ph 2)		ChemieTek	1000
Varespladib	Secretory phospholipase A2 inhibitor	X. Other	Investigational (Ph 2)		ChemieTek	100
