# Supplementary material for: Drug repositioning strategy for the identification of novel telomere‐damaging agents: A role for NAMPT inhibitors
Source: Aging Cell. 2023 Oct 19;22(11):e13944. doi: 10.1111/acel.13944 (PMC10652301; doi:10.1111/acel.13944)
Supplement: Supplementary file 5 — Table S3. [file ACEL-22-e13944-s001.pdf]

Table S3 - List of primers used in the work

qPCR	Sequence (5' – 3')
TRF2 FW	CATGCAGGCTTTGCTTGTCA
TRF2 RV	CTGCATAACCCGCAGCAATC
Actin FW	AGCACTGTGTTGGCGTACAG
Actin RV	TCCCTGGAGAAGAGCTACGA
36B4-FW	CAGCAAGTGGGAAGGTGTAATCC
36B4-RV	CCCATTCTATCATCAACGGGTACAA
Telo-8oxoG FW	CGGTTTGTTTGGGTTTGGGTTTGGGTTTGGGTTTGGGTT
Telo-8oxoG RV	GGCTTGCCTTACCCTTACCCTTACCCTTACCCTTACCCT

qPCR-ChIP	
Sub-Telo Chr 2 FW	CCCAAACCCTAACCCTAAAA
Sub-Telo Chr 2 RV	CTTCCTGTTTGCAGCACTGA
Sat II FW	TCGCATAGAATCGAATGGAA
Sat II RV	GCATTCGAGTCCGTGGA
k-Ras G-rich prom FW	GTACGCCCGTCTGAAGAAGA
k-Ras G-rich prom RV	GAGCACACCGATGAGTTCGG
